# Photochemical reduction of acylimidazolium salts

**DOI:** 10.3762/bjoc.21.153

**Published:** 2025-09-25

**Authors:** Michael Jakob, Nick Bechler, Hassan Abdelwahab, Fabian Weber, Janos Wasternack, Leonardo Kleebauer, Jan P Götze, Matthew N Hopkinson

**Affiliations:** 1 Institut für Chemie und Biochemie, Freie Universität Berlin, Arnimallee 20, 14195 Berlin, Germany; 2 Institut für Chemie und Biochemie, Freie Universität Berlin, Arnimallee 22, 14195 Berlin, Germany; 3 School of Natural and Environmental Sciences, Newcastle University, Bedson Building, Newcastle upon Tyne, NE1 7RU, UK

**Keywords:** carbenes, carbonyls, NHCs, photochemistry, reduction

## Abstract

Light-mediated methodologies for the reduction of acylazolium species generated during *N*-heterocyclic carbene (NHC)-catalyzed reactions have been developed. Employing the simple amine, DIPEA, as the terminal reductant, products resulting from overall 2-electron or 4-electron-reduction processes could be obtained using either a photocatalytic approach under blue light irradiation or directly under UV-A light irradiation without an additional photocatalyst. Moreover, under the same photocatalyst-free conditions, UV-A-light-mediated reduction could be achieved using triethylsilane as the only reductant with subsequent desilylation and NHC elimination with fluoride delivering the corresponding aldehyde product.

## Introduction

**Figure 1:**
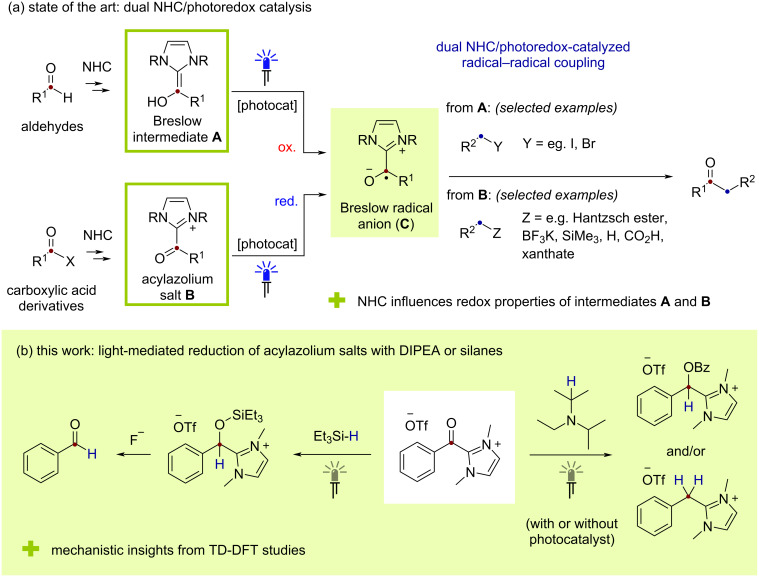
(a) Combining *N*-heterocyclic carbene (NHC) organocatalysis with photoredox catalysis for radical–radical coupling reactions. (b) This work: light-mediated reduction of acylimidazolium species **1** with the tertiary amine DIPEA or the simple silane HSiEt_3_.

The introduction and exploration of *N*-heterocyclic carbenes (NHCs) ranks among the most important developments in chemistry research of the last 30 years [1–3]. In addition to their numerous valuable roles as ligands, including for important transition-metal complexes such as the Grubbs’ second-generation metathesis catalyst, NHCs are now also well-established as organocatalysts. With the first application pre-dating the unambiguous characterization of a free NHC by nearly 50 years, NHCs can facilitate numerous synthetically attractive transformations of carbonyl substrates with umpolung processes of aldehydes such as the benzoin condensation and Stetter reaction being particularly well studied [4–11]. In these processes, addition of the NHC to the aldehyde followed by proton transfer generates the enamine-like Breslow intermediate **A** (Figure 1a), in which the formerly electrophilic carbonyl carbon reacts as a nucleophilic center. In this way, the traditional reactivity profile of the carbonyl group is transiently inverted, and unconventional product classes are generated. Alternatively, addition/elimination of the NHC to a suitably electrophilic substrate at the carboxylic acid oxidation level provides an acylazolium species **B**, which typically reacts directly with nucleophiles or may first be transformed into the corresponding enolate derivative. Regardless of the individual pathway, NHC-catalyzed reactions of this type offer many synthetic advantages while the wide availability of chiral NHCs can also allow for high levels of enantioselectivity.

As effective enamine and active ester derivatives, Breslow and acylazolium intermediates **A** and **B** typically react via classical two-electron polar mechanisms, however, recent research has demonstrated that NHCs are also capable of stabilizing radical or excited-state species [12–13]. In 2020, our group reported the concept of photo-NHC catalysis where direct excitation of acylazolium intermediates generated from *o*-toluoyl fluoride substrates with UV-A light resulted in a novel catalytic photoenolization/Diels–Alder (PEDA) reaction [14–15]. In this process, the NHC fragment present in the acylazolium species influences the absorption wavelength and fundamental photochemical reactivity of the C=O bond, enabling a “ketone-like” photoreaction from otherwise unsuitable carboxylic acid derivative substrates [16]. Over the last few years, a wide range of valuable NHC-catalyzed transformations have also been developed that incorporate redox steps. As an enamine species, single-electron oxidation of a Breslow intermediate is comparatively favored with the resulting open shell species **C** benefitting from additional stabilization by virtue of electron delocalization onto the NHC-derived azolium ring [17–20]. Similarly, the cationic azolium fragment in acylazolium salts can effectively lower the carbonyl reduction potential relative to the starting material with single-electron reduction delivering the same stabilized radical **C**. Beginning with a seminal report by di Rocco and Rovis in 2012 [21], the combination of NHC and photoredox catalysis has recently been the subject of intense research activity [22–30]. Employing the latter reductive manifold with carboxylic acid derivatives, numerous coupling processes affording ketone products have been developed. Since the initial report from Scheidt and co-workers using 4-alkyl-substituted Hantzsch esters as coupling partners [31–36], several alkyl radical sources have been employed including carboxylic acids [37], xanthates [38], electron-rich toluene or heteroatom-substituted species [39–42], organoboron compounds [43–44] and organosilanes [43,45]. Moreover, three-component radical relay processes employing styrene derivatives have also been widely studied. In contrast to these numerous reports with carbon-based alkyl radicals, dual NHC/photoredox-mediated coupling processes between carboxylic acid derivatives and other classes of radical are lacking [22–30]. In particular, to the best of our knowledge, formal reduction reactions of carboxylic acid derivatives involving hydrogen-atom transfer have not been reported despite the fundamental importance of carbonyl reduction processes in organic synthesis [46]. Here, we report the results of our investigation into such reactions using an acylazolium salt derived from benzoic acid as a model substrate (Figure 1b). This led to the successful development of a novel dual NHC/light-mediated reduction process using either the simple tertiary amine, NEt(iPr)_2_ (DIPEA) or the widely available silane HSiEt_3_, as the only reductant. Moreover, interesting insights into the reaction mechanism supported by density functional theory (DFT) calculations were obtained.

## Results and Discussion

### Photoreduction of 2-benzoylimidazolium triflate with diisopropylethylamine

#### Initial investigations

**Figure 2:**
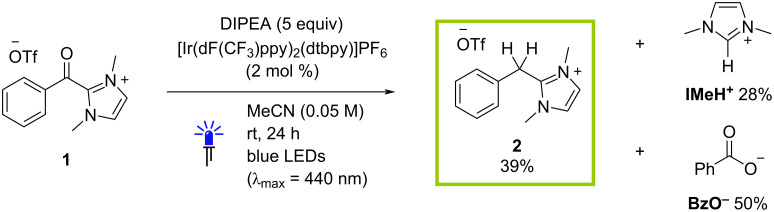
Initial test reaction employing [Ir(dF(CF_3_)ppy)_2_(dtbpy)]PF_6_ as a photocatalyst in the presence of DIPEA (5 equiv). ^1^H NMR yields shown using CH_2_Br_2_ as an internal standard and assuming a one-to-one molar ratio of starting material **1** to products.

To investigate the potential for the light-mediated reduction of acylazolium salts, compound **1** was prepared as a representative substrate in two steps from benzoyl chloride, imidazole and methyl trifluoromethanesulfonate. In an initial reaction, this species was reacted under photoredox conditions in the presence of [Ir(dF(CF_3_)ppy)_2_(dtbpy)]PF_6_ (2 mol %, dF(CF_3_)ppy = 3,5-difluoro-2-[5-(trifluoromethyl)-2-pyridine, dtbpy = 4,4′-di-*tert*-butyl-2,2′-dipyridine) as a photocatalyst and the simple tertiary amine diisopropylethylamine (DIPEA, 5 equiv) in MeCN (0.05 M). After 24 h under irradiation with light from blue LEDs (λ_max_ = 440 nm), the crude mixture was concentrated under reduced pressure and analyzed by ^1^H NMR spectroscopy. As shown in Figure 2, complete consumption of **1** was observed under these conditions with concomitant formation of a major new species with a characteristic ^1^H NMR signal at δ = 4.42 ppm (in CDCl_3,_ Table 1, entry 1). Comparison of the spectrum with that of an authentic sample (see Supporting Information File 1) allowed for the unambiguous identification of this product as the 2-benzylimidazolium compound **2**. The formation of this species results from an overall 4-electron-reduction process, indicating that the photocatalytic system with DIPEA as the terminal reductant is capable of facilitating two reduction steps with an initial reaction with the acylazolium starting material being followed by a presumably more challenging second reduction of a less-activated intermediate species. Using CH_2_Br_2_ as an internal reference, a ^1^H NMR yield of 39% was calculated with further analysis of the crude spectrum also indicating the presence of significant amounts of the imidazolium salt **IMeH^+^** (characteristic C2–H signal at δ = 8.94 ppm; 28%) and benzoate species (**BzO**^−^, *ortho*-C–H signals at δ = 7.98 ppm; 50%).

**Table 1:** Survey of reaction conditions for the light-mediated reduction of 2-benzoylimidazolium salt **1** using DIPEA as the terminal reductant.

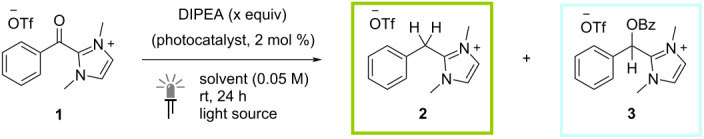						

Entry^a^	Photocatalyst	Equiv of DIPEA	Solvent	Light source (λ_max_)	Yield of **2**^b^	Yield of **3**^b^

1	[Ir(dF(CF_3_)ppy)_2_(dtbpy)]PF_6_	5	MeCN	440 nm	39%	–
2	[Ir(dF(CF_3_)ppy)_2_(dtbpy)]PF_6_	–	MeCN	440 nm	n.d.^c^	n.d.^c^
3^d^	[Ir(dF(CF_3_)ppy)_2_(dtbpy)]PF_6_	5	MeCN	440 nm	–	–
4	–	5	MeCN	440 nm	2%	15%
5	–	5	MeCN	370 nm	38%	9%
6	–	4	MeCN	370 nm	29%	21%
7	–	3	MeCN	370 nm	21%	21%
8	–	2	MeCN	370 nm	24%	17%
9	–	1	MeCN	370 nm	30%	9%
10	–	1	DCM	370 nm	6%	38%
11	–	1	DCE	370 nm	6%	35%
12	–	1	PhCl	370 nm	8%	38%
13	–	1	PhCF_3_	370 nm	11%	24%
14	–	1	acetone	370 nm	14%	24%
15	–	1	THF	370 nm	4%	7%
16	–	1	DMF	370 nm	n.d.^c^	n.d.^c^
17	[Ir(dF(CF_3_)ppy)_2_(dtbpy)]PF_6_	1	MeCN	440 nm	48%	–
18	[Ir(ppy)_2_(dtbpy)]PF_6_	1	MeCN	440 nm	46%	traces
19	4CzIPN	1	MeCN	440 nm	50%	–

^a^Reactions conducted on a 0.1 mmol scale in a dry Schlenk flask. ^b1^H NMR yields using CH_2_Br_2_ as an internal standard and assuming a one-to-one molar ratio of starting material **1** to products. ^c^Complex reaction mixture. ^d^Reaction conducted in the dark.

To confirm the photocatalytic nature of the novel carbonyl reduction process, a series of control reactions were carried out. As expected, conducting the reaction in the absence of the terminal reductant, DIPEA, led to a complex reaction mixture (Table 1, entry 2), while performing the reaction in the dark resulted only in recovered starting material (Table 1, entry 3). To our surprise, however, irradiation of **1** and DIPEA (5 equiv) in the absence of the photocatalyst (λ_max_ = 440 nm) did result in significant consumption of the acylazolium species (11% remaining by ^1^H NMR) with the fully reduced species **2** being observed in trace amounts (^1^H NMR yield = 2%). In addition to these compounds, **IMeH^+^** (45%) and **BzO**^−^ (43%), additional signals consistent with the *O*-benzoylated species **3** were identified. This compound was synthesized independently from benzoyl chloride and 1-methylimidazole (see Supporting Information File 1) with spiking of the crude ^1^H NMR spectrum with the authentic sample confirming the presence of this intermediate reduction product in the reaction mixture. Compound **3** is likely formed in a two-stage process involving initial reduction of the carbonyl group followed by addition/elimination of the resulting alkoxide into a second equivalent of the acylazolium salt **1**; a sequence which also partially explains the generation of the free **IMeH^+^** observed in the crude reaction mixture. Integration of the signals indicated a ^1^H NMR yield of 15% on a one-to-one molar basis relative to the acylazolium species **1**, however, given that two molecules of **1** are required to form one molecule of **3**, this represents an actual chemical yield of 30%. While seemingly less efficient than the photoredox process, the successful generation of reduced species in the absence of the catalyst is a remarkable result that highlights the unique influence of the NHC fragment on the reduction potentials and photoreactivity of acylazolium salts.

#### Survey of reaction conditions

To gain further insight into the photocatalyst-free reduction process, a survey of reaction conditions was conducted. In each case, the ^1^H NMR yields of the reduced species were calculated using CH_2_Br_2_ as an internal standard with results displayed in Table 1. To avoid confusion and facilitate comparisons between different conditions, percentage yields are stated on a one-to-one molar basis with respect to the acylazolium starting material **1** ignoring the influence of reaction stoichiometry. Firstly, the wavelength of the light source (λ_max_) was changed from 440 nm (blue LEDs) to 370 nm (UV-A). This resulted in an overall improvement in the reduction efficiency with the fully reduced species **2** becoming the major product formed in 38% yield alongside the *O*-benzoylated species **3** (9%, Table 1, entry 5). This result is again remarkable in that it appears the simple combination of the tertiary amine DIPEA and UV-A light irradiation is sufficient to effect complete reduction of the acylazolium species. Decreasing the equivalents of DIPEA did not have a significant influence on the overall amount of reduced products formed although the proportion of the fully reduced species **2** relative to **3** generally decreased (Table 1, entries 6–9). Employing just 1 equivalent of DIPEA nevertheless delivered 30% of **2** alongside 9% of **3**. A survey of common organic solvents was then conducted to investigate the influence of the reaction medium. Interestingly, switching from MeCN to the chlorinated solvents DCM, DCE and chlorobenzene led to a decline in the formation of the fully reduced product **2** with *O*-benzoylated species **3** being obtained as the major product (38% in DCM, 35% in DCE, 38% in PhCl cf. 6–8% of **2**, Table 1, entries 10–12). Significant reduction of **1** was also observed in PhCF_3_ (11% of **2**, 24% of **3**, Table 1, entry 13) and acetone (14% of **2**, 24% of **3**, Table 1, entry 14), however, relatively complex reaction mixtures were obtained in either THF (4% of **2**, 7% of **3**, Table 1, entry 15) or DMF (Table 1, entry 16). At this stage, we returned our attention to the photocatalyzed process, testing a selection of photocatalysts in the presence of 1 equivalent of DIPEA under blue light irradiation (λ_max_ = 440 nm). Notably, under these modified conditions, the photocatalyzed reaction with the originally test photocatalyst, [Ir(dF(CF_3_)ppy)_2_(dtbpy)]PF_6_, resulted in clean formation of the fully reduced product **2** in 48% ^1^H NMR yield with no intermediate species **3** being detected (Table 1, entry 17). The related iridium complex [Ir(ppy)_2_(dtbpy)]PF_6_ (ppy = 2-phenylpyridine) delivered **2** in a similar yield of 46% (Table 1, entry 18) while the organic species 1,2,3,5-tetrakis(carbazol-9-yl)-4,6-dicyanobenzene (4CzIPN), which has been commonly employed in dual NHC/photoredox-catalyzed coupling reactions, cleanly provided the fully reduced species in 50% ^1^H NMR yield (Table 1, entry 19).

#### Comments on the reaction mechanism

**Scheme 1:**
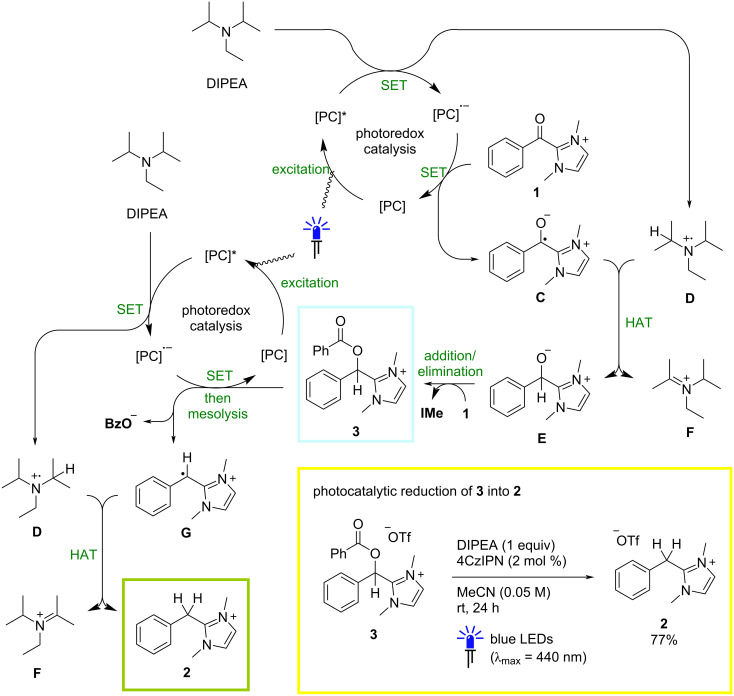
Plausible mechanism for the photocatalytic reduction of benzoylimidazolium salt **1** with DIPEA. [PC] = photocatalyst; SET = single-electron transfer; HAT = hydrogen atom transfer.

At this stage of the study, our attention turned to a consideration of the reaction mechanism. As has been well documented in the photoredox literature [47–53], excitation of a photocatalyst ([PC]) in the presence of DIPEA can result in reductive quenching of the excited state, affording the corresponding photocatalyst radical anion ([PC]^·−^) and the DIPEA radical cation **D** (Scheme 1). Single-electron transfer from [PC]**^·^**^−^ to the benzoylazolium species **1** would then regenerate the ground-state photocatalyst and afford the Breslow radical anion **C**, which could in turn react with **D** in a hydrogen-atom-transfer (HAT) step to form the zwitterionic alkoxide **E** and the oxidized DIPEA iminium ion **F**. Notably, small signals consistent with trace amounts of the alkoxide species **E** were occasionally detected in the crude ^1^H NMR spectra of reactions conducted both in the presence and absence of a photocatalyst (see Supporting Information File 1 for details). Finally, **E** could react in an addition/elimination process with a second molecule of the benzoylazolium starting material **1** to afford the observed *O*-benzoylated species **3**. An analogous reductive quenching cycle where the reduced photocatalyst [PC]**^·^**^−^ instead transfers an electron to compound **3** could explain the formation of the fully reduced species **2**. In this case, subsequent mesolysis would generate the benzyl radical cation **G**, which would deliver **2** following a HAT step with the DIPEA radical cation **D**.

To confirm whether *O*-benzoylated species is indeed an intermediate in the formation of the fully reduced product **2** under photocatalytic conditions, the independently synthesized **3** was irradiated with 1 equivalent of DIPEA and 4CzIPN under the conditions shown in Table 1, entry 16 (Scheme 1, in yellow box). After 24 h at rt, clean conversion into **2** (77% ^1^H NMR yield) was observed, implying that a mechanistic scenario in line with that shown in Scheme 1 is plausible. Given that two molecules of the benzoylazolium starting material are required to form one molecule of **3** and, by extension **2**, the ^1^H NMR yields of 46–50% quoted for the photocatalytic reactions in Table 1, entries 14–16, actually represent a near quantitative chemical conversion into the fully reduced species.

**Scheme 2:**
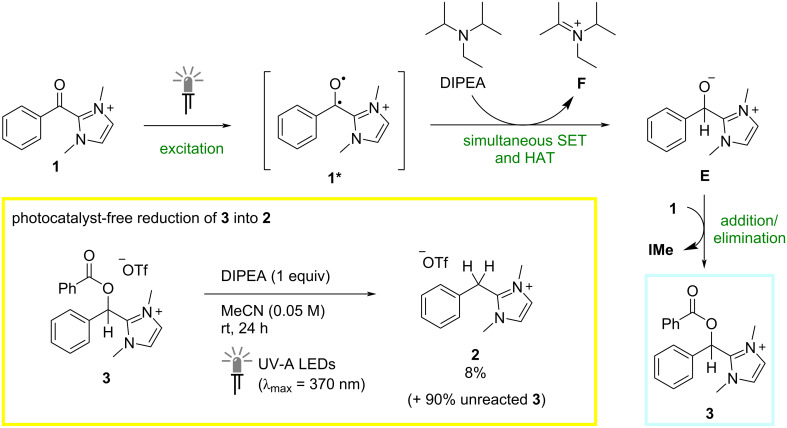
Plausible mechanism for the photocatalyst-free reduction of benzoylimidazolium salt **1** into *O*-benzoylated compound **3** with DIPEA.

The successful reduction of **1** into both **2** and **3** in the absence of a photocatalyst requires an alternative mechanistic explanation. Without a sensitizer present, a question arises regarding the identity of the active light-absorbing species in the reaction mixture. UV–vis spectra of the benzoylazolium starting material **1** at different concentrations in the acetonitrile reaction solvent were accordingly measured. Although exhibiting maximum absorption at wavelengths significantly shorter than that emitted by the light sources used (latest λ_abs,max_ = 271 nm), a significant absorption tail up to ca. λ = 400 nm was observed at higher concentrations, indicating that the starting material could potentially absorb the incident light directly. This rationale is consistent with the increase in reduction efficiency observed upon switching from blue to UV-A light irradiation. Nevertheless, alternative scenarios involving the potential formation of excited donor–acceptor (EDA) complexes between benzoylazolium species **1** and DIPEA were considered. Measurements of the UV–vis spectra in the presence of the amine, however, did not reveal any significant change in the absorption profile. To gain further insight, time-dependent density functional theory (TD-DFT) calculations were carried out (for details of the computational studies, see Supporting Information File 1). In line with the UV–vis studies, comparison of the computed vertical absorption spectrum of the benzoylazolium salt **1** with and without DIPEA did not reveal the formation of an EDA complex. We therefore propose that, even under irradiation with comparatively long wavelength blue light (λ_max_ = 440 nm), the benzoylimidazolium species **1** likely acts as the active absorbing species with its absorption band overlapping sufficiently with the emission front of the employed LEDs. Direct absorption of light by acylazolium species has indeed been previously proposed as a mechanistic pathway in light-mediated NHC-catalyzed coupling reactions [22–30]. The TD-DFT calculations also provided insight into the nature of the subsequent mechanistic steps where the excited benzoylimidazolium 1* is converted to the reduced zwitterionic alkoxide species **E** in the presence of DIPEA (Scheme 2). This requires the transfer of both an electron and a hydrogen atom from the amine to the excited carbonyl species, and analysis of the computed charge distributions indicates that these processes occur simultaneously in what can be best described as a formal hydride ion transfer. Addition/elimination of **E** into the carbonyl group of a second molecule of **1** then generates the *O*-benzoylated species **3**.

**Figure 3:**
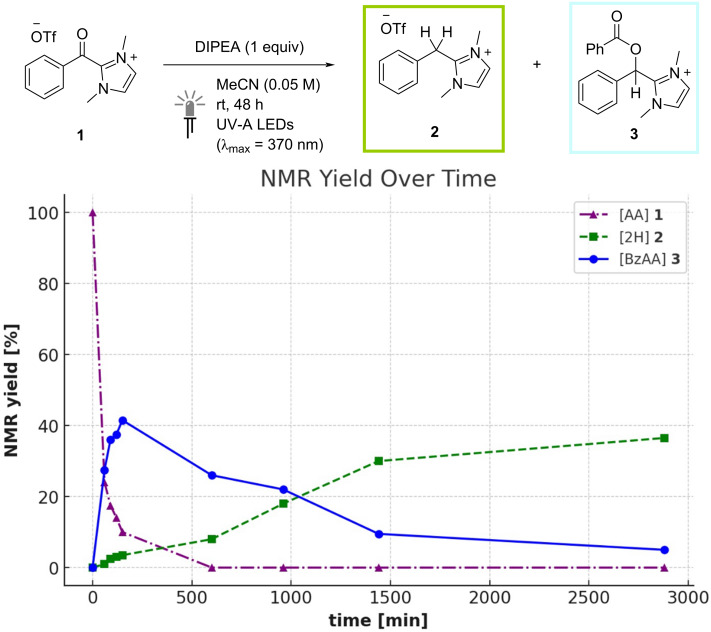
Reduction of 2-benzoylimidazolium triflate (**1**) under photocatalyst-free conditions monitored over 48 h by ^1^H NMR.

Determining a mechanistic rationale for the generation of the fully reduced species **2** under photocatalyst-free conditions is more challenging. The *O*-benzoylated species **3** is expected to be less susceptible to reduction than the benzoylazolium starting material **1** and, with no discernible chromophores present, it is also less likely to absorb the incident light. Indeed, when subjected to the reaction conditions shown in Table 1, entry 9 with 1 equivalent of DIPEA under UV-A light irradiation, only sluggish conversion of independently synthesized **3** into the 2-benzylimidazolium salt **2** was observed (^1^H NMR yield = 8%) with 90% of **3** remaining unreacted after 24 h at rt (Scheme 2, in yellow box). To gain more insight into the reaction course in the absence of a photocatalyst, the reduction of **1** with DIPEA (1 equiv) under UV-A irradiation was followed by ^1^H NMR over the course of 48 h. As shown in Figure 3, at the start of the reaction, rapid consumption of the benzoylazolium starting material was observed with concomitant formation of *O*-benzoylated product **3**. The concentration of this species then slowly decreased over the remaining reaction time along with increasing formation of the fully reduced compound **2**. Taken together, these experiments seem to indicate that the *O*-benzoylated species **3** is indeed an intermediate in the formation of **2** but that the second reduction largely occurs only in concert with the initial reduction of **1**. Further studies would be needed to fully elucidate the active mechanistic pathway in this system, however, potential explanations could include activation of **3** by DIPEA-derived reaction by-products, or invoke the involvement of the strongly reducing DIPEA α-amino radical, which could feasibly be formed under the reaction conditions [54].

### Photoreduction of 2-benzoylimidazolium triflate with triethylsilane

**Scheme 3:**
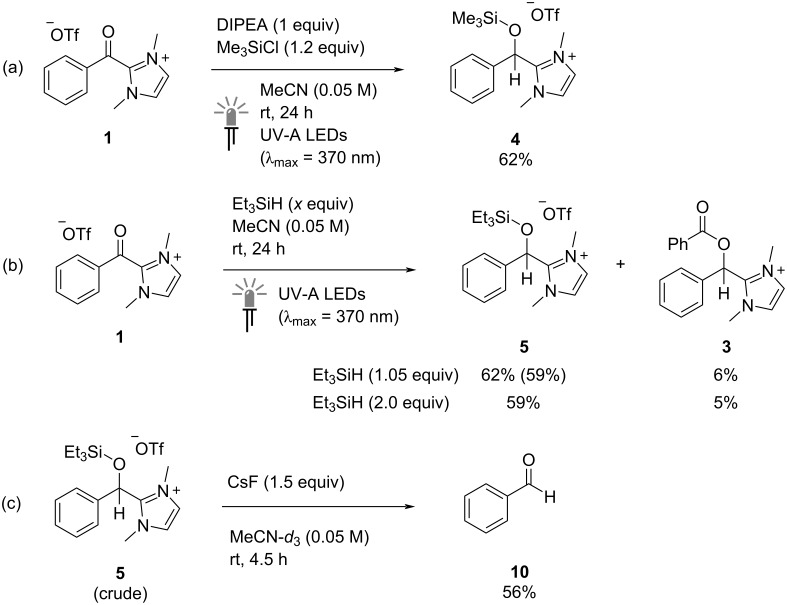
(a) Reduction of 2-benzoylimidazolium triflate (**1**) under photocatalyst-free conditions with DIPEA and TMSCl. (b) Reduction of 2-benzoylimidazolium triflate (**1**) under photocatalyst-free conditions with TESH. (c) Desilylation and NHC elimination of TES-protected alkoxide **5** with CsF. ^1^H NMR yields reported using CH_2_Br_2_ as an internal standard, isolated yields in parentheses.

Although the DIPEA-mediated photochemical reduction of benzoylimidazolium species **1** was successful, the reaction stoichiometry where two molecules of the starting material provide only one molecule of the reduced products **2** or **3** limits the overall efficiency. To overcome this limitation, we considered whether a different approach could provide reduced products in yields greater than 50% relative to the starting material **1**. In particular, addition of chloro(trimethyl)silane (TMSCl) to the reaction mixture could provide an electrophile capable of trapping the putative alkoxide zwitterionic intermediate **E**, outcompeting *O*-benzoylation by a second molecule of **1**. Pleasingly, conducting the standard photocatalyst-free reduction of **1** with 1 equivalent of DIPEA under UV-A light irradiation (λ_max_ = 370 nm) in the presence of 1.2 equivalents of TMSCl resulted in the formation of new peaks in the crude ^1^H NMR spectrum consistent with the desired silyl-protected species **4** (Scheme 3a). Crucially, the calculated ^1^H NMR yield of this species (62%) is higher than the maximum of 50% possible from the mechanism discussed above.

A further intriguing result was obtained upon switching from a chlorosilane to a hydrosilane. To our surprise, irradiating benzoylimidazolium species **1** with UV-A light (λ_max_ = 370 nm) in MeCN (0.05 M) in the presence Et_3_SiH (TESH, 1.2 equiv) led to smooth conversion into the silylated species **5** (^1^H NMR yield = 62%; with 6% of **3**) even in the absence of DIPEA (Scheme 3b). This remarkable result shows that simple silanes are capable of reducing NHC-derived acylazolium species with the calculated ^1^H NMR yield above 50% indicating that this approach is potentially higher yielding than the DIPEA-mediated reduction developed above. Performing the same reaction with a selection of electronically diverse derivatives of **1** bearing 4-CF_3_ (**6**), 4-CO_2_Me (**7**) and 4-OMe (**8**) groups also resulted in the formation of reduced products with ^1^H NMR spectra consistent with *O*-silylated species (^1^H NMR yields: from **6** = 54%; from **7** = 53%, from **8** = 38%), however, a representative aliphatic species, 2-(cyclohexanoyl)-1,3-(dimethyl)imidazolium triflate (**9**) was unreactive. The lower efficiency of the reaction with the 4-methoxy-substituted azolium **8** (30% starting material remaining) is consistent with the expected higher reduction potential of this comparatively electron-rich compound. Purification of the crude reaction mixture from **1** by column chromatography allowed for the isolation of pure product **5** as a colorless oil in 59% yield. A control reaction performed under the same conditions in the dark resulted only in recovered starting material, while **5** was formed in only 5% ^1^H NMR yield when **1** and TESH (1.2 equiv) were irradiated with blue light (λ_max_ = 440 nm) in the presence of the photocatalyst 4CzIPN (2 mol %). These results imply that the silane-mediated reduction is light-mediated and likely involves direct excitation of the benzoylazolium salt with the excited state species 1*, which subsequently reacts with TESH. Comparison of the redox potential of the silane (+1.54 V vs ferrocene) with the estimated excited-state potential of the azolium salt 1* (+1.70 V vs ferrocene) suggest that direct electron transfer between these species affording zwitterionic radical **E** is thermodynamically feasible (for details, see Supporting Information File 1). In an alternative pathway, direct hydrogen atom transfer (HAT) from the silane to the oxygen atom of excited 1* could occur. A similar HAT-step from 1* was very recently proposed by Marzo and co-workers in an NHC-mediated coupling reaction with alkyl halides mediated by (TMS)_3_SiH [55]. The *O*-silylated product **5** could result from a subsequent radical *C*-silylation followed by a Brook-type rearrangement process [56]. Interestingly, performing the TESH-mediated reduction in the presence of additional TMSCl led to the formation of both possible *O*-silylated products with the TMS-substituted compound **4** being the major species obtained (^1^H NMR yields: **4** = 34%; **5** = 12%). This intriguing result indicates that the *O*-silylation step is to some degree decoupled from the reduction process. Further mechanistic studies will be required, however, to fully elucidate the active pathways occurring in this system.

Finally, treatment of crude **5** with CsF (1.5 equiv) in MeCN-*d*_3_ at rt led to efficient desilylation and elimination of the NHC, affording benzaldehyde (**10**) in 56% ^1^H NMR yield (Scheme 3c). The successful generation of the aldehyde from the azolium species derived from the corresponding carboxylic acid highlights the potential of this two-step sequence as a method for the partial reduction of carboxyl compounds. Such transformations can be challenging in organic synthesis with complete reduction to the alcohol followed by partial re-oxidation often being conducted.

## Conclusion

In conclusion, we have developed novel light-driven methodologies for the reduction of acylazolium salts using benzoylimidazolium triflate as a model substrate. In the presence of a photocatalyst under blue light irradiation, the simple tertiary amine DIPEA can serve as the terminal reductant, delivering predominantly the fully reduced benzylimidazolium species as the major product. Irradiation with UV-A light in the absence of a photocatalyst, however, also provides reduced products with an *O*-benzoylated intermediate species also being obtained under some conditions. Supported by TD-DFT studies, plausible reaction mechanisms for both processes were proposed. Moreover, further investigations employing silicon-containing additives allowed for an increase in the reduction yield beyond the maximum of 50% achievable using DIPEA. This study also led to the development of a novel silane-mediated photoreduction method where triethylsilane serves as both as terminal reductant and alkoxide trapping reagent, with subsequent treatment with fluoride providing the corresponding aldehyde. Given the importance of carboxyl reduction reactions in organic synthesis and the recent explosion in interest in light-mediated NHC-catalyzed coupling reactions, we believe this work will be of value to the community and further studies on these systems, including on the development of catalytic applications, are underway in our laboratory.

## Supporting Information

File 1 Experimental procedures, characterization data of all isolated products, details of UV–vis, time-course and computational studies as well as copies of NMR spectra for novel compounds.

## Data Availability

Data generated and analyzed during this study is available from the corresponding author upon reasonable request.
