# Supplementary material for: Photochemical reduction of acylimidazolium salts
Source: Beilstein J Org Chem. 2025 Sep 25;21:1973–83. doi: 10.3762/bjoc.21.153 (PMC12477902; doi:10.3762/bjoc.21.153)
Supplement: File 1 — Experimental procedures, characterization data of all isolated products, details of UV–vis, time-course and computational studies as well as copies of NMR spectra for novel compounds. [file Beilstein_J_Org_Chem-21-1973-s001.pdf]

BEILSTEIN JOURNAL OF ORGANIC CHEMISTRY

Supporting Information

for

Photochemical reduction of acylimidazolium salts

Michael Jakob, Nick Bechler, Hassan Abdelwahab, Fabian Weber, Janos Wasternack, Leonardo Kleebauer, Jan P. Götze and Matthew N. Hopkinson

Beilstein J. Org. Chem. **2025,** *21,* 1973–1983. doi:10.3762/bjoc.21.153

Experimental procedures, characterization data of all isolated products, details of UV–vis, time-course and computational studies as well as copies of NMR spectra for novel compounds

License and Terms: This is a supporting information file under the terms of the Creative Commons Attribution License (https://creativecommons.org/licenses/by/4.0). Please note that the reuse, redistribution and reproduction in particular requires that the author(s) and source are credited and that individual graphics may be subject to special legal provisions.
The license is subject to the *Beilstein Journal of Organic Chemistry* terms and conditions: (https://www.beilstein-journals.org/bjoc/terms)

Table of contents

1 General information S2

2 Preparations S4

2.1 Preparation of imidazole precursors S5

2.2 Preparation of 1,3-dimethyl-1*H*-imidazol-3-ium compounds S10

3 Photoreduction of acyl azolium 1 using DIPEA as reductant S14

3.1 NMR yield determination S15

3.2 Screening of reaction conditions S15

3.3 Mechanistic experiments S18

4 Photoreduction of acyl azolium 1 using triethylsilane as reductant S26

4.1 Screening of reaction conditions & isolation of silyl ether **5** S26

4.2 Further experiments S28

5 Computational section S31

5.1 Conformer search with CREST S31

5.2 Quantum chemical calculations with Gaussian S31

5.3 NTO calculations with ORBKIT S33

5.4 Structures S33

6 References S41

7 NMR spectra of novel compounds S43

1 General information

All air- and moisture-sensitive procedures were performed under inert conditions (dry solvents, protective nitrogen atmosphere, heat-gun-dried glassware, standard Schlenk techniques). Anhydrous solvents were purchased (Acros Organics). The technical grade solvents were distilled under reduced pressure prior to use. The photocatalyst 4CzIPN was prepared for an earlier project in our laboratories.[1] All other reagents and starting materials were commercially obtained and used without further purification, if no preparation is described or if not otherwise stated below.

Chromatography: Thin-layer chromatography (TLC) was performed on silica-coated aluminum plates produced by Macherey-Nagel GmbH & Co. KG (ALUGRAM® Xtra SIL G/UV_{254}). Spots were visualized by UV light (λ = 254nm) or by permanganate staining. Flash column chromatographic separation was carried out manually with silica 60 M (230–400 mesh, 0.04–0.063 mm) produced by Macherey-Nagel GmbH & Co. KG as stationary phase.

NMR spectra were recorded at ambient temperature on the instruments Bruker AVANCE 300 III, JEOL ECX400, Bruker AVANCE 500 III, JEOL ECZ600 and Bruker AVANCE 700. Chemical shifts (δ) are expressed in parts per million (ppm) relative to tetramethylsilane. Proton and carbon spectra are referenced to the residual signal of the respective solvents chloroform (^{1}H: δ = 7.26 ppm; ^{13}C: δ = 77.16 ppm) or acetonitrile (^{1}H: δ = 1.94 ppm; ^{13}C: 118.26 ppm or 1.32 ppm). Fluorine spectra are not calibrated. Coupling constants (*J*) are reported in hertz (Hz) and refer to the H–H coupling if not otherwise stated. Multiplicities are denoted as follows: s = singlet, d = doublet, t = triplet, q = quartet, m = multiplet, br = broad. All ^{13}C NMR spectra were recorded ^{1}H decoupled. NMR yields of crude mixtures were determined by the addition of dibromomethane as internal standard.

High-resolution mass spectra (**HRMS**) were measured with an Agilent 6210 ESI-TOF (4 µL/min, 1.0 bar, 4 kV) instrument. **IR absorption spectra** were recorded with a Bruker Alpha II FT-IR spectrometer. The wavelengths are given in $\tilde{\nu}$ [cm^{-1}]. Only diagnostic absorption bands are reported. The intensities are described as vs = very strong, s = strong, m = medium, w = weak and br = broad. **UV/Vis spectra** were measured with a Varian Cary 50 Bio UV-Visible Spectrophotometer (scan rate = 4800.00 nm/min, data interval = 1.00 nm).

Photoreactions were performed as shown in Figure S1 using LED chips (4 × 4 cm^2, 33 V, 0.7 A, 23 W) by the brand SZRZGT attached to either a Voltcraft LSP-1165 or LSP-1403 power supply. The reaction mixture was irradiated in a distance of 2 cm. Emission spectra of the two used LED chips (UV-A and blue light) are shown in Figure S2.

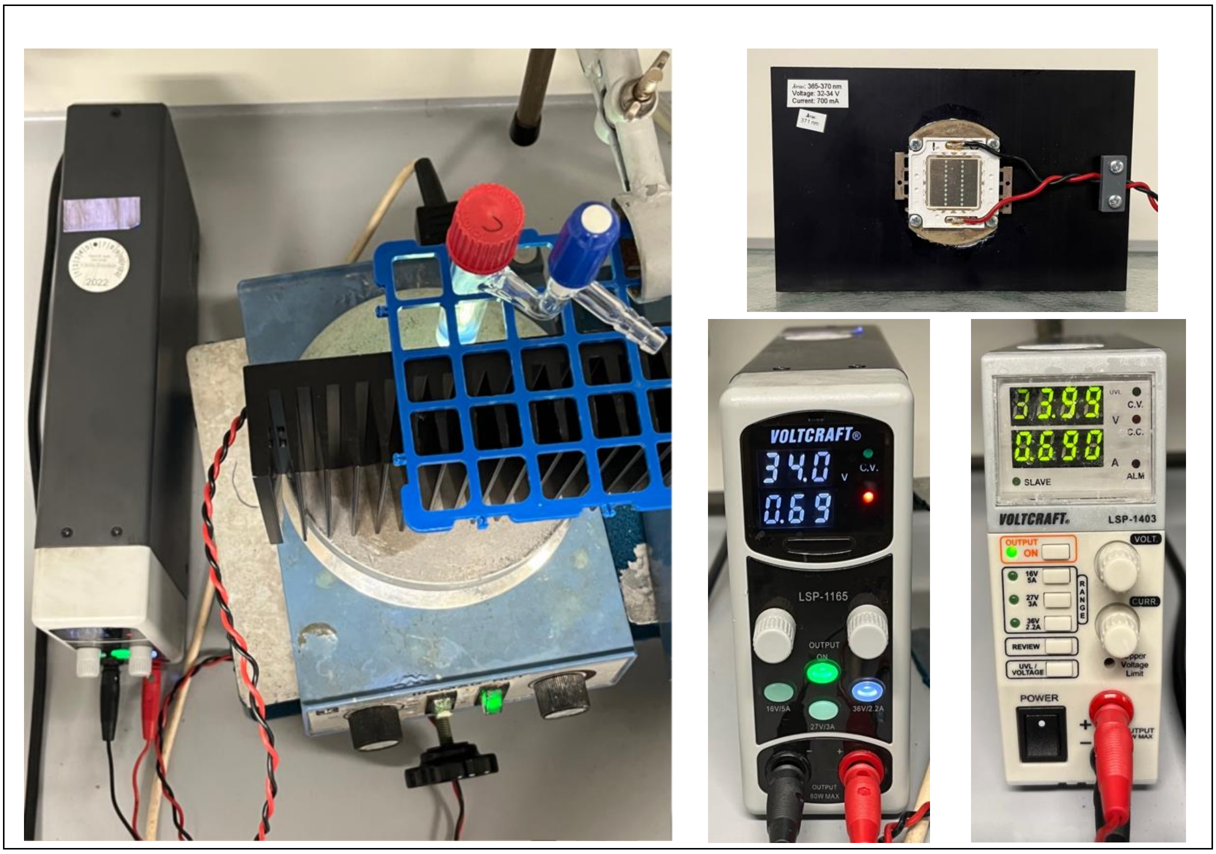

Figure S1: Photoreaction set-up (left). LED chip construction, powered by either a Voltcraft LSP-1165 or an LSP-1403 power supply (right).

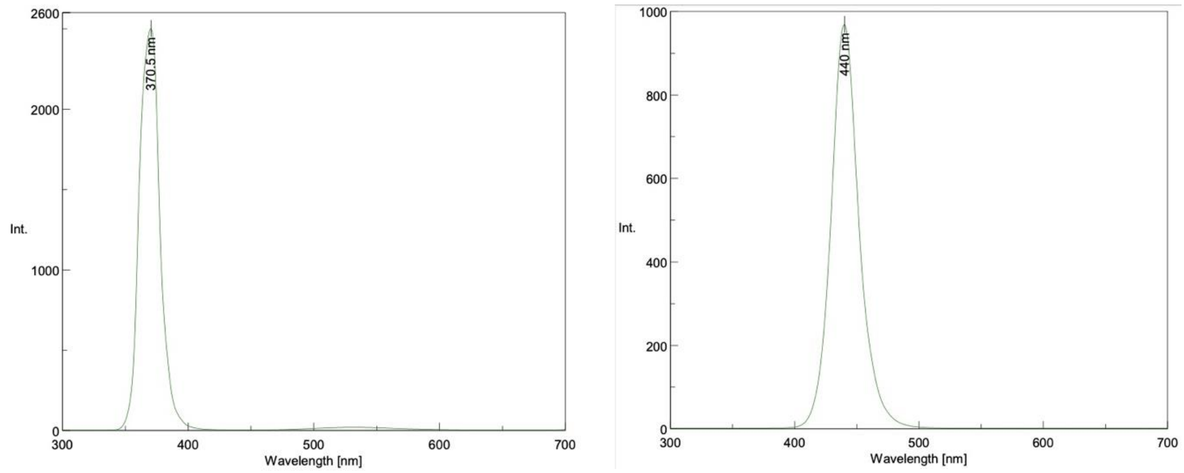

Figure S2: Emission spectra of LED chips measured with a JASCO FP-8200 fluorescence spectrometer; UV-A (left), blue light (right).

2 Preparations

An overview of the preparations of acyl azolium **1** and independently prepared photoreduction products **2**, **3**, and **S1** is shown in Figure S3.

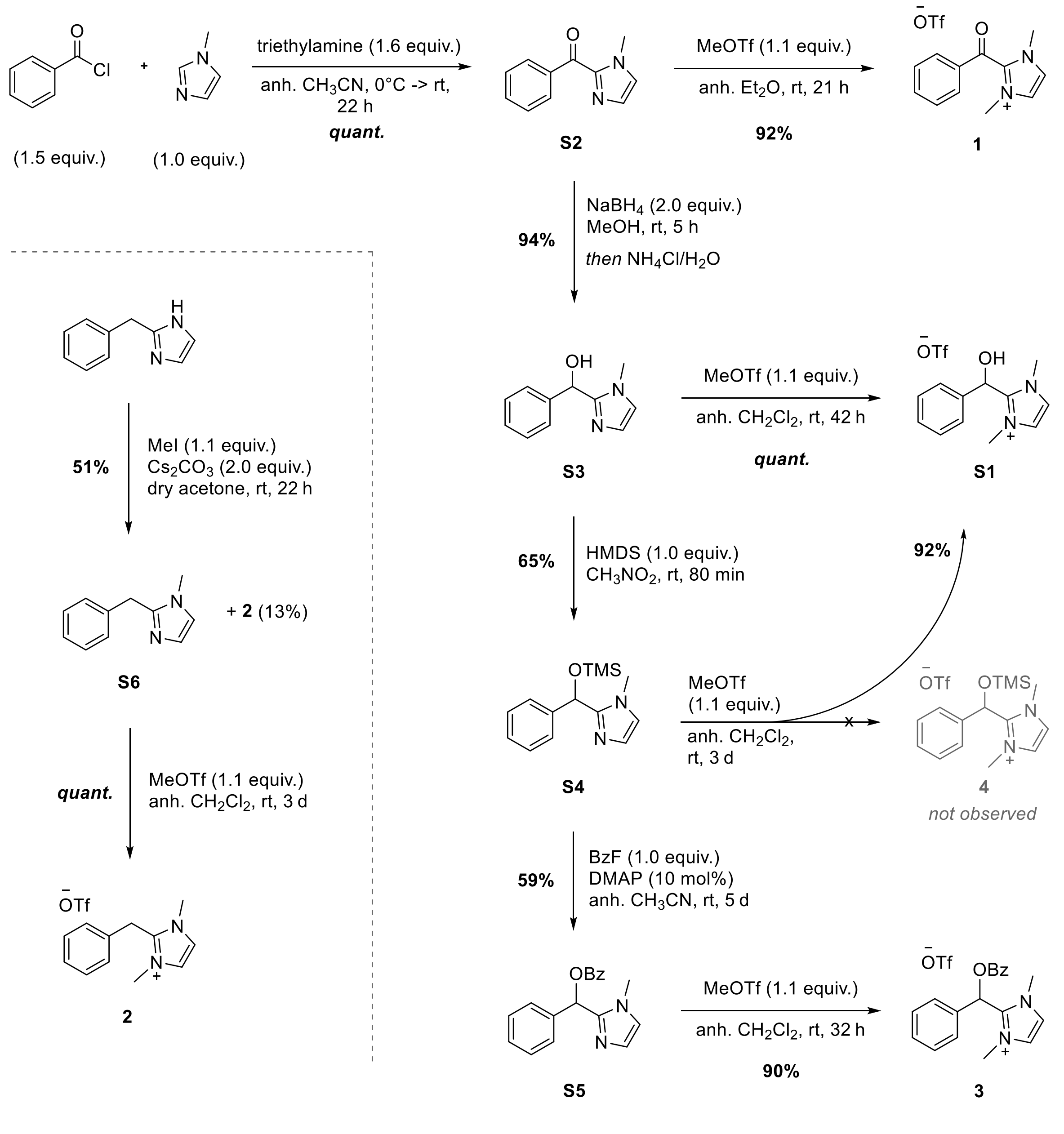

Figure S3: Overview of the preparations of the azolium salts **1**, **2**, **3**, and **S1**; MeOTf = methyl triflate; HMDS = hexamethyldisilazane; DMAP = 4-dimethylaminopyridine; TMS = trimethylsilyl; Bz = benzoyl.

2.1 Preparation of imidazole precursors

General procedure A:

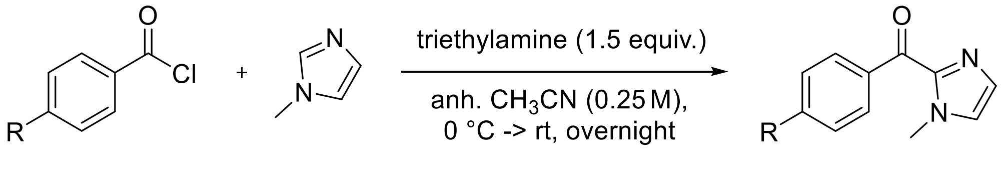

1-Methylimidazole (797 µL, 821 mg, 10.0 mmol, 1.0 equiv) was dissolved in anh. CH_3CN (40 mL, 0.25 M) and the solution was cooled in an ice bath. Benzoyl chloride (15.0 mmol, 1.5 equiv) and dropwise dry triethylamine (2.10 mL, 1.52 g, 15.0 mmol, 1.5 equiv) were added. The reaction mixture was allowed to stir overnight while slowly warming to room temperature. H_2O (20 mL) and EtOAc (25 mL) were added, and the mixture was transferred into a separation funnel. The aq. layer was separated and extracted with EtOAc (2 × 20 mL). The combined org. layer was washed with brine (1 × 15 mL), dried over $MgSO_4$, filtered and the solvent was removed under reduced pressure. The resulting crude product was purified by column chromatography.

(1-Methyl-1*H*-imidazol-2-yl)(phenyl)methanone (S2)

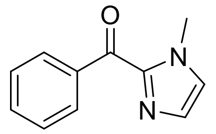

S2

According to *general procedure A*, 1-methylimidazole (800 µL, 824 mg, 10.0 mmol, 1.0 equiv) and benzoyl chloride (1.77 mL, 2.14 g, 15.2 mmol, 1.5 equiv) were reacted for 22 h. Purification via column chromatography (silica gel, *n*-hexane/ethyl acetate 2:1) afforded methanone **S2** as a yellow oil (1.87 g, 10.0 mmol, *quant.*). **R_f:** 0.21 (*n*-hexane/EtOAc 2:1); **^{1}H NMR** (400 MHz, $CDCl_3$): δ = 8.26–8.24 (m, 2H), 7.59–7.55 (m, 1H), 7.51–7.46 (m, 2H), 7.24 (d, *J* = 0.9 Hz, 1H), 7.11–7.11 (m, 1H), 4.08 (s, 3H) ppm.
Analytical data was found to be in agreement with the literature.[1,2]

(1-Methyl-1*H*-imidazol-2-yl)(phenyl)methanol (S3)

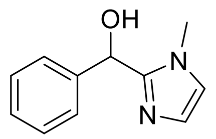

S3

Following a known procedure,[3] methanone **S2** (1.31 g, 7.03 mmol, 1.0 equiv) was dissolved in anh. MeOH (30 mL) and sodium borohydride (532 mg, 14.1 mmol, 2.0 equiv) was added slowly to the stirred solution at room temperature. After stirring for 4 d, the solution was cooled in an ice bath and sat. aq. NH_4Cl solution (ca. 20 mL) was added, and the mixture was allowed to stir for 5 min. The mixture was diluted with H_2O (10 mL), transferred into a separation funnel (eluent: EtOAc) and the aq. layer was extracted with EtOAc (3 × 20 mL). The combined org. layer was washed with brine (1 × 10 mL), dried over $MgSO_4$, filtered and the solvent was removed under reduced pressure. Purification via column

chromatography (silica gel, ethyl acetate/methanol 1:0 → 1:10) afforded alcohol **S3** as a yellowish oil (1.24 g, 6.59 mmol, 94%).
R*f*: 0.30 (EtOAc, tailing); **^{1}H NMR** (400 MHz, $CDCl_3$): δ = 7.35–7.28 (m, 4H), 7.28–7.22 (m, 1H), 6.85 (d, *J* = 1.0 Hz, 1H), 6.72 (s, 1H), 5.95 (s, 1H), 3.38 (s, 3H) ppm. Analytical data was found to be in agreement with the literature.[3]

(1-Methyl-2-(phenyl(trimethylsilyl)oxy)methyl-1*H*-imidazole (S4)

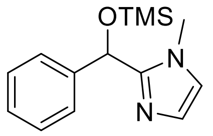

S4

According to a known procedure,[4] alcohol **S3** (790 mg, 4.20 mmol, 1.0 equiv) was dissolved in nitromethane (4.2 mL, 1 M) and hexamethyldisilazane (0.90 mL, 0.70 g, 4.3 mmol, 1.0 equiv) was dropped to the solution. After 80 min of stirring at room temperature, the solvent was removed under reduced pressure and the crude product was dried in vacuo. Purification via column chromatography (silica gel, ethyl acetate) afforded silyl ether **S4** as a colorless to slightly yellowish oil (707 mg, 2.71 mmol, 64%).
R*f*: 0.57 (EtOAc); **^{1}H NMR** (500 MHz, $CDCl_3$): δ = 7.36–7.28 (m, 4H), 7.25–7.20 (m, 1H), 6.93 (d, *J* = 1.2 Hz, 1H), 6.74 (d, *J* = 1.2 Hz, 1H), 6.12 (s, 1H), 3.36 (s, 3H), 0.09 (s, 9H) ppm; **^{13}C NMR** (151 MHz, $CDCl_3$): δ = 148.21, 141.20, 128.30, 127.25, 126.88, 125.50, 122.33, 71.04, 33.28, −0.33 ppm; **HRMS-ESI:** *m/z* calculated for $[C_{14}H_{21}N_2OSi]^+$ ($[M+H]^+$): 261.1418, found: 261.1429; **IR (ATR):** $\tilde{\nu}$ = 1254 (m, δ_s(Si–C–H)), 842 (vs, δ_{as}(Si–C–H)) cm^{-1}.
^{1}H NMR spectral data is comparable with reported data, that was measured in CCl_4.[5]

[*NB: After only 60 min reaction time, another batch on a 4.08 mmol scale gave silyl ether* ***S4*** *with a reduced yield of 50%.*]

(1-Methyl-1*H*-imidazol-2-yl)(phenyl)methyl benzoate (S5)

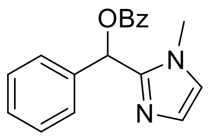

S5

According to a known procedure,[6] silyl ether **S4** (384 mg, 1.47 mmol, 1.0 equiv) was dissolved in anh. CH_3CN (3.0 mL, 0.5 M). Benzoyl fluoride (160 µL, 183 mg, 1.47 mmol, 1.0 equiv) and DMAP (18 mg, 0.15 mmol, 10 mol %) were added and the mixture was allowed to stir for 5 d at room temperature. The mixture was transferred into a one-necked flask (eluent: CH_2Cl_2) and the solvent was removed under reduced pressure. Purification via column chromatography (silica gel, ethyl acetate) afforded benzoate **S5** as a colorless solid (252 mg, 0.862 mmol, 59%).
R*f*: 0.54 (EtOAc); **mp:** 150.0–150.2 °C (turns orange while heating, melts as brown oil); **^{1}H NMR** (500 MHz, CDCl3): δ = 8.13 (d, *J* = 7.8 Hz, 2H), 7.57 (t, *J* = 7.2 Hz, 1H), 7.49–743 (m, 4H), 7.38 (t, *J* = 7.4 Hz, 2H), 7.36–7.30 (m, 1H), 7.29 (s, 1H), 7.06 (s, 1H), 6.88 (s, 1H), 3.67 (s, 3H) ppm; **^{13}C NMR** (126 MHz, $CDCl_3$): δ = 165.49, 144.97, 136.82, 133.55, 130.03, 129.50, 128.80, 128.61, 128.59, 127.83, 126.98, 122.30, 70.17, 33.42 ppm; **HRMS-ESI:** *m/z* calculated for $[C_{18}H_{17}N_2O_2]^+$ ($[M+H]^+$): 293.1285, found: 293.1296; **IR (ATR):** $\tilde{\nu}$ = 1710 (s, *ν*(C=O)) cm^{-1}.

Alternative "one-pot" approach for the preparation of S5:

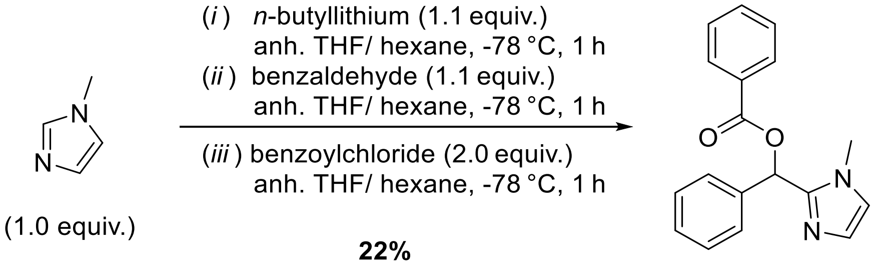

S5

1-Methylimidazole (0.40 mL, 0.41 g, 5.0 mmol, 1.0 equiv) was dissolved in anh. THF (25 mL) and the solution was cooled to −78 °C. At this temperature, *n*-butyllithium (1.6 M in hexane, 3.44 mL, 5.5 mmol, 1.1 equiv) was added slowly and the mixture was allowed to stir for 1 h. Benzaldehyde (0.56 mL, 0.59 mg, 5.5 mmol, 1.1 equiv) was dropped slowly to the −78 °C cold solution which turned red after addition. The mixture was allowed to stir for an hour at this temperature, while its color turns light brown and lastly yellow over time. Benzoyl chloride (1.15 mL, 1.40 g, 10.0 mmol, 2.0 equiv) was added to the −78 °C cold solution and was allowed to stir for 1 h at this temperature. The mixture was allowed to warm to room temperature and H_2O (25 mL) was added. The mixture was transferred into a separation funnel and the aq. layer was extracted with EtOAc (3 × 20 mL). The combined org. layer was dried over Na_2SO_4, filtered and the solvent was removed under reduced pressure. Purification via column chromatography (silica gel, *n*-pentane/ethyl acetate 4:1 → 0:1) afforded benzoate **S5** as a colorless solid (320 mg, 1.09 mmol, 22%).

2-Benzyl-1-methyl-1*H*-imidazole (S6)

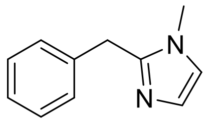

S6

According to a known procedure,[7] 2-benzyl-1*H*-imidazole (400 mg, 2.53 mmol, 1.0 equiv) was dissolved in dry acetone (13 mL, 0.2 M) and Cs_2CO_3 (1.65 g, 5.06 mmol, 2.0 equiv) was added. Iodomethane (175 µL, 399 mg, 2.81 mmol, 1.1 equiv) was dropped to the yellow suspension and the mixture was allowed to stir at room temperature for 22 h. The mixture was filtered through a short pad of celite (eluent: acetone) and the solvent was removed under reduced pressure. Purification via column chromatography (silica gel, dichloromethane/methanol 9:1) afforded imidazole **S6** as a yellow oil (222 mg, 1.29 mmol, 51%).

R_f: 0.51 (DCM/MeOH 9:1); **^{1}H NMR** (500 MHz, CDCl3): δ = 7.28 (t, J = 7.4 Hz, 2H), 7.21 (t, J = 7.4 Hz, 1H), 7.18–7.15 (m, 2H), 6.97 (d, J = 1.0 Hz, 1H), 6.80 (d, J = 1.0 Hz, 1H), 4.12 (s, 2H), 3.44 (s, 3H) ppm; **IR (ATR):** $\tilde{\nu}$ = 3113 (w, $\nu(C–H)_{arom}$), 3033 (w, $\nu(C–H)_{arom}$), 2953 (w, $\nu(C–H)_{aliph}$) cm^{-1}.

Analytical data was found to be in agreement with the literature.[7]

[NB: *After column chromatography, also the double methylated target molecule **2** was isolated (113.2 mg, 0.336 mmol, 13% + mixed fraction); **R_f**: 0.27 (DCM/MeOH 9:1, tailing).*]

(1-Methyl-1*H*-imidazol-2-yl)(4-(trifluoromethyl)phenyl)methanone (S7)

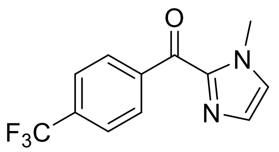

S7

According to *general procedure A*, 1-methylimidazole (797 µL, 821 mg, 10.0 mmol, 1.0 equiv) and 4-(trifluoromethyl)benzoyl chloride (2.23 mL, 3.13 g, 15.0 mmol, 1.5 equiv) were reacted overnight. Purification via column chromatography (silica gel, petroleum ether/diethyl ether 2:1) afforded methanone **S7** as an orange solid (2.00 g, 7.87 mmol, 79%).

R_f: 0.33 (petroleum ether/Et_2O 2:1); **^{1}H NMR** (400 MHz, $CDCl_3$): δ = 8.36 (d, J = 8.0 Hz, 2H), 7.74 (d, J = 8.2 Hz, 2H), 7.25 (s, 1H), 7.16 (s, 1H), 4.10 (s, 3H); **^{19}F NMR** (376 MHz, $CDCl_3$): δ = −63.1 ppm; **^{13}C NMR** (101 MHz, $CDCl_3$): δ = 183.1, 142.8, 140.4, 133.9 (q, J = 33 Hz), 131.1, 129.9, 127.6, 125.2 (q, J = 4 Hz), 122.5, 36.7 ppm. Analytical data was found to be in agreement with the literature.[8,9]

Methyl 4-(1-methyl-1*H*-imidazole-2-carbonyl)benzoate (S8)

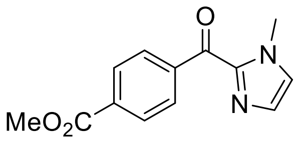

S8

According to *general procedure A*, 1-methylimidazole (797 µL, 821 mg, 10.0 mmol, 1.0 equiv) and methyl 4-(chlorocarbonyl)benzoate (1.99 g, 10.0 mmol, 1.0 equiv) were reacted overnight. Purification via column chromatography (silica gel, petroleum ether/diethyl ether 1:1) afforded methanone **S8** as a grey solid (1.30 g, 5.32 mmol, 53%).

R_f: 0.33 (petroleum ether/Et2O 1:1); **^{1}H NMR** (400 MHz, $CDCl_3$): δ = 8.31 (d, J = 8.5 Hz, 2H), 8.14 (d, J = 8.5 Hz, 2H), 7.25 (s, 1H), 7.14 (s, 1H), 4.10 (s, 3H), 3.95 (s, 3H); **^{13}C NMR** (101 MHz, $CDCl_3$): δ = 183.4, 166.5, 142.8, 140.9, 133.3, 130.7, 129.6, 129.3, 127.3, 52.4, 36.6 ppm.

Analytical data was found to be in agreement with the literature.[8]

(4-Methoxyphenyl)(1-methyl-1*H*-imidazol-2-yl)methanone (S9)

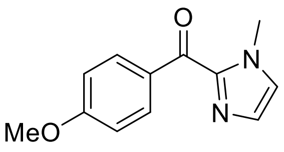

S9

According to *general procedure A*, 1-methylimidazole (797 µL, 821 mg, 10.0 mmol, 1.0 equiv) and 4-methoxybenzoyl chloride (2.03 mL, 2.56 g, 15.0 mmol, 1.5 equiv) were reacted for 13 h. Purification via column chromatography (silica gel, *n*-hexane/ethyl acetate 1:1) afforded methanone **S9** as an off-white solid (1.75 g, 8.09 mmol, 81%).

R_f: 0.32 (*n*-hexane/EtOAc 1:1); **^{1}H NMR** (500 MHz, $CDCl_3$): δ = 8.32 (d, J = 8.9 Hz, 2H), 7.22 (s, 1H), 7.09 (s, 1H), 6.99–6.95 (m, 2H), 4.05 (s, 3H), 3.87 (s, 3H) ppm.

Analytical data was found to be in agreement with the literature.[8]

N-Methoxy-N-methylcyclobutanecarboxamide (S10)

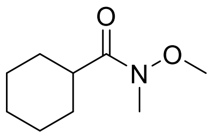

S10

Following a known procedure,[10] cyclohexanecarboxylic acid (1.28 g, 10.0 mmol, 1.0 equiv) was dissolved in anh. CH_2Cl_2 (25 mL) and *N,N'*-carbonyldiimidazole (2.11 g, 13.0 mmol, 1.3 equiv) was added slowly to the stirred solution at room temperature. After stirring for 1 h, *N,O*-dimethylhydroxylamine hydrochloride (1.95 g, 20.0 mmol, 2.0 equiv) was added and the mixture was allowed to stir overnight (16 h) at room temperature. Aq. HCl (1 M, 100 mL) was added, and the mixture was allowed to stir for further 15 min. The mixture was transferred into a separation funnel (eluent: CH_2Cl_2) and was extracted (CH_2Cl_2, 3 × 25 mL). The combined org. layer was washed with aq. sat. $NaHCO_3$ (1 × 100 mL), then brine (1 × 100 mL), dried over $MgSO_4$, filtered, and the solvent was removed under reduced pressure. Purification via column chromatography (silica gel, *n*-hexane/ethyl acetate 2:1) afforded Weinreb amide **S10** as a colorless oil (1.34 g, 7.82 mmol, 78%).

R*f*: 0.29 (*n*-hexane/EtOAc 2:1); **^{1}H NMR** (700 MHz, $CDCl_3$): δ = 3.68 (s, 3H), 3.16 (s, 3H), 2.67 (br s, 1H), 1.80–1.76 (m, 2H), 1.76–1.72 (m, 2H), 1.68–1.65 (m, 1H), 1.50–1.43 (m, 2H), 1.32–1.20 (m, 3H) ppm; **^{13}C NMR** (176 MHz, $CDCl_3$): δ = 177.6, 61.6, 40.1, 32.3, 29.1, 25.9, 25.9 ppm.

Analytical data was found to be in agreement with the literature.[10]

Cyclohexyl(1-methyl-1H-imidazol-2-yl)methanone (S11)

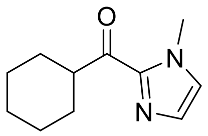

S11

Similar to a known procedure,[11] 1-methylimidazole (0.78 mL, 0.80 g, 9.78 mmol, 1.3 equiv) was dissolved in anh. THF (60 mL) and the solution was cooled to −78 °C. At this temperature, *n*-butyllithium (2.5 M in hexane, 3.91 mL, 9.78 mmol, 1.3 equiv) was added slowly. The mixture was allowed to warm to room temperature, was stirred for 30 min and was cooled again to −78 °C. A solution of Weinreb amide **S10** (1.28 g, 7.50 mmol, 1.0 equiv) in anh. THF (15 mL) was added, and the mixture was allowed to stir overnight (16 h) while slowly warming to room temperature. Glacial acetic acid (3.0 mL) was added, and the mixture was allowed to stir for 15 min. Aq. sat. $NaHCO_3$ (30 mL) was added, the mixture was transferred into a separation funnel (eluent: Et_2O) and was extracted with Et_2O (3 × 30 mL). The combined org. layer was washed with aq. sat. $NaHCO_3$ (1 × 30 mL), then brine (1 × 30 mL), dried over $MgSO_4$, filtered, and the solvent was removed under reduced pressure. Purification via column chromatography (silica gel, *n*-pentane/diethyl ether 2:1) afforded methanone **S11** as a colorless solid (1.35 g, 7.02 mmol, 94%).

R*f*: 0.44 (*n*-pentane/Et2O 1:1); **^{1}H NMR** (600 MHz, $CDCl_3$): δ = 7.12 (d, *J* = 0.9 Hz, 1H), 6.99 (d, *J* = 0.9 Hz, 1H), 3.96 (s, 3H), 3.66–3.60 (m, 1H), 1.93–1.85 (m, 2H), 1.82–1.74 (m, 2H), 1.71–1.66 (m, 1H), 1.45–1.34 (m, 4H), 1.25–1.16 (m, 1H) ppm; **^{13}C NMR** (151 MHz, CDCl3): δ = 196.5, 142.5, 128.9, 127.0, 46.1, 36.4, 29.2, 26.0, 25.7 ppm.

Analytical data was found to be in agreement with the literature.[9]

2.2 Preparation of 1,3-dimethyl-1*H*-imidazol-3-ium compounds

General procedure B:

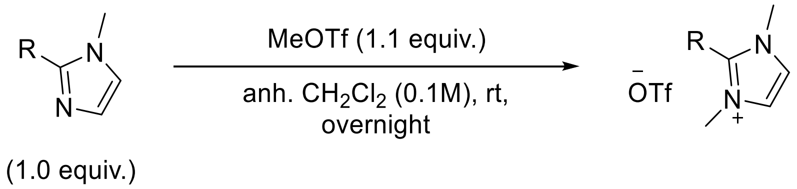

Methyl triflate (1.1 equiv) was dropped to a solution of *N*-methyl-1*H*-imidazole **S3–S9** or **S11** (1.0 equiv) in anh. CH_2Cl_2 (0.1 M) at room temperature and the mixture was allowed to stir overnight under N_2. The solvent was removed by a light stream of N_2 and the resulting product was dried in vacuo. The crude product was washed (3 ×, Et_2O) and dried in vacuo again.

2-Benzoyl-1,3-dimethyl-1*H*-imidazol-3-ium trifluoromethanesulfonate (1)

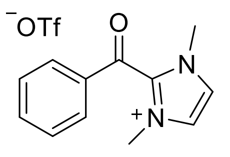

1

Methyl triflate (1.34 mL, 1.94 g, 11.8 mmol, 1.1 equiv) was dropped into a solution of methanone **S2** (1.99 g, 10.7 mmol, 1.0 equiv) in anh. Et_2O (65.0 mL, 0.16 M) at room temperature and the mixture was allowed to stir for 21 h under argon. The colorless precipitate was filtered under reduced pressure, washed with Et_2O (3 × 50 mL) and dried in vacuo, affording acyl azolium **1** as a colorless solid (3.45 g, 9.85 mmol, 92%).
^{1}H NMR (400 MHz, $CDCl_3$): δ = 8.00 (d, *J* = 7.6 Hz, 2H), 7.80 (t, *J* = 7.4 Hz, 1H), 7.79 (s, 2H), 7.66 (t, *J* = 7.7 Hz, 2H), 3.88 (s, 6H) ppm; **^{1}H NMR** (600 MHz, CD_3CN): δ = 7.91–7.88 (m, 2H), 7.86 (tt, *J* = 7.5, 1.3 Hz, 1H), 7.69–7.65 (m, 2H), 7.59 (dd, *J* = 2.4, 1.6 Hz, 2H), 3.76 (s, 6H) ppm; **^{19}F NMR** (565 MHz, CD_3CN): δ = −79.20 ppm; **^{13}C NMR** (151 MHz, CD_3CN): δ = 181.48, 140.12, 137.39, 135.66, 131.11, 130.68, 126.19, 38.12 ppm.
Analytical data is consistent with that reported in the literature.[12,13]

[NB: *This procedure was reported in a previous publication by our group.*[1] *Due to the key role of acyl azolium* **1** *in this project, we decided to show it again.*]

2-Benzyl-1,3-dimethyl-1*H*-imidazol-3-ium trifluoromethanesulfonate (2)

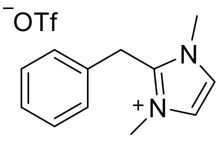

2

According to *general procedure B*, imidazole **S6** (149 mg, 0.865 mmol, 1.0 equiv) and methyl triflate (105 µL, 157 mg, 0.957 mmol, 1.1 equiv) were allowed to stir over the weekend in anh. CH_2Cl_2 (8.7 mL) for 3 d, affording benzyl imidazolium **2** as a slightly yellowish solid (291 mg, 0.865 mmol, *quant.*).
mp: 75.1–77.6 °C; **^{1}H NMR** (600 MHz, $CDCl_3$): δ = 7.40 (s, 2H), 7.33 (t, *J* = 7.3 Hz, 2H), 7.30–7.27 (m, 1H), 7.00 (d, *J* = 7.4 Hz, 2H), 4.42 (s, 2H), 3.78 (s, 6H) ppm; **^{19}F NMR** (376 MHz, $CDCl_3$): δ = −78.38 ppm; **^{13}C NMR** (151 MHz, $CDCl_3$): δ = 145.61, 131.85, 129.78, 128.10, 127.73, 123.11, 35.67, 29.33 ppm; **HRMS-ESI:** *m/z* calculated for $[C_{12}H_{15}N_2]^+$ ($[M]^+$): 187.1230, found: 187.1232; **IR (ATR):** $\tilde{\nu}$ = 3144 (w, ν(C–H)$_{arom}$), 2953 (w, ν(C–H)$_{aliph}$), 1257 (vs, $\nu_{as}(SO_3)$), 1226 (s, $\nu_s(CF_3)$), 1031 (vs, $\nu_s(SO_3)$), 637 (vs, $\delta_s(SO_3)$) cm^{-1}.

2-((Benzoyloxy)(phenyl)methyl)-1,3-dimethyl-1*H*-imidazol-3-ium trifluoromethane-sulfonate (3)

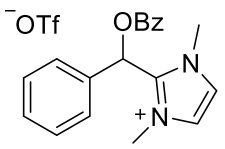

3

According to *general procedure B*, benzoate **S5** (83.6 mg, 0.286 mmol, 1.0 equiv) and methyl triflate (34.4 µL, 51.5 mg, 0.314 mmol, 1.1 equiv) were allowed to stir in anh. CH_2Cl_2 (2.9 mL) for 32 h, affording *O*-benzoylated species **3** as a yellowish oil (118 mg, 0.259 mmol, 90%).

^{1}H NMR (500 MHz, CD_3CN): δ = 8.21–8.17 (m, 2H), 7.76–7.71 (m, 1H), 7.62–7.57 (m, 2H), 7.55–7.49 (m, 3H), 7.49 (s, 1H), 7.44 (s, 2H), 7.42–7.39 (m, 2H), 3.88 (s, 6H) ppm; **^{1}H NMR** (500 MHz, $CDCl_3$): δ = 8.11 (dd, *J* = 8.3, 1.2 Hz, 2H), 7.69 (tt, *J* = 7.2, 1.2 Hz, 1H), 7.66 (s, 2H), 7.58–7.42 (m, 5H), 7.38 (s, 1H), 7.31 (d, *J* = 7.6 Hz, 2H), 3.97 (s, 6H) ppm; **^{19}F NMR** (376 MHz, $CDCl_3$): δ = −78.37 ppm; **^{13}C NMR** (151 MHz, $CDCl_3$): δ = 165.30, 141.54, 134.82, 131.67, 130.10, 130.07, 129.96, 129.19, 127.44, 125.39, 124.91, 65.65, 36.47 ppm; **IR (ATR):** $\tilde{\nu}$ = 1726 (m, ν(C=O)) cm^{-1}.
Analytical data was found to be in agreement with the literature.[9]

2-(Hydroxy(phenyl)methyl)-1,3-dimethyl-1*H*-imidazol-3-ium trifluoromethanesulfonate (S1)

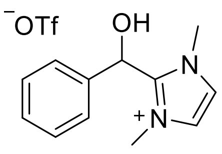

S1

According to *general procedure B*, imidazole **S3** (128.8 mg, 0.684 mmol, 1.0 equiv) and methyl triflate (88 µL, 0.13 g, 0.79 mmol, 1.1 equiv) were allowed to stir in anh. CH_2Cl_2 (6.8 mL) for 42 h, affording the corresponding alcohol **S1** as a colorless oil (233.1 mg, 0.661 mmol, 97%).

^{1}H NMR (600 MHz, $CDCl_3$): δ = 7.41–7.34 (m, 2H), 7.39–7.30 (m, 1H), 7.28 (s, 2H), 7.24–7.19 (m, 2H), 6.39 (s, 1H), 5.52 (br s, 1H), 3.84 (s, 6H) ppm; **^{1}H NMR** (500 MHz, CD_{3CN}): δ = 7.48–7.38 (m, 3H), 7.32 (s, 3H), 7.34–7.29 (m, 2H), 6.40 (s, 1H), 5.22 (br s, 1H), 3.77 (s, 6H) ppm; **^{19}F NMR** (376 MHz, CD_3CN): δ = −79.24 ppm; **^{13}C NMR** (126 MHz, CD_3CN): δ = 146.53, 137.62, 129.98, 129.74, 126.63, 124.48, 65.83, 36.76 ppm; **HRMS-ESI:** *m/z* calculated for $[C_{12}H_{15}N_2O]^+$ ($[M]^+$): 203.1179, found: 203.1187; **IR (ATR):** $\tilde{\nu}$ = 3355 (br, (O–H)), 3147 (w, ν(C–H)$_{arom}$), 1245 (s, ν_{as}(SO_3)), 1226 (s, ν_s(CF_3)), 1030 (vs, ν_s(SO_3)), 639 (s, δ_s(SO_3)) cm^{-1}.

Unexpected desilylation of silyl ether S4 to alcohol S1:

According to *general procedure B*, silyl ether **S4** (125 mg, 0.480 mmol, 1.0 equiv) and methyl triflate (58 µL, 0.09 g, 0.55 mmol, 1.1 equiv) were allowed to stir in anh. CH_2Cl_2 (4.8 mL) for 3 d, affording alcohol **S1** as a colorless oil (154 mg, 0.437 mmol, 92%).

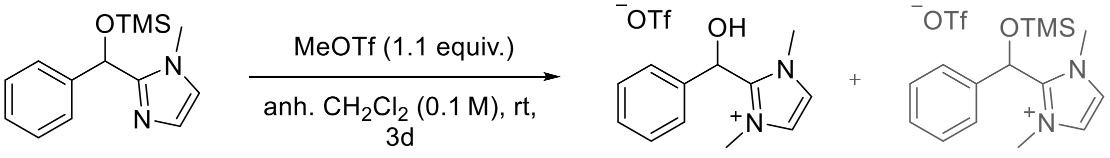

S4 **S1** **4**

92% *(not observed)*

1,3-Dimethyl-2-(4-(trifluoromethyl)benzoyl)-1*H*-imidazol-3-ium trifluoromethane-sulfonate (6)

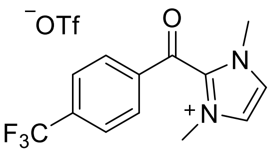

6

Methyl triflate (301 µL, 451 mg, 2.75 mmol, 1.1 equiv) was dropped into a solution of methanone **S7** (635 mg, 2.50 mmol, 1.0 equiv) in anh. Et_2O (25 mL, 0.1 M) at room temperature and the mixture was allowed to stir for 16 h under nitrogen. The colorless precipitate was filtered, washed with Et_2O (3 × 50 mL) and dried in vacuo, affording acyl azolium **6** as a colorless solid (807 mg, 1.93 mmol, 77%).

^{1}H NMR (300 MHz, DMSO-*d*6): δ = 8.17–8.12 (m, 2H), 8.04 (s, 2H), 8.06–8.01 (m, 2H), 3.82 (s, 6H) ppm; **^{19}F NMR** (282 MHz, DMSO-*d*6): δ = −61.91, −77.80 ppm. Analytical data is consistent with that reported in the literature.[8,9]

2-(4-(Methoxycarbonyl)benzoyl)-1,3-dimethyl-1*H*-imidazol-3-ium trifluoromethane-sulfonate (7)

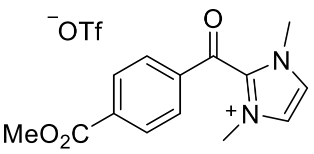

7

Methyl triflate (301 µL, 451 mg, 2.75 mmol, 1.1 equiv) was dropped into a solution of methanone **S8** (610 mg, 2.50 mmol, 1.0 equiv) in anh. Et_2O (25 mL, 0.1 M) at room temperature and the mixture was allowed to stir for 16 h under nitrogen. The colorless precipitate was filtered, washed with Et_2O (3 × 50 mL) and dried in vacuo, affording acyl azolium **7** as a colorless solid (916 mg, 2.24 mmol, 90%).

^{1}H NMR (300 MHz, DMSO-*d*6): δ = 8.22–8.16 (m, 2H), 8.09–8.04 (m, 2H), 8.02 (s, 2H), 3.92 (s, 3H), 3.81 (s, 6H) ppm; **^{13}C NMR** (126 MHz, DMSO-*d*6): δ = 180.00, 165.27, 138.28, 137.88, 135.04, 130.54, 129.86, 125.83, 52.77, 37.69 ppm.
Analytical data is consistent with that reported in the literature.[8]

2-(4-Methoxybenzoyl)-1,3-dimethyl-1*H*-imidazol-3-ium trifluoromethanesulfonate (8)

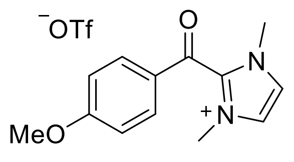

8

Similar to g*eneral procedure B*, methyl triflate (1.00 mL, 1.49 g, 9.10 mmol, 1.1 equiv) was dropped into a solution of methanone **S9** (1.51 g, 7.00 mmol, 1.0 equiv) in anh. CH_2Cl_2 (35 mL, 0.2 M) at room temperature and the mixture was allowed to stir for 48 h under nitrogen. After concentration and precipitation with Et_2O (20 mL), the precipitate was filtered, washed with Et_2O (3 × 50 mL) and dried in vacuo, affording acyl azolium **8** as an off-white solid (2.58 g, 6.79 mmol, 97%).

^{1}H NMR (500 MHz, DMSO-*d*6): δ = 7.96 (s, 2H), 7.93 (d, J = 8.8 Hz, 2H), 7.20 (d, J = 9.0 Hz, 2H), 3.93 (s, 3H), 3.78 (s, 6H) ppm; **^{19}F NMR** (376 MHz, DMSO-*d*6): δ = −77.66 ppm; **^{13}C NMR** (126 MHz, DMSO-*d*6): δ = 178.43, 165.80, 139.13, 133.24, 127.27, 125.00, 120.68 (q, J = 322.4 Hz), 115.14, 56.12, 36.84 ppm.

Analytical data is consistent with that reported in the literature.[8,9]

2-(Cyclohexanecarbonyl)-1,3-dimethyl-1*H*-imidazol-3-ium trifluoromethanesulfonate (9)

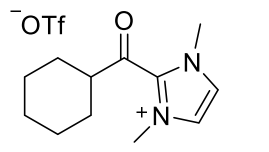

9

Methyl triflate (301 µL, 451 mg, 2.75 mmol, 1.1 equiv) was dropped into a solution of methanone **S11** (960 mg, 5.00 mmol, 1.0 equiv) in anh. Et_2O (50 mL, 0.1 M) at room temperature and the mixture was allowed to stir for 16 h under argon. The colorless precipitate was filtered, washed with Et_2O (3 × 50 mL) and dried in vacuo, affording acyl azolium **9** as a colorless solid (1.63 g, 4.57 mmol, 91%).

^{1}H NMR (500 MHz, DMSO-*d*6): δ = 7.89 (s, 2H), 4.00 (s, 6H), 3.18–3.09 (m, 1H), 1.97–1.88 (m, 2H), 1.78–1.70 (m, 2H), 1.69–1.62 (m, 1H), 1.43–1.29 (m, 4H), 1.26–1.13 (m, 1H) ppm; **^{19}F NMR** (376 MHz, DMSO-*d*6): δ = −77.68 ppm; **^{13}C NMR** (126 MHz, DMSO-*d*6): δ = 191.44, 138.37, 125.41, 120.68 (d, J = 322.5 Hz), 48.59, 37.65, 27.39, 25.17, 24.58 ppm; **IR (ATR):** $\tilde{\nu}$ = 1689 (s, ν(C=O)) cm^{-1}.

Analytical data is consistent with that reported in the literature.[9]

3 Photoreduction of acyl azolium 1 using DIPEA as reductant

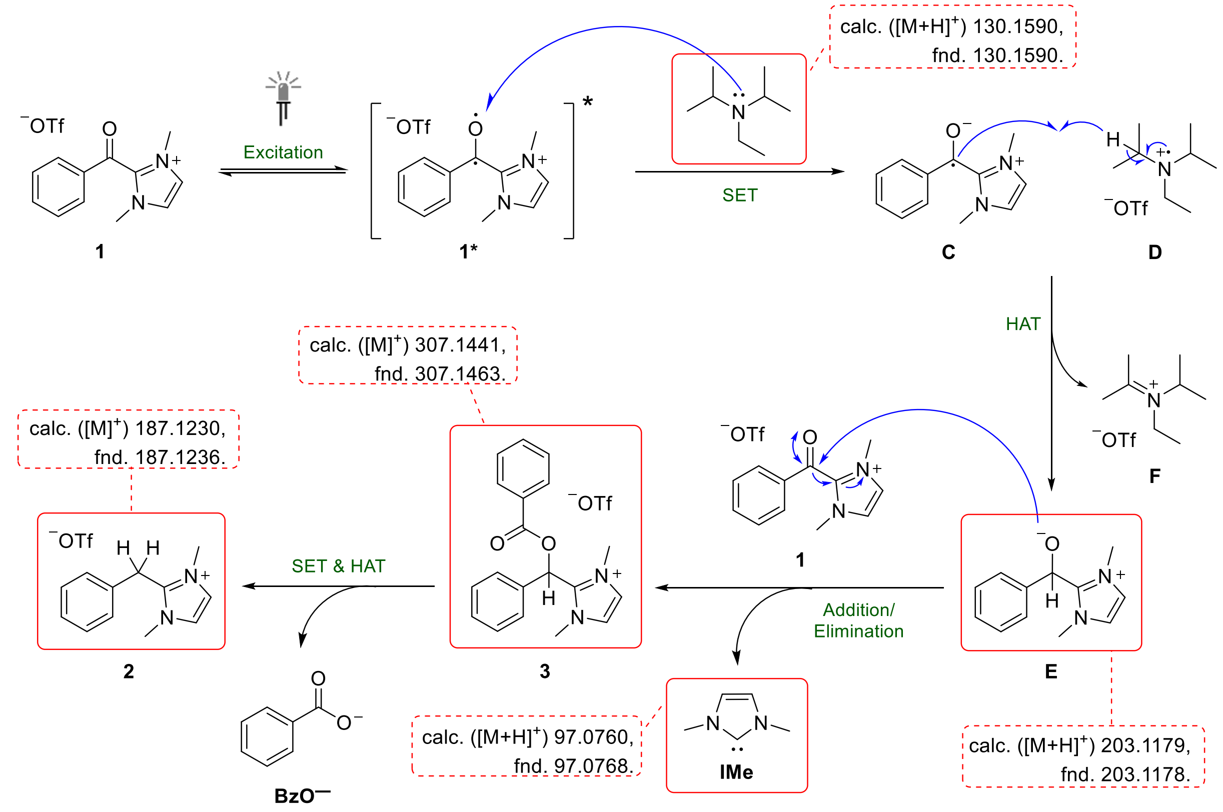

Figure S4: Working hypothesis for the non-photocatalytic UV-A reduction of acyl azolium **1** and ESI signals found in a crude reaction mixture (conditions: 1.0 equiv DIPEA, 0.05 M CH_3CN).

General procedure C:

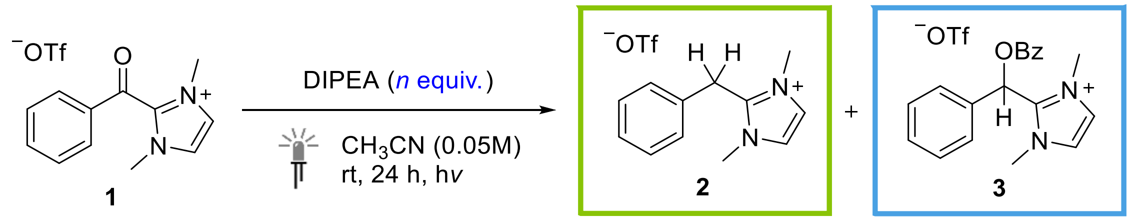

Acyl azolium **1** (35.0 mg, 0.100 mmol, 1.0 equiv) was added into a pre-dried Schlenk tube, which was then evacuated and backfilled with N_2 three times. Anh. CH_3CN (2.0 mL, 0.05 M) and diisopropylethylamine (DIPEA) either 1.0 equiv (17.0 μL, 0.100 mmol) or 5.0 equiv (85 μL, 0.50 mmol) were added. The mixture was allowed to stir under a nitrogen atmosphere, while irradiated at ambient temperature for 24 h. After switching off the light, the mixture was transferred into a flask (eluent: CH_2Cl_2) and the solvent was removed under reduced pressure. The obtained crude product was used as sample for ^{1}H NMR spectroscopy with dibromomethane (CH_2Br_2, 0.050 mmol) as internal standard.

3.1 NMR yield determination

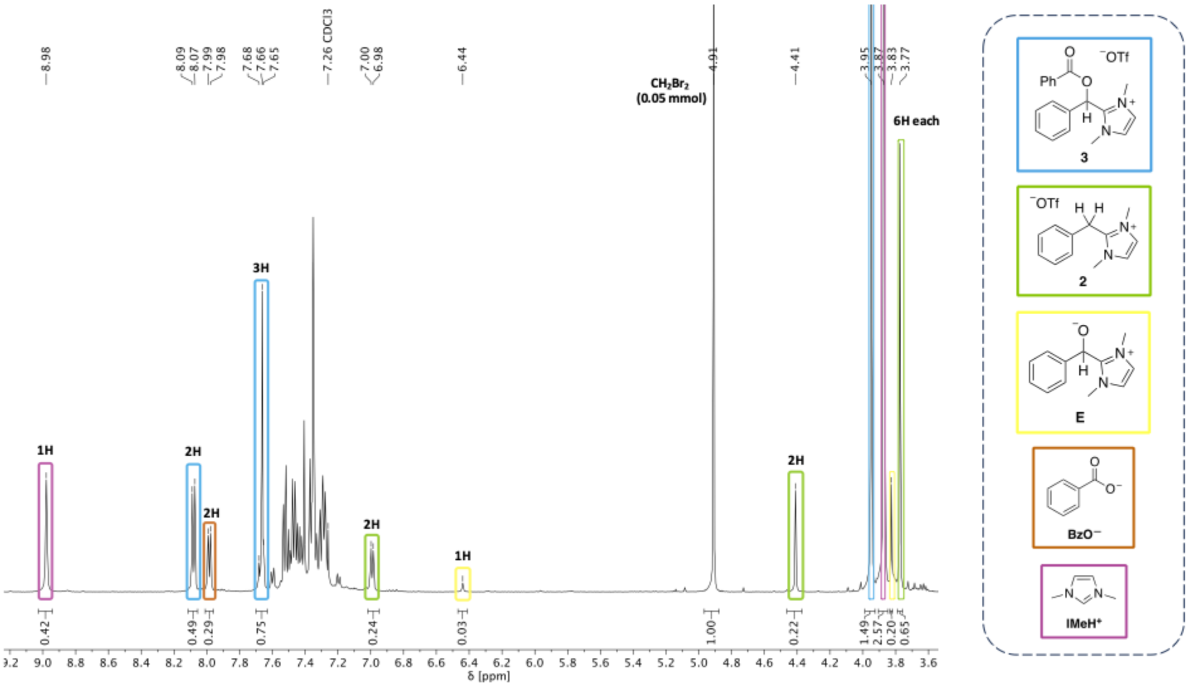

Figure S5: Typical signals in crude ^{1}H NMR spectra exemplified by the reaction shown in Table S3, entry 7. Dibromomethane (CH_2Br_2, 0.050 mmol) as internal standard (δ = 4.91 ppm).

3.2 Screening of reaction conditions

Table S1: Photocatalytic reduction with 5.0 equiv DIPEA (blue light)

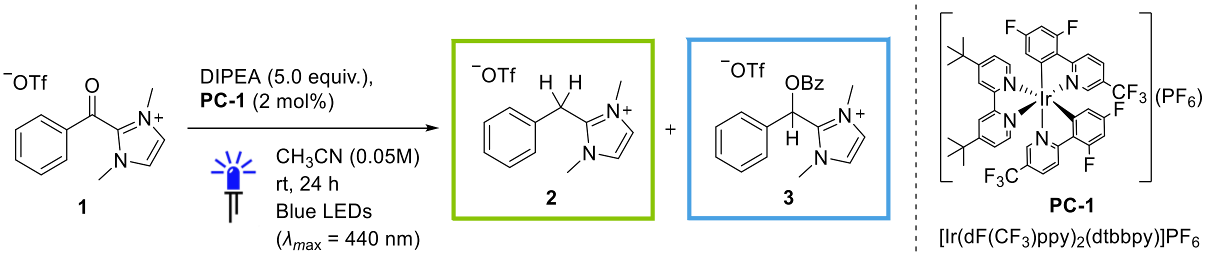

Entry	Variations from Reaction Conditions	NMR Yield[a]		
		Benzyl **2**	Benzoate **3**	Alkoxide **E**
1	*none*	39%	---	*traces*
2	no DIPEA	*n.d.*	*n.d.*	*n.d.*
3	in the dark		*no conversion*	
4	no photocatalyst	2%	15%	---
5	UV-A irradiation (λ_{max} = 370 nm) no photocatalyst	38%	9%	*traces*

n.d. = not determined (inconclusive); DIPEA = diisopropylethylamine; **PC-1**: 2.2 mg; [a] ^{1}H NMR yields determined with dibromomethane (CH_2Br_2, 0.050 mmol) as internal standard.

Table S2: Variation of DIPEA equivalents (UV-A irradiation)

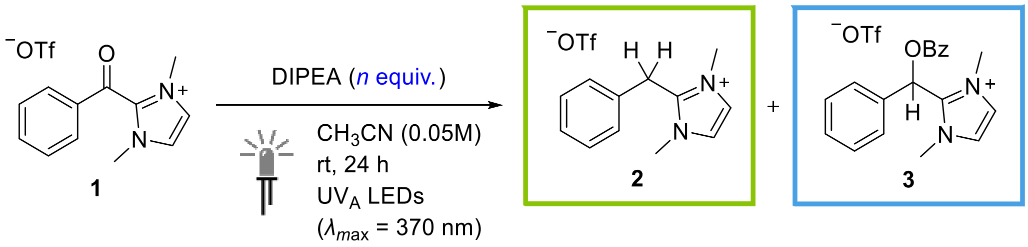

Entry	Equivalents of DIPEA	NMR Yield[a]		
		Benzyl **2**	Benzoate **3**	Alkoxide **E**
1	5.0 (85 µL)	38%	9%	*traces*
2	4.0 (68 µL)	29%	21%	---
3	3.0 (51 µL)	21%	21%	---
4	2.0 (34.0 µL)	24%	17%	---
5	1.0 (17.0 µL)	30%	9%	---

DIPEA = diisopropylethylamine; [a] ^{1}H NMR yields determined with dibromomethane (CH_2Br_2, 0.050 mmol) as internal standard.

Table S3: Variation of solvent using 1.0 equiv DIPEA (UV-A irradiation)

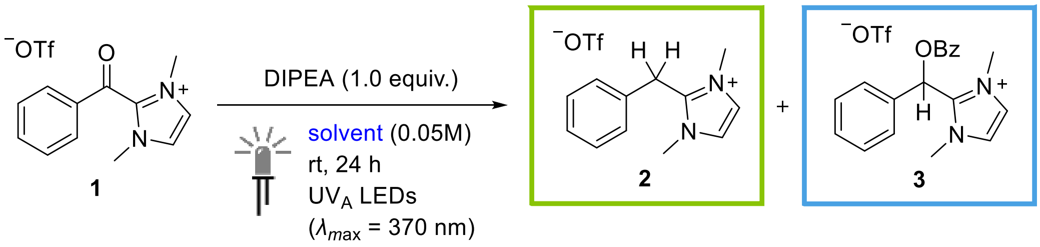

Entry	Solvent Variation	NMR Yield[a]		
		Benzyl **2**	Benzoate **3**	Alkoxide **E**
1	acetonitrile	30%	9%	---
2	dichloromethane (DCM)	6%	38%	1%
3	1,2-dichloroethane	6%	35%	1%
4	tetrahydrofuran (THF)	4%	7%	4%
5	acetone	14%	24%	---
6	*N*,*N*-dimethylformamide (DMF)	*n.d.*	*n.d.*	*n.d.*
7	trifluoromethylbenzene	11%	24%	3%
8	chlorobenzene	8%	38%	3%

n.d. = not determined (inconclusive); DIPEA = diisopropylethylamine; [a] ^{1}H NMR yields determined with dibromomethane (CH_2Br_2, 0.050 mmol) as internal standard.

Table S4: Further reactions using 1.0 equiv DIPEA (UV-A irradiation)

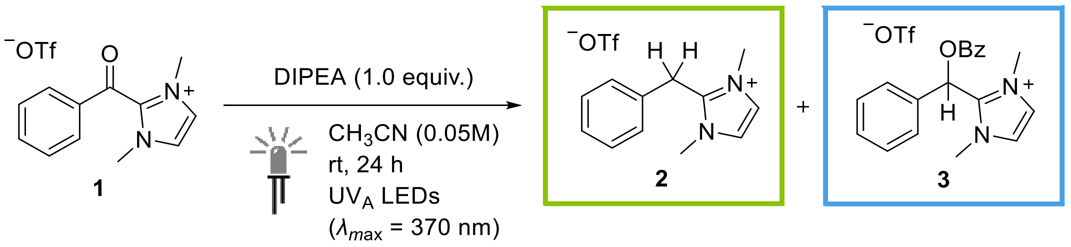

Entry	Solvent Variation	NMR Yield[a]		
		Benzyl **2**	Benzoate **3**	Alkoxide **E**
1	*none*	30%	9%	---
2	0.1 M (1.0 mL CH_3CN)	24%	22%	---
3	TEA as reductant (13.8 µL, 1.0 equiv)	27%	13%	---
4	H_2O (90 µL, 0.5 equiv)	*traces*	---	28%
5	no DIPEA	*n.d.*	*n.d.*	*n.d.*
6	in the dark	*no conversion*		

n.d. = not determined (inconclusive); DIPEA = diisopropylethylamine; TEA = triethylamine; [a] ^1H NMR yields determined with dibromomethane (CH_2Br_2, 0.050 mmol) as internal standard.

Table S5: Variation of photocatalyst using 1.0 equiv DIPEA (blue light)

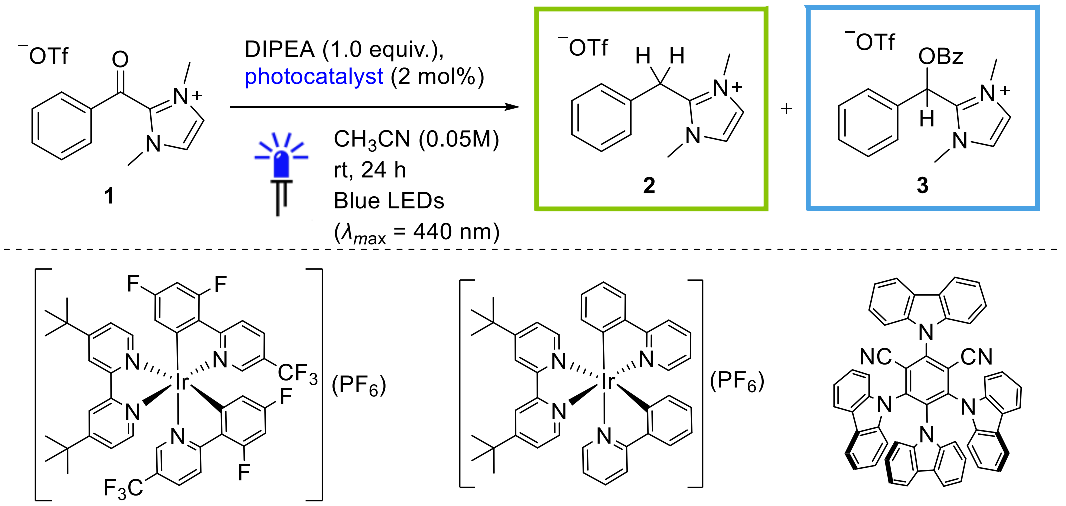

PC-1
$[Ir(dF(CF_3)ppy)_2(dtbbpy)]PF_6$

PC-2
$[Ir(ppy)_2(dtbbpy)]PF_6$

PC-3
4CzIPN

Entry	Photocatalyst Variation	NMR Yield[a]		
		Benzyl **2**	Benzoate **3**	Alkoxide **E**
1	**PC-1** (2.2 mg)	48%	---	---
2	**PC-2** (1.8 mg)	46%	*traces*	---
3	**PC-3** (1.6 mg)	50%	---	---
4	no photocatalyst	1%	11%	---

DIPEA = diisopropylethylamine; [a] ^1H NMR yields determined with dibromomethane (CH_2Br_2, 0.050 mmol) as internal standard.

3.3 Mechanistic experiments

Experiments on the mechanism of the UV-A photoreduction of acyl azolium **1** with DIPEA were performed. The progression of the photoreduction was followed by time-dependent reactions. Trapping experiments with trimethylsilylchloride (TMSCl) were conducted, to see if the formation of *O*-benzoylated azolium **3** proceeds via alkoxide **E**. Photoreactions of independently prepared azolium species **S1** and **3** were performed for reasoning the formation of fully reduced azolium **2**. Furthermore, UV–vis and voltammetry experiments with acyl azolium **1**, DIPEA and triethylsilane were conducted.
Results from TD-DFT calculations can be found in section *5 Computational Section*.

Time-dependency of UV-A-reduction of acyl azolium 1 using 1.0 equiv DIPEA

According to g*eneral procedure C*, a mixture of acyl azolium **1** (35.0 mg) and DIPEA (17.0 μL, 0.100 mmol, 1.0 equiv) in anh. CH_3CN (2.0 mL) was irradiated with UV-A light (λ_{max} = 370 nm) at ambient temperature for various reaction times.

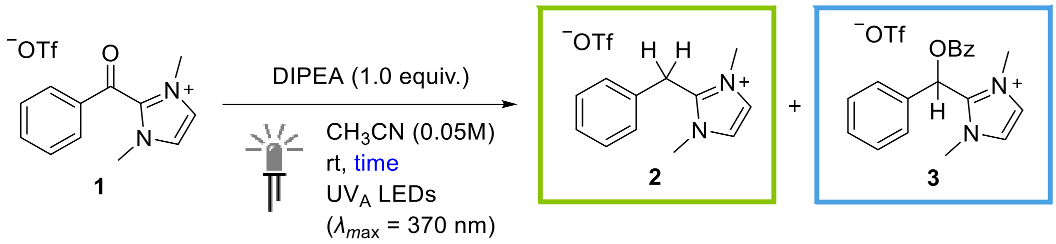

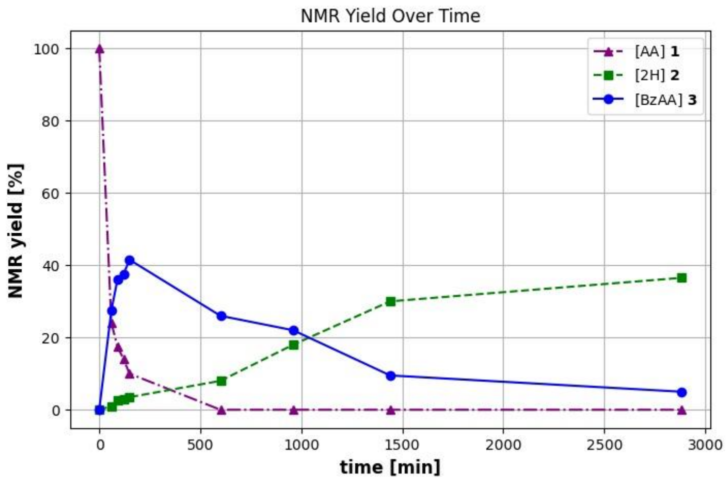

Time	NMR Yield[a]		
	1	2	3
1 h	24%	1%	27.5%
1.5 h	17.5%	2.5%	36%
2 h	14%	3%	37.5%
2.5 h	10%	3.5%	41.5%
10 h	---	8%	26%
16 h	---	18%	22%
24 h	---	30%	9.5%
48 h	---	36.5%	5%

Figure S6: Time-dependency of the UV-A-reduction of acyl azolium **1** with DIPEA; [a] ^{1}H NMR yields determined with dibromomethane (CH_2Br_2, 0.050 mmol) as internal standard.

Trapping experiments with trimethylsilylchloride (TMSCl)

Similar to *general procedure C*, a mixture of acyl azolium **1** (35.0 mg), DIPEA (17.0 µL, 0.100 mmol, 1.0 equiv) and TMSCl (15.2 µL, 0.120 mmol, 1.2 equiv) in anh. CH_3CN (2.0 mL) was irradiated with UV-A light (λ_{max} = 370 nm) at ambient temperature.

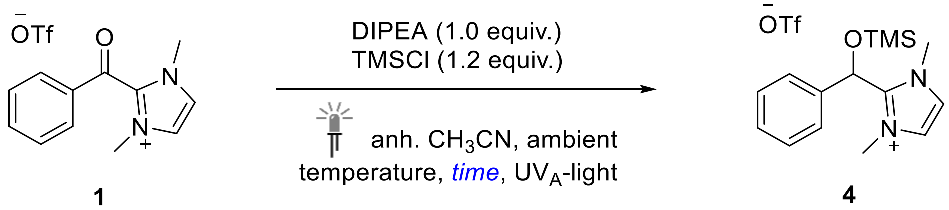

1 **4**

Entry	Time	NMR Yield[a]			
		Silyl Ether **4**	Unreacted **1**	Benzyl **2**	Benzoate **3**
1	15 min	31%	47%	---	---
2	24 h	62%	20%	---	---

DIPEA = diisopropylethylamine; TMSCl = trimethylsilylchloride; [a] ^{1}H NMR yields determined with dibromomethane (CH_2Br_2, 0.050 mmol) as internal standard.

Peak assignments for non-isolated[1] silyl ether **4** in crude ESI and ^{1}H NMR spectra (400 MHz, $CDCl_3$): δ = 7.63 (s, 2H), 7.43–7.31 (m, 3H), 7.21–7.25 (m, 2H), 6.29 (s, 1H), 3.89 (s, 6H), 0.13 (s, 9H) ppm; *m/z* calculated for $[C_{15}H_{23}N_2OSi]^+$ ($[M]^+$): 275.1574, found: 275.1579.

Alkoxide **E** + TMSCl:

A solution of independently prepared alcohol **S1** (17.8 mg, 0.050 mmol, 1.0 equiv), DIPEA (8.5 µL, 0.050 mmol, 1.0 equiv) and TMSCl (6.5 µL, 0.051 mmol, 1.0 equiv) in anh. CH_3CN (1.0 mL, 0.05 M) was allowed to stir for 1 h at room temperature. The solvent was removed under reduced pressure and the resulting crude product was analyzed by ^{1}H NMR spectroscopy.

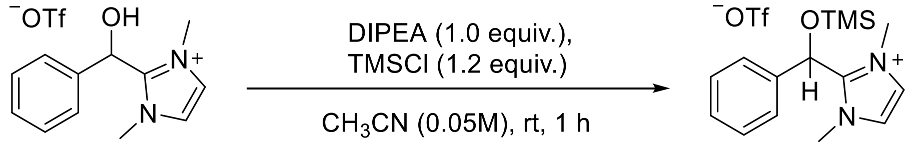

S1 **4**

20% (NMR)

*(80% alkoxide **E** / alcohol **S1**)*

[1] NB: Isolation of non-literature known silyl ether **4** from the reaction mixture failed. Attempts for the independent preparation via *N*-methylation of silyl ether **S4** did not yield silyl **4**, only afforded the desilylated alcohol **S1**.

Formation of *O*-benzoylated azolium 3 (addition–elimination step)

A solution of independently prepared alcohol **S1** (17.6 mg, 0.050 mmol, 1.0 equiv), DIPEA (8.5 µL, 0.050 mmol, 1.0 equiv) and acyl azolium **1** (17.5 mg, 0.050 mmol, 1.0 equiv) in anh. CH_3CN (1.0 mL, 0.05 M) was allowed to stir for 1 h at room temperature. The solvent was removed under reduced pressure and the resulting crude product was analyzed by ^1H NMR spectroscopy.

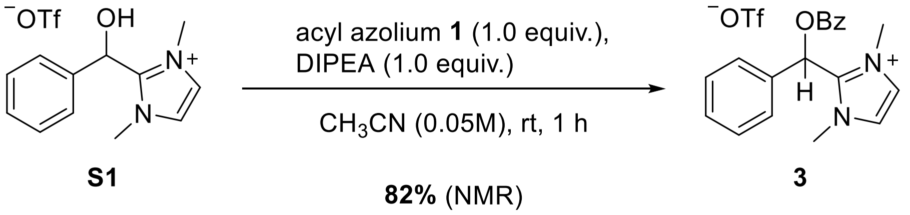

Table S6: Second photoreduction to fully reduced azolium 2

According to *general procedure C*, a mixture of either alcohol **S1** or benzoate **3** (0.100 mmol) and DIPEA (17.0 µL, 0.100 mmol, 1.0 equiv) in anh. CH_3CN (2.0 mL) was irradiated at ambient temperature for 24 h.

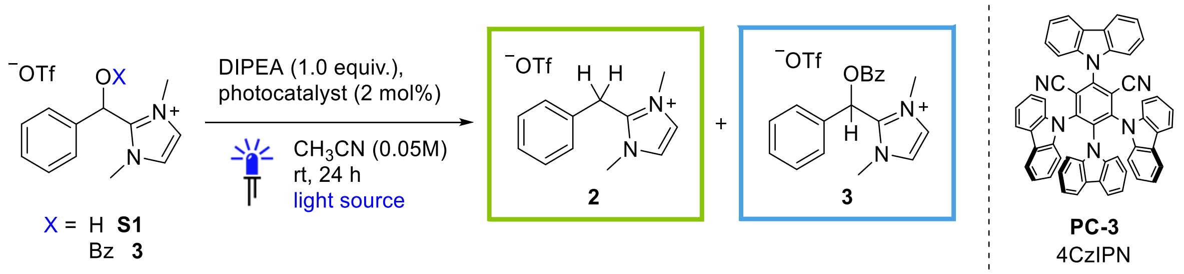

Entry	Reactant	Light Source [λ_{max}]	Photocatalyst	NMR Yield[a]		
				Alkoxide **E**	Benzoate **3**	Benzyl **2**
1	**S1** (35.2 mg)	370 nm	---	74%	3%	1%
2	**S1** (35.2 mg)	440 nm	**PC-3** (1.6 mg)	68%	---	10%
3	**3** (45.6 mg)	370 nm	---	*traces*	90%	8%
4	**3** (45.6 mg)	440 nm	**PC-3** (1.6 mg)	---	2%	77%

DIPEA = diisopropylethylamine; [a] ^1H NMR yields determined with dibromomethane (CH_2Br_2, 0.050 mmol) as internal standard.

UV–vis absorption spectra

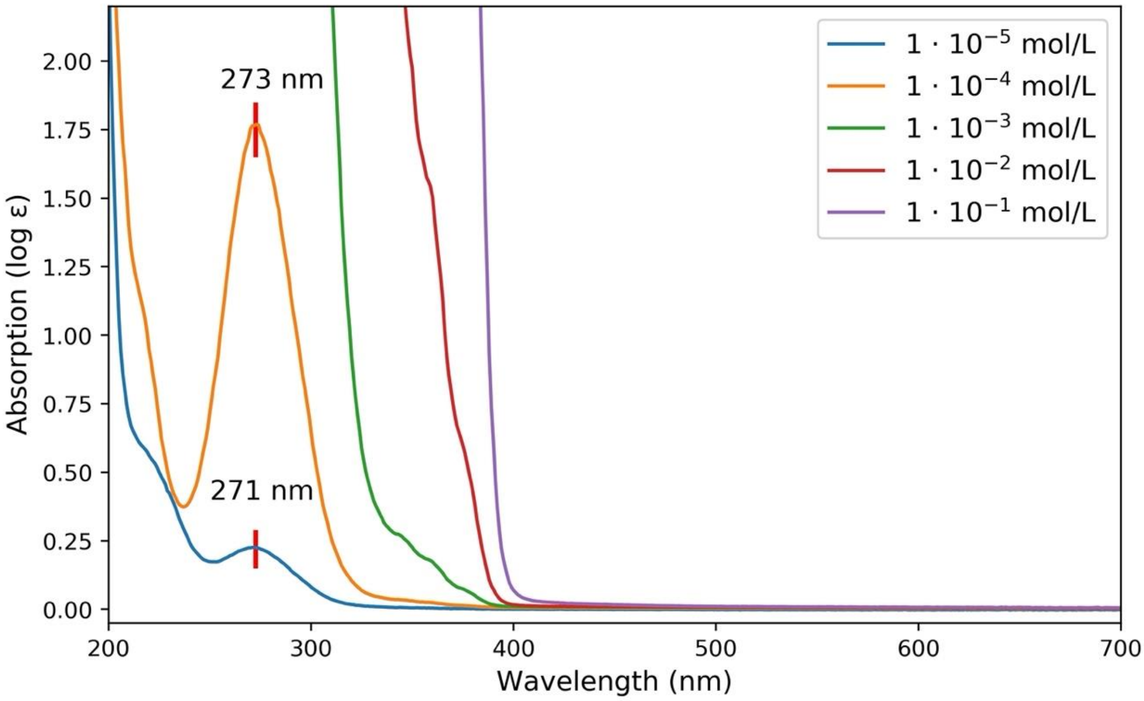

Figure S7: Stacked UV–vis absorption spectra of acyl azolium **1** at different concentrations in anh. CH_3CN.

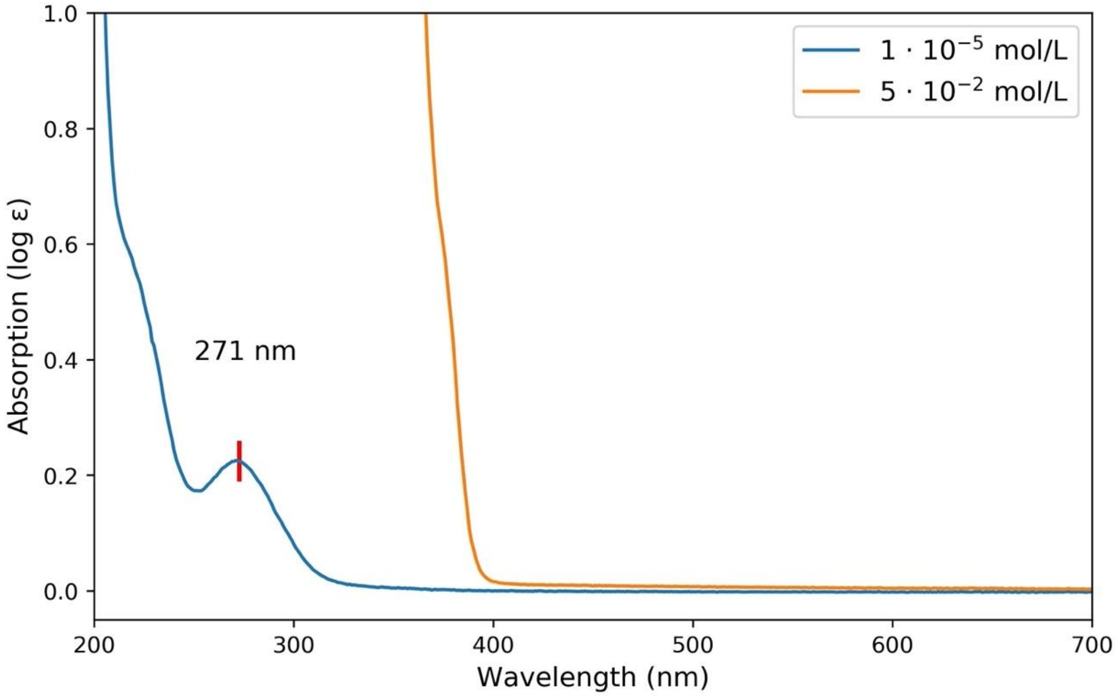

Figure S8: UV–vis absorption cut-off of acyl azolium **1** at the concentration of the photoreaction (0.05 M, anh. CH_3CN) and comparison with low concentration absorption.

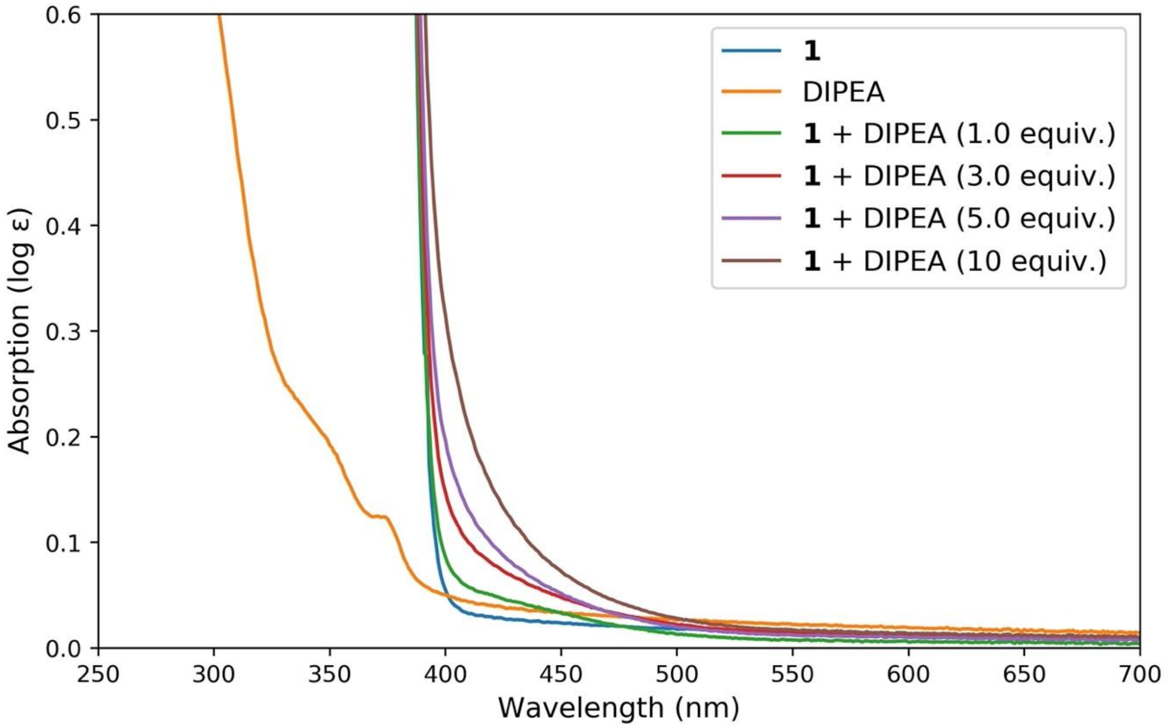

Figure S9: UV–vis absorption of acyl azolium **1** (0.05 M, anh. CH_3CN) with different equivalents of DIPEA and comparison with a 0.05 M DIPEA solution in anh. CH_3CN without azolium **1**.

Cyclic voltammetry (CV) & differential pulse voltammetry (DPV)

Cyclic voltammetry (CV) and differential pulse voltammetry (DPV) (Figure S10) were performed on an Autolab PGSTAT302N potentiostat using a three-electrode set-up: a freshly polished glassy carbon working electrode, a platinum wire counter electrode, and a silver wire pseudoreference electrode. All CV measurements were carried out three times with a scan rate of 100 mV/s. All DPV measurements were conducted with a scan rate of 10 mV/s (25 mV modulation amplitude, 50 ms modulation time, 5 mV step potential, 0.5 s interval time). The ferrocene/ferrocenium (Fc^0/Fc^+) couple was used as internal reference for the measurements of acyl azolium **1** and triethylsilane (TESH), whereas in case of DIPEA the decamethylferrocene/decamethylferrocenium ($DmFc^0/DmFc^+$) couple was used. Anhydrous and nitrogen-purged acetonitrile as solvent, n-Bu_4NPF_6 (0.1 M) as electrolyte, and an analyte concentration of 1.0 mM (acyl azolium **1** and DIPEA) or 0.6 mM (TESH) were used for all measurements. Collected and averaged data is shown in Table S7 and Table S8.

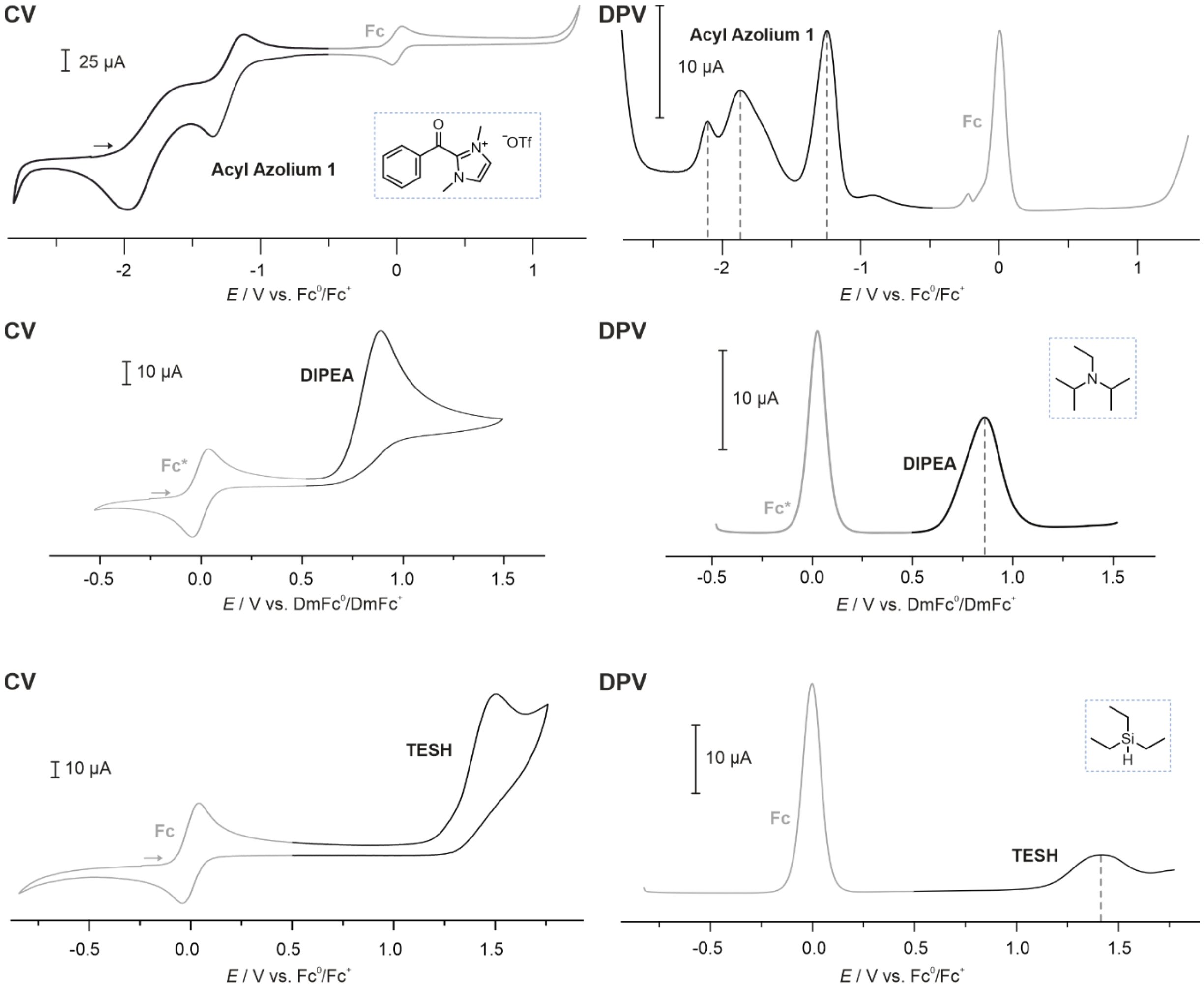

Figure S10: Voltammograms of (a) acyl azolium 1, (b) DIPEA, and (c) triethylsilane. Left: Cyclic voltammograms (CV, scan rate: 100 mV/s); right: differental pulse voltammograms (DPV, scan rate: 10 mV/s). Measurements conducted with a three-electrode set-up (glassy carbon working electrode, platinum counter electrode, silver pseudoreference electrode) in anh. acetonitrile with n-Bu_4NPF_6 (0.1 M) as electrolyte.

Table S7: Data collected using cyclic voltammetry (3 scans, 100 mV/s)

Compound	Scan 1	Scan 2	Scan 3	Mean	
Acyl Azolium **1** (1st Potential)					
E_p^A [V]	–1.174	–1.113	–1.144	–1.144 ± 0.031	vs Fc^0/Fc^+
E_p^C [V]	–1.369	–1.348	–1.352	–1.356 ± 0.012	vs Fc^0/Fc^+
$E_{1/2}$ [V]	–1.271	–1.230	–1.248	–1.250 ± 0.021	vs Fc^0/Fc^+
Acyl Azolium **1** (2nd Potential)					
E_p^C [V]	–1.953	–1.958	–1.974	–1.962 ± 0.012	vs Fc^0/Fc^+
DIPEA (1st Potential)					
E_p^A [V]	+0.880	+0.885	+0.893	+0.886 ± 0.007	vs $DmFc^0/DmFc^+$
TESH (1st Potential)					
E_p^A [V]	+1.506	+1.549	+1.575	+1.543 ± 0.035	vs Fc^0/Fc^+

E_p^A = anodic peak potential; E_p^C = cathodic peak potential; $E_{1/2}$ = (E_p^A + E_p^C)/2 = half wave potential; DIPEA = diisopropylethylamine; TESH = triethylsilane; Fc^0/Fc^+ = ferrocene/ferrocenium; $DmFc^0/DmFc^+$ = decamethylferrocene/decamethylferrocenium.

Table S8: Averaged peak and half-wave potentials from CV & DPV potentials

Compound	E_p^A [V]	E_p^C [V]	$E_{1/2}$ [V]	$E_{(DPV)}$ [V]	Reference
Acyl Azolium **1** (1st Potential)	–1.14 ± 0.03	–1.36 ± 0.01	–1.25 ± 0.02	–1.245	vs Fc^0/Fc^+
Acyl Azolium **1** (2nd Potential)	---	–1.96 ± 0.01	---	–1.872	vs Fc^0/Fc^+
DIPEA (1st Potential)	+0.89 ± 0.01	---	---	+0.833	vs $DmFc^0/DmFc^+$
	+0.38 ± 0.02	---	---	+0.328	vs Fc^0/Fc^+ [a]
TESH (1st Potential)	+1.54 ± 0.03	---	---	+1.417	vs Fc^0/Fc^+

E_p^A = anodic peak potential; E_p^C = cathodic peak potential; $E_{1/2}$ = (E_p^A + E_p^C)/2 = half wave potential; DIPEA = diisopropylethylamine; TESH = triethylsilane; Fc^0/Fc^+ = ferrocene/ferrocenium; $DmFc^0/DmFc^+$ = decamethylferrocene/decamethylferrocenium; [a] Converted using: E [V] vs $DmFc^0/DmFc^+$ − 0.51 V = E [V] vs Fc^0/Fc^+.[14]

For comparability of the obtained potentials, the measured anodic peak potential of DIPEA (E_p^A vs $DmFc^0/DmFc^+$) was converted as shown in Equation 1.

$$E_p^A = +0.89\ \text{V} - 0.51\ \text{V}^{[14]} = +0.38\ \text{V vs } Fc^0/Fc^+ \quad \text{(Eq. 1)}$$

Estimation of the excited-state potential of acyl azolium 1*

Similar to previous publications[15,16], the excited-state potential of acyl azolium **1** was estimated using Equation 2[17],

$$E(\mathbf{1^*/C}) = E(\mathbf{1/C}) + E_{0\text{-}0}(\mathbf{1^*/1}), \quad \text{(Eq. 2)}$$

in which the redox potential E(**1**/**C**) corresponds to the half-wave potential $E_{1/2}$ = −1.25 V (vs Fc^0/Fc^+) of acyl azolium **1**, measured by cyclic voltammetry (Figure S10, Tables S7–S8), and the excitation energy $E_{0\text{-}0}$(**1*/1**) corresponds to the absorption tail obtained by UV–vis spectroscopy (Figure S8), which is estimated to be λ = 420 nm and converts to 2.95 eV.

$$E(\mathbf{1^*/C}) = -1.25\ \text{V} + 2.95\ \text{V} = +1.70\ \text{V (vs Fc}^0\text{/Fc}^+) \quad \text{(Eq. 3)}$$

The estimated excited state potential of acyl azolium **1*** (Eq. 3) is higher than the anodic peak potentials (E_p^A, Table S8) of both DIPEA (+0.38 V vs Fc^0/Fc^+) and triethylsilane (+1.54 V vs Fc^0/Fc^+). Hence, a single electron transfer (SET) is thermodynamically feasible (Eq. 4).

$$\Delta G = -z\,F\,\Delta E, \text{ with } \Delta E = E_{\text{red}} - E_{\text{ox}} \quad \text{(Eq. 4)}$$

4 Photoreduction of acyl azolium 1 using triethylsilane as reductant

4.1 Screening of reaction conditions & isolation of silyl ether 5

General procedure D:

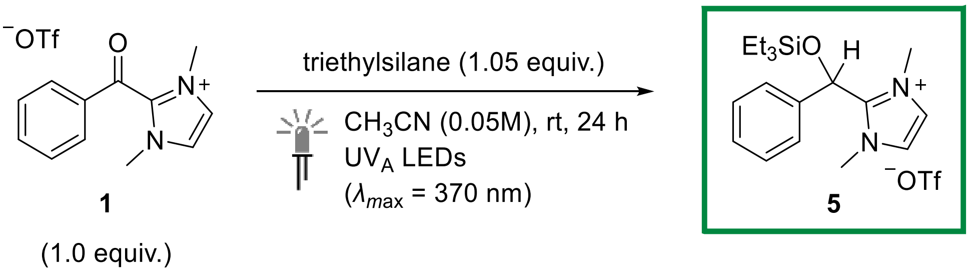

Acyl azolium **1** (35.0 mg, 0.100 mmol, 1.0 equiv) was added into a pre-dried Schlenk tube, which was then evacuated and backfilled with N_2 three times. Anh. CH_3CN (2.0 mL, 0.05 M) and triethylsilane (TESH, 16.7 µL, 0.105 mmol, 1.05 equiv) were added. The mixture was allowed to stir under a nitrogen atmosphere, while irradiated with UV-A light (λ_{max} = 370 nm) at ambient temperature for 24 h.
After switching off the light, the mixture was transferred into a flask (eluent: CH_2Cl_2) and the solvent was removed under reduced pressure. The obtained crude product was used as sample for ^{1}H NMR spectroscopy with dibromomethane (CH_2Br_2, 0.050 mmol) as internal standard.

Table S9: Screening of reaction conditions

Entry	Variation from Reaction Conditions	NMR Yield[a]		
		Silyl Ether **5**	Other Compounds	
1	*none*	62%	**S1** (7%)	**3** (6%)
2	dichloromethane (DCM) as solvent	---	**S1** (2%)	*n.d.*
3	2.0 equiv triethylsilane (31.8 µL)	59%	**S1** (6%)	**3** (5%)
4	only 20 h	57%	**S1** (*n.d.*)	**3** (5%)
5	only 10 h	52%	**S1** (*n.d.*)	**3** (5%)
6	0.1 M (1.0 mL CH_3CN)	61%	**S1** (*n.d.*)	**3** (5%)
7	4CzIPN (1.6 mg, 2 mol%), blue light (λ_{max} = 440 nm)	*5%*	**S1** (5%)	**1** (89%)
8	in the dark	*no conversion*		

n.d. = not determined (inconclusive); DIPEA = diisopropylethylamine; [a] ^{1}H NMR yields determined with dibromomethane (CH_2Br_2, 0.050 mmol) as internal standard.

1,3-Dimethyl-2-(phenyl((triethylsilyl)oxy)methyl)-1*H*-imidazol-3-ium trifluoromethane-sulfonate (5)

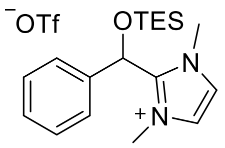

5

Similar to *general procedure D*, acyl azolium **1** (175.1 mg, 0.500 mmol, 1.0 equiv) and triethylsilane (84 µL, 61 mg, 0.52 mmoL, 1.0 equiv) were allowed to stir in anh. CH_3CN (10 mL) for 24 h while irradiated with UV-A light (λ_{max} = 370 nm) at ambient temperature. After switching off the light, the solvent was removed under reduced pressure. Purification via column chromatography (silica gel, dichloromethane/methanol 9:1) afforded silyl ether **5** as a colorless oil (138.3 mg, 0.296 mmol, 59%).

R_f: 0.45 (DCM/MeOH 9:1); **^{1}H NMR** (600 MHz, CDCl3): δ = 7.54 (s, 2H), 7.42–7.38 (m, 2H), 7.37–7.33 (m, 1H), 7.27 (dd, J = 7.4, 1.9 Hz, 2H), 6.28 (s, 1H), 3.92 (s, 6H), 0.87 (t, J = 8.0 Hz, 9H), 0.64 (qd, J = 7.9, 1.2 Hz, 6H) ppm; **^{19}F NMR** (376 MHz, $CDCl_3$): δ = −78.35 ppm; **^{13}C NMR** (151 MHz, $CDCl_3$): δ = 144.96, 136.24, 129.51, 125.43, 124.19, 121.85, 119.73, 66.94, 36.35, 6.44, 4.40 ppm; **HRMS-ESI:** *m/z* calculated for $[C_{18}H_{29}N_2OSi]^+$ ($[M]^+$): 317.2044, found: 317.2059; **IR (ATR):** $\tilde{\nu}$ = 3140 (w, ν(C–H)$_{arom}$), 2964 (w, ν(C–H)$_{aliph}$), 2884 (w, ν(C–H)$_{aliph}$), 1263 (vs, $\nu_{as}(SO_3)$), 1226 (m, $\nu_s(CF_3)$), 1033 (vs, $\nu_s(SO_3)$), 640 (s, $\delta_s(SO_3)$) cm^{-1}.

4.2 Further experiments

Further acyl azolium salts tested

According to *general procedure D*, acyl azolium **6–9** (0.100 mmol, 1.0 equiv) and triethylsilane (TESH, 16.7 µL, 0.105 mmol, 1.05 equiv) in anh. CH_3CN (2.0 mL, 0.05 M) were irradiated with UV-A light (λ_{max} = 370 nm) at ambient temperature for 24 h.

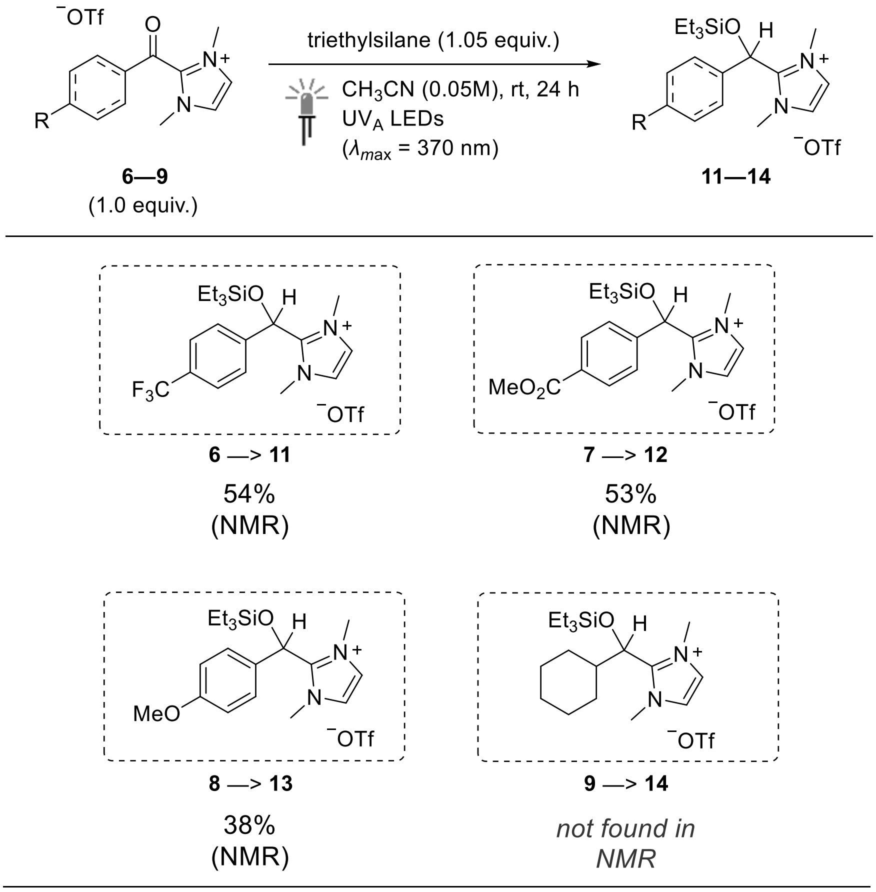

Figure S11: Photoreduction of other acyl azolium salts **6**–**9** with triethylsilane to silyl ethers **11**–**14**; ^{1}H NMR yields determined with dibromomethane (CH_2Br_2, 0.050 mmol) as internal standard.

<u>Peak assignments for non-isolated silyl ethers **11**–**14**:</u>

Silyl ether **11** in crude ^{1}H NMR spectrum (400 MHz, $CDCl_3$): δ = 7.68 (d, J = 8.3 Hz, 2H), 7.53 (s, 2H), 7.48 (d, J = 8.2 Hz, 2H), 0.89 (t, J = 7.8 Hz, 9H), 0.66 (qd, J = 8.5, 7.9, 2.9 Hz, 6H) ppm.

Silyl ether **12** in crude ^{1}H NMR spectrum (400 MHz, $CDCl_3$): δ = 7.68 (d, J = 8.3 Hz, 2H), 7.53 (s, 2H), 7.48 (d, J = 8.2 Hz, 2H), 0.89 (t, J = 7.8 Hz, 9H), 0.66 (qd, J = 8.4, 7.8, 2.1 Hz, 6H) ppm.

Silyl ether **13** in crude ESI and ^{1}H NMR spectrum (500 MHz, $CDCl_3$): δ = 7.52 (s, 2H), 7.18 (d, J = 8.8 Hz, 2H), 6.91 (d, J = 8.8 Hz, 2H), 6.20 (s, 1H), 3.91 (s, 6H), 3.86 (s, 3H), 0.86 (t, J = 7.9 Hz, 9H), 0.62 (q, J = 7.6 Hz, 6H) ppm; *m/z* calculated for $[C_{19}H_{31}N_2O_2Si]^+$ ($[M]^+$): 347.2149, found: 347.2146.

Silyl ether **14** only in crude ESI: *m/z* calculated for $[C_{18}H_{35}N_2OSi]^+$ ($[M]^+$): 323.2513, found: 323.2517.

Desilylation of crude silyl ether 5

A crude sample of silyl ether **5**, obtained after following the standard conditions described in *general procedure D*, was dissolved in CD_3CN (2.0 mL) and cesium fluoride (23 mg, 0.15 mmol, 1.5 equiv) was added. The mixture was allowed to stir for 4.5 h at room temperature. Dibromomethane (CH_2Br_2, 7.0 µL, 0.10 mmol) was added as internal standard and 0.7 mL of the solution was used as sample for ^{1}H NMR spectroscopy.

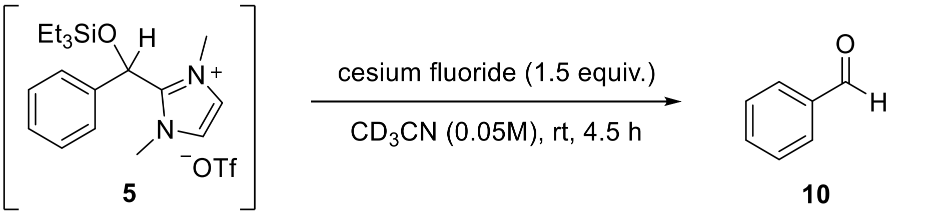

5 (*crude*) (1.0 equiv.) → **10** 56% (NMR) (+ unreacted **5** 2%)

Peak assignments for non-isolated benzaldehyde **10** in crude ^{1}H NMR spectrum (500 MHz, CD_3CN): δ = 10.00 (s, 1H), 7.92–7.88 (m, 2H), 7.71–7.67 (m, 1H), 7.59 (dd, J = 8.4, 7.0 Hz, 2H) ppm.

"One-pot" triethylsilane photoreduction desilylation:

Similar to *general procedure D*, acyl azolium **1** (35.0 mg, 0.100 mmol, 1.0 equiv) and triethylsilane (TESH, 16.7 µL, 0.105 mmol, 1.05 equiv) in CD_3CN (2.0 mL) was irradiated with UV-A light (λ_{max} = 370 nm) for 24 h at ambient temperature. After switching off the light, cesium fluoride (30.3 mg, 0.199 mmol, 2.0 equiv) was added and the mixture was allowed to stir at room temperature for 4.5 h under a nitrogen atmosphere. Dibromomethane (CH_2Br_2, 7.0 µL, 0.10 mmol) was added as internal standard and 0.7 mL of the solution was used as sample for ^{1}H NMR spectroscopy.

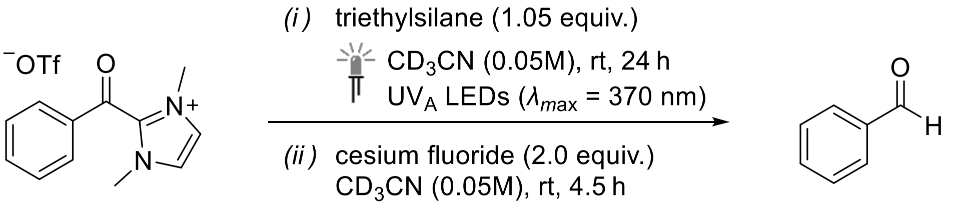

1 (1.0 equiv.) → **6** 20% (NMR) (+ unidentified aromatic by products)

"One-Pot"

Trapping experiment with trimethylsilylchloride (TMSCl)

According to *general procedure D*, azolium **1** (35.0 mg, 0.100 mmol, 1.0 equiv) in anh. CH_3CN (2.0 mL) was irradiated with UV-A light (λ_{max} = 370 nm) for 24 h in the presence of triethylsilane (TESH, 16.7 µL, 0.105 mmol, 1.05 equiv) and TMSCl (15.2 µL, 0.120 mmol, 1.2 equiv) at ambient temperature. The resulting crude mixture was analyzed by ^{1}H NMR spectroscopy.

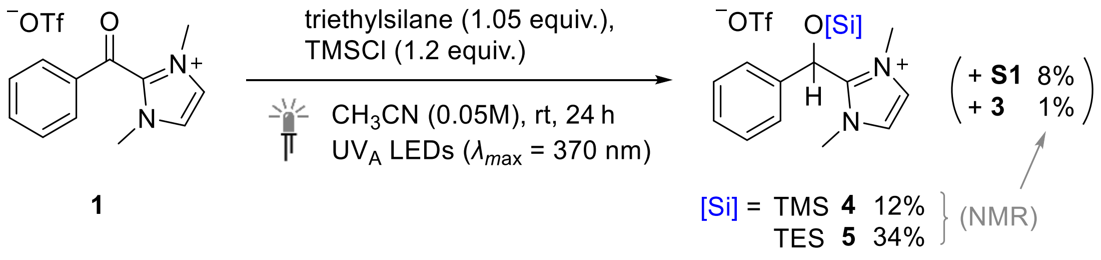

5 Computational section

5.1 Conformer search with CREST

Conformational ensemble generation was performed using the CREST[18] (conformer-rotamer ensemble sampling tool) software package. CREST utilizes semiempirical quantum chemical methods to explore conformational space and identify low-energy structures corresponding to minima on the potential energy surface (PES). Prior to quantum chemical calculations (see Section 5.2), conformer searches were conducted for the system of acyl imidazolium **1** with DIPEA. All molecular structures were initially constructed using Avogadro[19].

For the complex of alcoholate **E** and oxidized DIPEA-cation **F** (**E**–**F** complex) involved in the first reaction, the conformer search was carried out using CREST with the ALPB[20] implicit solvation model and acetonitrile (MeCN) as the solvent. Calculations were performed at the GFN2-xTB[21] level of theory. A total molecular charge of +1 was assigned to the complex. As the system involved non-covalently bound components, the NCI (non-covalent interactions) option was enabled. The energy window was set to 12 kcal/mol. A harmonic constraint (force constant: 0.50 Eh/Bohr^2) was applied to the oxidized DIPEA-cation **F** fragment to maintain its geometry during sampling. Two unique conformers were identified; the one with the lowest energy was selected for further quantum chemical analysis. The optimized structure of this lowest- energy conformer is shown in Figure S12.

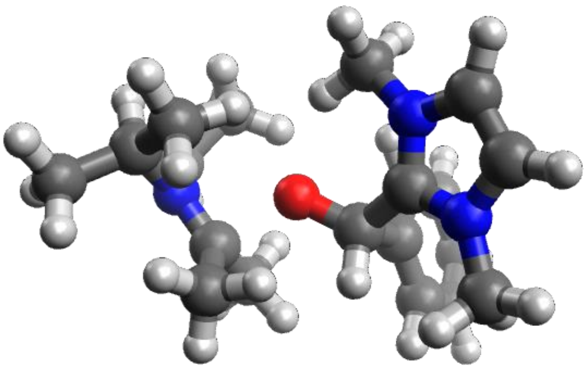

Figure S12: Chemical structure of the most stable conformer of the **E**–**F** complex.

5.2 Quantum chemical calculations with Gaussian

All quantum chemical calculations, including geometry optimizations, single-point energy computations, and time-dependent simulations, were carried out using the Gaussian16[22] software package (Revision A.03). The computational protocol followed the methodology reported by Mavroskoufis et al.[23] to ensure consistency with previous studies.

Calculations were performed using density functional theory (DFT) and time-dependent DFT (TD-DFT)[24] employing the CAM-B3LYP[25] functional and the 6-31+G(d)[26,27,28] basis set. Solvent effects were considered using the CPCM[29] implicit solvation model with acetonitrile (ε = 35.688), in line with the experimental conditions. No symmetry constraints were applied during geometry optimizations (i.e., Symmetry=None). For TD-DFT calculations, five excited states were computed by default. This number was increased to 12 for calculations involving natural transition orbitals (NTOs).

Calculated UV spectra: Two types of UV–vis spectral calculations were performed: vertical excitation (absorption) and vertical de-excitation (emission). For the vertical excitation spectra, geometry optimizations of both acyl imidazolium **1** and the **1**–DIPEA complex were carried out in the ground electronic state (S_0), followed by TD-DFT calculations to compute the excitation energies. For the vertical de-excitation spectra, the geometries of both species were first optimized in the first excited state (S_1), using the ground-state optimized structures as initial guesses. Subsequent TD-DFT calculations were then performed to obtain the emission energies.

Relaxed scan calculation: A relaxed minimum energy pathway (MEP) scan was performed to investigate the hydrogen atom transfer (HAT) process along the C–H bond of the alcoholate **E** fragment, specifically the hydrogen atom attached to the carbon of the carbonyl group. During the scan, the C–H bond was incrementally elongated. All calculations were carried out in the first excited state (S_1) using TD-DFT at the CAM- B3LYP/6-31+G(d) level of theory, with acetonitrile (MeCN) as the implicit solvent. At each point along the relaxed scan, the electronic energies of the ground state (S_0), first excited state (S_1), and second excited state (S_2) were evaluated at the corresponding S_1-optimized geometries. The scan consisted of 20 steps with an increment of 0.2 Å. As an initial structure, the **E**–**F** complex with a center-of-mass separation of 6.66 Å was used. The starting C–H bond length was 1.11 Å. In addition, Mulliken population analysis was performed using Gaussian to monitor the redistribution of electronic charge between the alcoholate **E** and oxidized DIPEA-cation **F** fragments throughout the HAT process, providing insight into the nature of the charge transfer during the reaction pathway.

Reduction potential of acyl imidazolium **1** and *O*-benzoylated species **3**: The reduction potentials of two species, acyl imidazolium **1** (the reactant) and benzoate **3** (the corresponding product of the first reduction process), were computed using a thermodynamic cycle approach. For each species, geometry optimization and frequency analysis (Opt+Freq) were performed in the gas phase at the ground electronic state (S_0), followed by a single-point energy calculation in acetonitrile (MeCN) using the CPCM implicit solvation model. Each calculation was performed twice per species: once for the oxidized form (charge = +1) and once for the reduced form (charge = 0). Vibrational frequency calculations provided the zero-point vibrational energy (ZPVE) and the thermal correction to the Gibbs free energy. This methodology was applied to both **1** and **3**.

5.3 NTO calculations with ORBKIT

Post-processing of electronic structure data was conducted using ORBKIT[30], an open-source Python-based toolkit designed for visualizing and analyzing molecular orbitals and electron densities. Output files from the TD-DFT single-point calculations were used as input for ORBKIT. A modified version of the program, provided by Dr. Fabian Weber, was used to compute the NTOs for excited-state analysis. In total, 12 excited states were analyzed for NTO construction.

5.4 Structures

Optimized XYZ geometry of acyl imidazolium 1 (S_0 state) in MeCN

C -0.003333 0.028199 0.057471
N 0.028376 0.003197 1.430073
C 1.308384 -0.031806 1.828205
N 2.085890 -0.035936 0.737073
C 1.284462 0.010064 -0.375161
C 1.839873 -0.161657 3.232015
O 2.640387 -1.058686 3.431672
C 3.550882 -0.097104 0.708885
C -1.172059 -0.026538 2.272866
C 1.399899 0.791951 4.264029
C 0.854947 2.035932 3.924437
C 0.499953 2.931820 4.925411
C 0.675230 2.583719 6.262762
C 1.217092 1.343702 6.605283
C 1.586801 0.451764 5.610311
H 0.728609 2.320130 2.884035
H 0.087941 3.900236 4.662061
H 0.389917 3.281837 7.043615
H 1.350097 1.077168 7.648635
H 2.012733 -0.514119 5.859600
H 1.694033 0.028847 -1.372677
H -0.932628 0.049126 -0.489591
H -0.914962 -0.381397 3.267741
H -1.887666 -0.712120 1.820813
H -1.600439 0.974208 2.334342
H 3.876144 -1.130950 0.820853
H 3.960594 0.511084 1.513164
H 3.882806 0.299867 -0.248581

Optimized XYZ geometry of acyl imidazolium 1 (S_1 state) in MeCN

C -0.019989 -0.593086 0.241211
N 0.119911 -0.026217 1.488798
C 1.446374 0.168702 1.729955
N 2.125568 -0.275724 0.636238
C 1.218217 -0.747262 -0.285438
C 2.040956 0.720005 2.875794
O 3.309677 0.884487 3.057823
C 3.573228 -0.261581 0.454961
C -0.991171 0.297276 2.375345
C 1.442401 1.240095 4.126672
C 1.140558 2.614404 4.237414
C 0.559061 3.087791 5.399180
C 0.338863 2.216035 6.471088
C 0.704928 0.867974 6.391883
C 1.287272 0.381380 5.236052
H 1.335125 3.274528 3.399138
H 0.283611 4.133273 5.482968
H -0.105634 2.595634 7.385304
H 0.541425 0.209455 7.237782
H 1.593440 -0.656079 5.156904
H 1.532917 -1.150732 -1.234356
H -0.990488 -0.836450 -0.160422
H -0.910250 -0.260043 3.309433
H -1.911985 0.010675 1.869792
H -1.017238 1.368058 2.581292
H 4.056951 -0.866022 1.223334
H 3.949962 0.761227 0.495910
H 3.790272 -0.685263 -0.524710

Optimized XYZ geometry of alcoholate E in MeCN

H -0.799838 -0.383963 1.250448
C -1.707556 0.064429 0.854457
C -2.855987 -0.711649 0.733230
H -2.840414 -1.752548 1.044125
C -4.025605 -0.155190 0.214234
H -4.922424 -0.760315 0.118370
C -4.034840 1.180079 -0.179119
H -4.940159 1.622768 -0.585035
C -2.880909 1.953102 -0.050876
H -2.893479 2.996013 -0.360168
C -1.705520 1.406782 0.464405
C -0.417715 2.248175 0.506931
C 0.137083 2.204726 1.943532
N 1.184334 1.509998 2.410727

N -0.352130 2.918918 2.974113
C 1.357574 1.785285 3.753078
C 0.395828 2.668322 4.106523
O 0.491104 1.897456 -0.430760
C -1.493742 3.827387 2.915837
C 2.021091 0.564621 1.667445
H 2.149163 1.329428 4.326567
H 0.179032 3.143004 5.050412
H -1.279776 4.658962 2.242295
H -1.670439 4.213014 3.918639
H -2.380654 3.294579 2.572002
H 1.777857 0.687581 0.612240
H 1.808434 -0.450662 2.008660
H 3.068720 0.801909 1.859582
H -0.756365 3.304378 0.409595

Optimized XYZ geometry of DIPEA in MeCN

C 1.199290 3.513135 0.347993
C -0.129759 2.812567 0.654438
N -0.131328 1.345870 0.635697
C 0.623137 0.783947 1.783930
C 2.034492 0.261946 1.476150
C 0.100548 0.731965 -0.684201
C -0.340708 -0.734930 -0.709620
H 1.057024 4.599885 0.351486
H 1.591002 3.230683 -0.634976
H 1.963177 3.276101 1.095416
H -0.893999 3.170994 -0.038927
H -0.471061 3.120207 1.649216
H 2.026745 -0.650416 0.872703
H 2.542293 0.025079 2.417213
H 2.636933 1.008806 0.951047
H -0.186767 -1.147062 -1.712576
H -1.405588 -0.815817 -0.463677
H 0.216669 -1.362469 -0.013416
C -0.653029 1.466610 -1.796955
H -0.278783 2.477588 -1.975272
H -1.723300 1.528853 -1.567856
H -0.540185 0.909672 -2.732117
C -0.195324 -0.249946 2.566739
H 0.762270 1.625916 2.470526
H -0.365467 -1.167726 1.998485
H -1.171006 0.166935 2.835331
H 0.326875 -0.521299 3.491888
H 1.172550 0.762038 -0.945696

Optimized XYZ geometry of oxidized DIPEA-cation F in MeCN

C 1.136927 3.613655 0.346280
C -0.115112 2.800900 0.646793
N 0.117942 1.340012 0.511582
C 0.769156 0.738467 1.729949
C 2.046044 -0.041184 1.424534
C -0.229442 0.701139 -0.559185
C -0.110016 -0.775801 -0.722076
H 0.910848 4.674194 0.485246
H 1.472763 3.466615 -0.683765
H 1.960088 3.354316 1.017532
H -0.941723 3.087797 0.001572
H -0.446427 2.958782 1.674910
H 1.869868 -1.040901 1.027312
H 2.596333 -0.154779 2.362692
H 2.685048 0.506503 0.726322
H 0.738761 -0.986007 -1.383516
H -1.006259 -1.132845 -1.236671
H 0.011287 -1.330568 0.200892
C -0.803622 1.422219 -1.739836
H -0.314583 2.375766 -1.938121
H -1.870921 1.608198 -1.569674
H -0.717223 0.791668 -2.625311
C -0.249714 -0.005818 2.589374
H 1.079346 1.619483 2.292349
H -0.605182 -0.927487 2.124267
H -1.114705 0.626892 2.807382
H 0.224188 -0.269804 3.538678

Optimized XYZ geometry of 1–DIPEA complex (S_0 state) in MeCN

H -0.164886 1.473719 1.002113
C -0.915395 1.922010 0.359824
C -0.811557 3.246565 -0.036275
H 0.027086 3.848075 0.298893
C -1.785235 3.803792 -0.867153
H -1.699472 4.840113 -1.178824
C -2.865145 3.038049 -1.300522
H -3.615035 3.470670 -1.954328
C -2.981658 1.714171 -0.895000
H -3.817432 1.118064 -1.249059
C -2.006471 1.151663 -0.063120
C -2.068491 -0.262290 0.340909
C -3.381608 -0.996624 0.250054
N -4.549347 -0.661901 0.816798
N -3.532528 -2.184593 -0.350164
C -5.461684 -1.656185 0.561834

C -4.826183 -2.605692 -0.173736
O -1.109066 -0.902341 0.733712
C -2.495465 -2.938036 -1.062368
C -4.837426 0.529919 1.621932
H -6.474302 -1.606529 0.929859
H -5.183759 -3.536612 -0.584353
H -1.852393 -3.444030 -0.343094
H -1.905676 -2.262128 -1.679447
H -2.991558 -3.667346 -1.699830
H -5.289674 1.299601 0.996050
H -3.918760 0.904251 2.068307
H -5.527581 0.241766 2.413275
C 2.144512 -0.465352 -1.865409
C 3.432020 0.242238 -1.454975
N 3.942547 -0.215088 -0.150609
C 4.832476 -1.381769 -0.365533
C 4.798728 -2.416792 0.763161
C 4.426080 0.905460 0.682237
C 5.089664 0.468726 1.989593
H 1.821788 -0.132349 -2.858739
H 1.343015 -0.251424 -1.150908
H 2.278545 -1.551820 -1.904597
H 3.245986 1.316210 -1.421205
H 4.193200 0.103212 -2.238697
H 5.346057 -2.106211 1.654735
H 5.251918 -3.350120 0.409715
H 3.765022 -2.629539 1.052651
H 4.406834 -0.146206 2.585344
H 5.333160 1.361620 2.574231
H 6.017583 -0.084585 1.845842
C 3.257927 1.823904 1.065719
H 2.708464 2.218150 0.208414
H 2.549155 1.277315 1.698309
C 6.270558 -1.036189 -0.783018
H 4.382506 -1.892752 -1.223300
H 6.855565 -0.611553 0.037315
H 6.285892 -0.320425 -1.611532
H 6.786368 -1.941977 -1.119625
H 5.163389 1.505959 0.117078
H 3.632930 2.681279 1.634609

Optimized XYZ geometry of 1–DIPEA complex (S_1 state) in MeCN

H -0.161084 1.584280 1.010663
C -0.934412 2.005673 0.377610
C -0.883476 3.338638 -0.008157
H -0.059129 3.962459 0.322880
C -1.886796 3.876386 -0.812709

H -1.843456 4.919194 -1.111333
C -2.944421 3.070340 -1.237832
H -3.722865 3.482435 -1.871711
C -3.006994 1.733197 -0.868149
H -3.820924 1.113090 -1.230203
C -2.004647 1.195708 -0.045527
C -2.017785 -0.218903 0.343576
C -3.236414 -1.034354 0.249156
N -4.443609 -0.759464 0.776399
N -3.290492 -2.258290 -0.308838
C -5.290262 -1.804226 0.489907
C -4.564614 -2.749868 -0.165662
O -0.976769 -0.830472 0.738942
C -2.180743 -2.955309 -0.960365
C -4.821531 0.430062 1.538645
H -6.325337 -1.792986 0.792858
H -4.852498 -3.712440 -0.557856
H -1.524961 -3.397251 -0.209494
H -1.624349 -2.246720 -1.574063
H -2.597989 -3.734682 -1.594960
H -5.276735 1.167587 0.876409
H -3.933469 0.848914 2.008460
H -5.531961 0.127941 2.306874
C 1.912380 -0.528248 -1.717403
C 3.238082 0.179430 -1.453525
N 3.831171 -0.192564 -0.157867
C 4.672019 -1.401558 -0.334268
C 4.673303 -2.347896 0.870370
C 4.380354 0.970240 0.570078
C 5.104561 0.609034 1.868486
H 1.527277 -0.263529 -2.708677
H 1.167848 -0.240977 -0.967799
H 2.023588 -1.617321 -1.681525
H 3.075137 1.256774 -1.488962
H 3.939956 -0.038547 -2.273143
H 5.280775 -1.991082 1.703588
H 5.077192 -3.319041 0.562851
H 3.652422 -2.503889 1.232543
H 4.443287 0.051890 2.540337
H 5.394638 1.534618 2.375456
H 6.013470 0.027353 1.716302
C 3.248808 1.936095 0.946603
H 2.665197 2.282094 0.091141
H 2.562869 1.440930 1.642561
C 6.091851 -1.133048 -0.857071
H 4.157800 -1.958750 -1.124211
H 6.736099 -0.669167 -0.105535
H 6.078629 -0.481355 -1.736836
H 6.559178 -2.078284 -1.152724

H 5.096132 1.512139 -0.075329
H 3.662338 2.821950 1.439474

Optimized XYZ geometry of E–F complex in MeCN

H -0.164886 1.473719 1.002113
C -0.915395 1.922010 0.359824
C -0.811557 3.246565 -0.036275
H 0.027086 3.848075 0.298893
C -1.785235 3.803792 -0.867153
H -1.699472 4.840113 -1.178824
C -2.865145 3.038049 -1.300522
H -3.615035 3.470670 -1.954328
C -2.981658 1.714171 -0.895000
H -3.817432 1.118064 -1.249059
C -2.006471 1.151663 -0.063120
C -2.068491 -0.262290 0.340909
C -3.381608 -0.996624 0.250054
N -4.549347 -0.661901 0.816798
N -3.532528 -2.184593 -0.350164
C -5.461684 -1.656185 0.561834
C -4.826183 -2.605692 -0.173736
O -1.109066 -0.902341 0.733712
C -2.495465 -2.938036 -1.062368
C -4.837426 0.529919 1.621932
H -6.474302 -1.606529 0.929859
H -5.183759 -3.536612 -0.584353
H -1.852393 -3.444030 -0.343094
H -1.905676 -2.262128 -1.679447
H -2.991558 -3.667346 -1.699830
H -5.289674 1.299601 0.996050
H -3.918760 0.904251 2.068307
H -5.527581 0.241766 2.413275
C 2.144512 -0.465352 -1.865409
C 3.432020 0.242238 -1.454975
N 3.942547 -0.215088 -0.150609
C 4.832476 -1.381769 -0.365533
C 4.798728 -2.416792 0.763161
C 4.426080 0.905460 0.682237
C 5.089664 0.468726 1.989593
H 1.821788 -0.132349 -2.858739
H 1.343015 -0.251424 -1.150908
H 2.278545 -1.551820 -1.904597
H 3.245986 1.316210 -1.421205
H 4.193200 0.103212 -2.238697
H 5.346057 -2.106211 1.654735
H 5.251918 -3.350120 0.409715
H 3.765022 -2.629539 1.052651

H 4.406834 -0.146206 2.585344
H 5.333160 1.361620 2.574231
H 6.017583 -0.084585 1.845842
C 3.257927 1.823904 1.065719
H 2.708464 2.218150 0.208414
H 2.549155 1.277315 1.698309
C 6.270558 -1.036189 -0.783018
H 4.382506 -1.892752 -1.223300
H 6.855565 -0.611553 0.037315
H 6.285892 -0.320425 -1.611532
H 6.786368 -1.941977 -1.119625
H 5.163389 1.505959 0.117078
H 3.632930 2.681279 1.634609

6 References

[1] Jakob, M.; Steiner, L.; Göbel, M.; Götze, J. P.; Hopkinson, M. N. Dual *N*-Heterocyclic Carbene/Photoredox-Catalyzed Coupling of Acyl Fluorides and Alkyl Silanes. *ACS Catal.* **2024**, *14*, 17642–17653.

[2] Zarganes-Tzitzikas, T.; Neochoritis, C. G.; Stephanidou-Stephanatou, J.; Tsoleridis, C. A. Synthesis of 2-Keto-imidazoles Utilizing N-Arylamino-Substituted *N*-Heterocyclic Carbenes. *J. Org. Chem.* **2011**, *76*, 1468–1471.

[3] Zheng, Y.; Martinez-Acosta J. A.; Khimji, M.; Barbosa, L. C. A.; Clarkson, G. J.; Wills, M. Asymmetric Transfer Hydrogenation of Aryl Heteroaryl Ketones using Noyori-Ikariya Catalysts. *ChemCatChem* **2021**, *13*, 4384–4391.

[4] Kadam, S. T.; Kim, S. S. Catalyst-free silylation of alcohols and phenols by promoting HMDS in CH_3NO_2 as solvent. *Green Chem.* **2010**, *12*, 94–98.

[5] Pinkerton, F. H.; Thames, S. F. Organosilicon compounds XV. Cleavage of the silicon-carbon bond of 2-trimethylsilyl-1-methylimidazole and 2-trimethylsilyl-1-methylbenzimidazole. *J. Heterocyclic Chem.* **1972**, *9*, 67–72.

[6] Poisson, T.; Dalla, V.; Papamicaël, C.; Dupas, G.; Marsais, F.; Levacher, V. DMAP-Organocatalyzed *O*-Silyl-*O*-(or *C*-)-Benzoyl Interconversions by Means of Benzoyl Fluoride. *Synlett* **2007**, *3*, 381–386.

[7] Sterckx, H.; De Houwer, J.; Mensch, C.; Caretti, I.; Tehrani, K. A.; Herrebout, W. A.; Van Doorslaer, S.; Maes, B. U. W. Mechanism of the Cu^{II}-catalyzed benzylic oxygenation of (aryl)(heteroaryl)methanes with oxygen. *Chem. Sci.* **2016**, *7*, 346–357.

[8] Zhu, J. L.; Schull, C. R.; Tam, A. T.; Rentería-Gómez, Á.; Gogoi, A. R.; Gutierrez, O.; Scheidt, K. A. Acyl Azolium–Photoredox-Enabled Synthesis of β-Keto Sulfides. *J. Am. Chem. Soc.* **2023**, *145*, 1535–1541.

[9] Rourke, M. J.; Wang, T. C.; Schull, C. R.; Scheidt, K. A. Photoinduced Acylations Via Azolium-Promoted Intermolecular Hydrogen Atom Transfer. *ACS Catal.* **2023**, *13*, 7987–7994.

[10] Zygalski, L.; Middel, C.; Harms, K.; Koert, U. Enolizable β-Fluoroenones: Synthesis and Asymmetric 1,2-Reduction. *Org. Lett.* **2018**, *20*, 5071–5074.

[11] Huo, H.; Shen, X.; Wang, C.; Zhang, L.; Röse, P.; Chen, L.-A.; Harms, K.; Marsch, M.; Hilt, G.; Meggers, E. Asymmetric photoredox transition-metal catalysis activated by visible light. *Nature* **2014**, *515*, 100–103.

[12] Meng, Q.-Y.; Doeben, N.; Studer, A. Cooperative NHC and Photoredox Catalysis for the Synthesis of β-Trifluoromethylated Alkyl Aryl Ketones. *Angew. Chem. Int. Ed.* **2020**, *59*, 19956–19960.

[13] Samanta, R. C.; De Sarkar, S.; Froehlich, R.; Grimme, S.; Studer, A. *N*-Heterocyclic carbene (NHC) catalyzed chemoselective acylation of alcohols in the presence of amines with various acylating reagents. *Chem. Sci.* **2013**, *4*, 2177–2184.

[14] Aranzaes, J. R.; Daniel, M.-C.; Astruc, D. Metallocenes as references for the determination of redox potentials by cyclic voltammetry – Permethylated iron and cobalt sandwich complexes, inhibition by polyamine dendrimers, and the role of hydroxy-containing ferrocenes. *Can. J. Chem.* **2006**, *84*, 288–299.

[15] Silvi, M.; Verrier, C.; Rey, Y. P.; Buzzetti, L.; Melchiorre, P. Visible-light excitation of iminium ions enables the enantioselective catalytic β-alkylation of enals. *Nature Chem.* **2017**, *9*, 868–873.

[16] Ren, S.-C.; Lv, W.-X.; Yang, X.; Yan, J.-L.; Xu, J.; Wang, F.-X.; Hao, L.; Chai, H.; Jin, Z.; Chi, Y. R. Carbene-Catalyzed Alkylation of Carboxylic Esters via Direct Photoexcitation of Acyl Azolium Intermediates. *ACS Catal.* **2021**, *11*, 2925–2934.

[17] Rehm, D.; Weller, A. Kinetics of Fluorescence Quenching by Electron and H-Atom Transfer. *Isr. J. Chem.* **1970**, *8*, 259–271.
[18] Pracht, P.; Bohle, F.; Grimme, S. Automated exploration of the low-energy chemical space with fast quantum chemical methods. *Phys. Chem. Chem. Phys.* **2020**, *22*, 7169–7192.
[19] Hanwell, M. D.; Curtis, D. E.; Lonie, D. C.; Vandermeersch, T.; Zurek, E.; Hutchinson, G. R. Avogadro: an advanced semantic chemical editor, visualization, and analysis platform. *J. Cheminform.* **2012**, *4*, 17.
[20] Ehlert, S.; Stahn, M.; Spicher, S.; Grimme, S. Robust and Efficient Implicit Solvation Model for Fast Semiempirical Methods. *J. Chem. Theory Comput.* **2021**, *17*, 4250–4261.
[21] Bannwarth, C.; Ehlert, S.; Grimme, S. GFN2-xTB–An Accurate and Broadly Parametrized Self-Consistent Tight-Binding Quantum Chemical Method with Multipole Electrostatics and Density-Dependent Dispersion Contributions. *J. Chem. Theory Comput.* **2019**, *15*, 1652–1671.
[22] Frisch, M. J.; Trucks, G. W.; Schlegel, H. B.; Scuseria, G. E. ; Robb, M. A.; Cheeseman, J. R.; Scalmani, G.; Barone, V.; Petersson, G. A.; Nakatsuji, H.; Li, X.; Caricato, M.; Marenich, A. V.; Bloino, J.; Janesko, B. G.; Gomperts, R.; Mennucci, B.; Hratchian, H. P.; Ortiz, J. V.; Izmaylov, A. F.; Sonnenberg, J. L.; Williams-Young, D.; Ding, F.; Lipparini, F.; Egidi, F.; Goings, J.; Peng, B.; Petrone, A.; Henderson, T.; Ranasinghe, D.; Zakrzewski, V. G.; Gao, J.; Rega, N.; Zheng, G.; Liang, W.; Hada, M.; Ehara, M.; Toyota, K.; Fukuda, R.; Hasegawa, J.; Ishida, M.; Nakajima, T.; Honda, Y.; Kitao, O.; Nakai, H.; Vreven, T.; Throssell, K.; Montgomery, Jr., J. A.; Peralta, J. E.; Ogliaro, F.; Bearpark, M. J.; Heyd, J. J.; Brothers, E. N.; Kudin, K. N.; Staroverov, V. N.; Keith, T. A.; Kobayashi, R.; Normand, J.; Raghavachari, K.; Rendell, A. P.; Burant, J. C.; Iyengar, S. S.; Tomasi, J.; Cossi, M.; Millam, J. M.; Klene, M.; Adamo, C.; Cammi, R.; Ochterski, J. W.; Martin, R. L.; Morokuma, K.; Farkas, O.; Foresman, J. B.; Fox, D. J. Gaussian 16, Revision C.01. *J. Chem. Theory Comput.* **2016**, *Gaussian Inc. Wallingford CT.*
[23] Mavroskoufis, A.; Lohani, M.; Weber, M.; Hopkinson, M. N.; Götze, J. P. A (TD-)DFT study on photo-NHC catalysis: photoenolization/Diels-Alder reaction of acid fluorides catalyzed by *N*-heterocyclic carbenes. *Chem. Sci.* **2023**, *14*, 4027–4037.
[24] Runge, E.; Gross, E. K. U. Density-Functional Theory for Time-Dependent Systems. *Phys. Rev. Lett.* **1984**, *52,* 997–1000.
[25] Yanai, T.; Tew, D. P.; Handy, N. C. A new hybrid exchange-correlation functional using the Coulomb-attenuating method (CAM-B3LYP). *Chem. Phys. Lett.* **2004**, *393*, 51–57.
[26] Hariharan, P. C.; Pople, J. A. The influence of polarization functions on molecular orbital hydrogenation energies. *Theor. Chem. Acc.* **1973**, *28*, 213–222.
[27] Hehre, W. J.; Ditchfield, R.; Pople, J. A. Self–Consistent Molecular Orbital Methods. XII. Further Extensions of Gaussian–Type Basis Sets for Use in Molecular Orbital Studies of Organic Molecules. *J. Chem. Phys.* **1972**, *56*, 2257–2261.
[28] Ditchfield, R.; Hehre, W. J.; Pople, J. A. Self–Consistent Molecular-Orbital Methods. IX. An Extended Gaussian-Type Basis for Molecular-Orbital Studies of Organic Molecules. *J. Chem. Phys.* **1971**, *54*, 724–728.
[29] Cossi, M.; Rega, N.; Scalmani, G.; Barone, V. Energies, structures, and electronic properties of molecules in solution with the C-PCM solvation model. *J. Comput. Chem.* **2003**, *24*, 669–681.
[30] Hermann, G.; Pohl, V.; Tremblay, J. C.; Paulus, B.; Hege, H.-C.; Schild, A. ORBKIT: A modular python toolbox for cross-platform postprocessing of quantum chemical wavefunction data. *J. Comput. Chem.* **2016**, *37*, 1511–1520.

7 NMR spectra of novel compounds

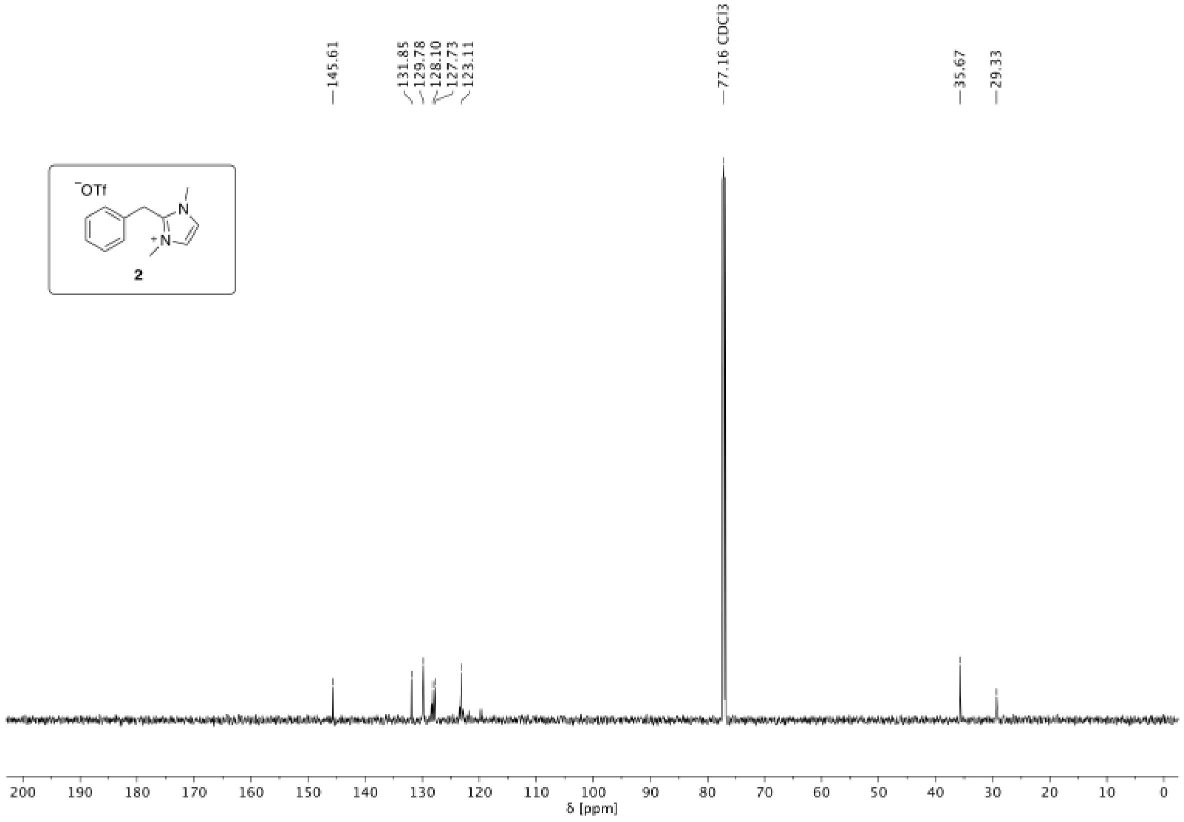

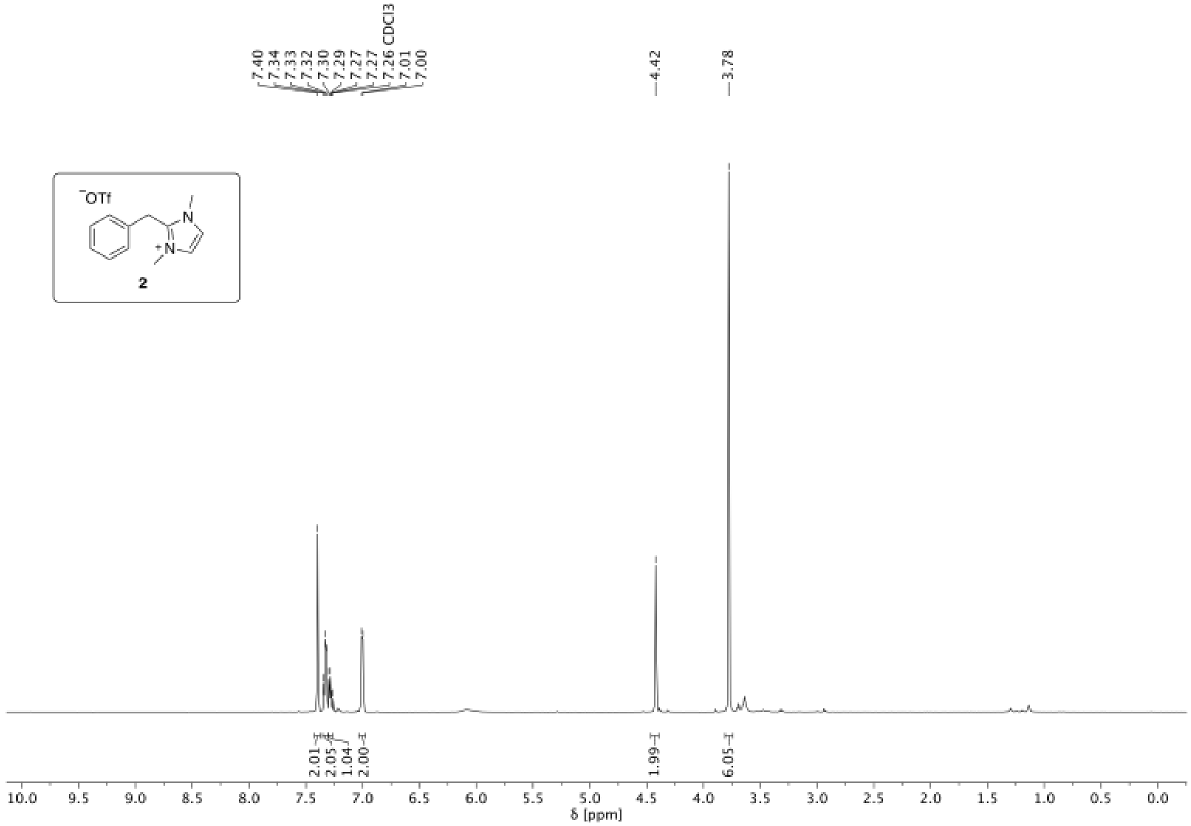

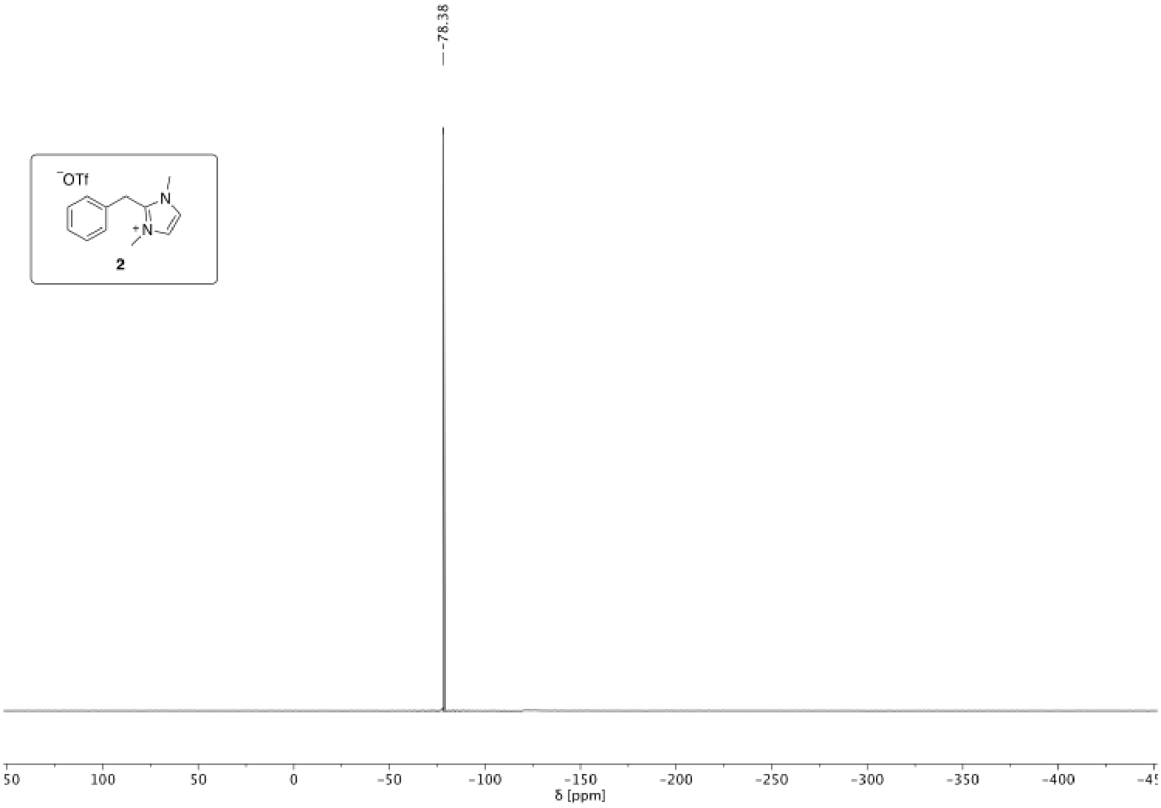
−78.38
⁻OTf
N
+N
2
150
100
50
0
−50
−100
−150
δ [ppm]
−200
−250
−300
−350
−400

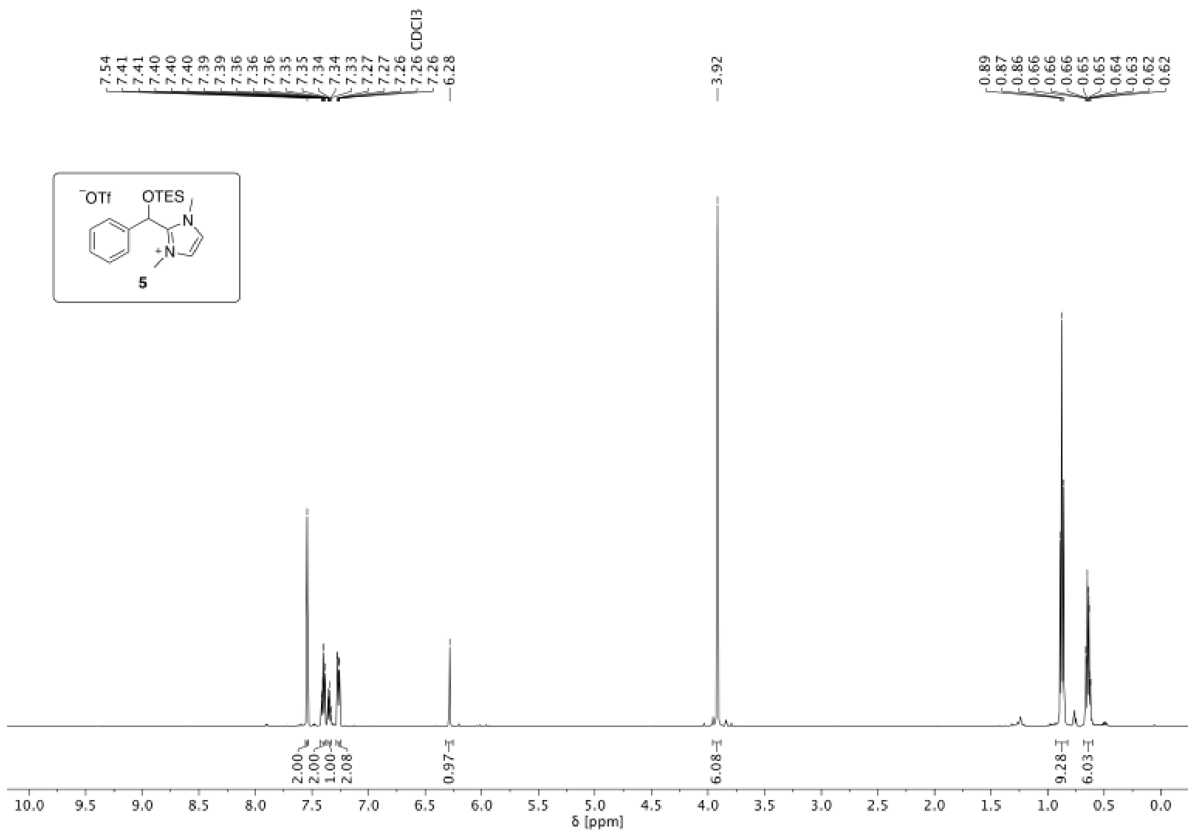
7.54
7.41
7.41
7.40
7.40
7.39
7.39
7.36
7.36
7.35
7.35
7.34
7.34
7.33
7.27
7.27
7.26
7.26 CDCl3
7.26
6.28
3.92
0.89
0.87
0.86
0.66
0.66
0.66
0.65
0.65
0.64
0.63
0.62
0.62
-OTf
OTES
N
+N
5
2.00
2.00
1.00
2.08
0.97
6.08
9.28
6.03
δ [ppm]

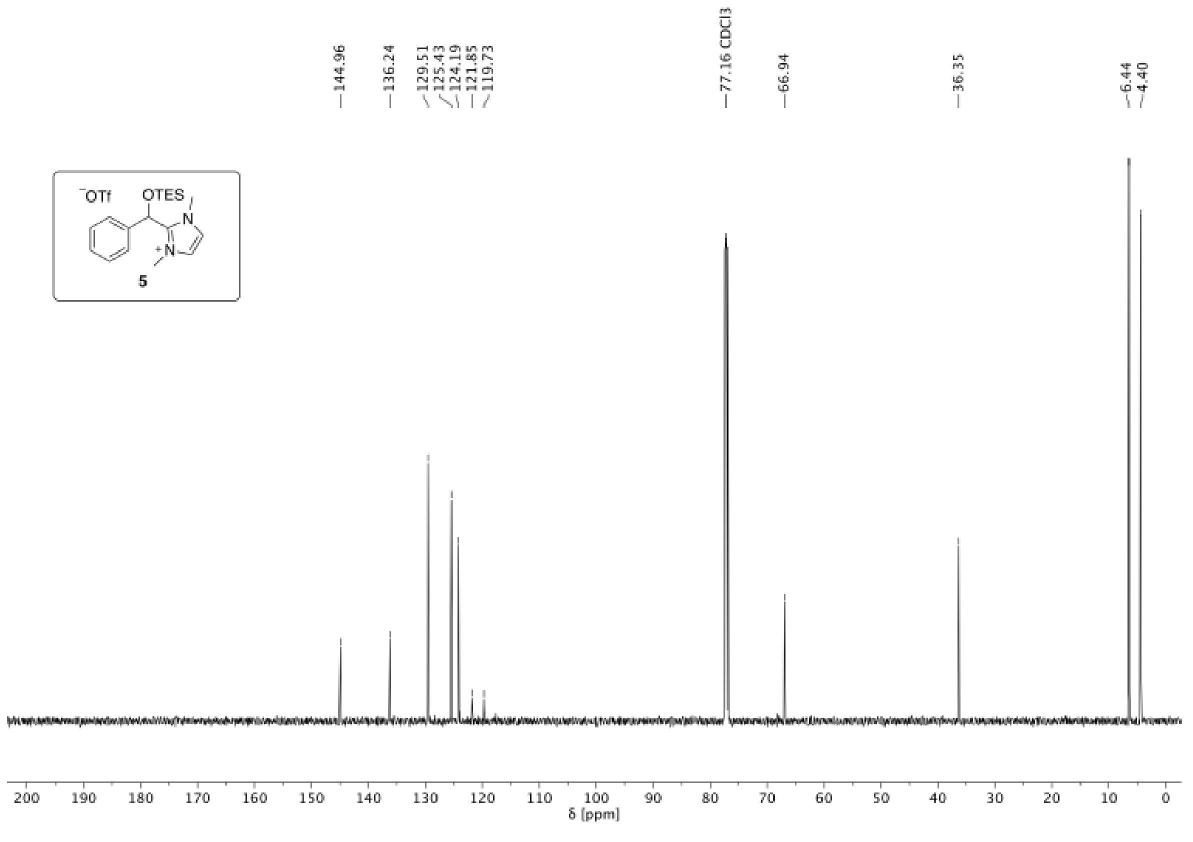
144.96
136.24
129.51
125.43
124.19
121.85
119.73
77.16 CDCl3
66.94
36.35
6.44
4.40
-OTf
OTES
N
+N
5
δ [ppm]

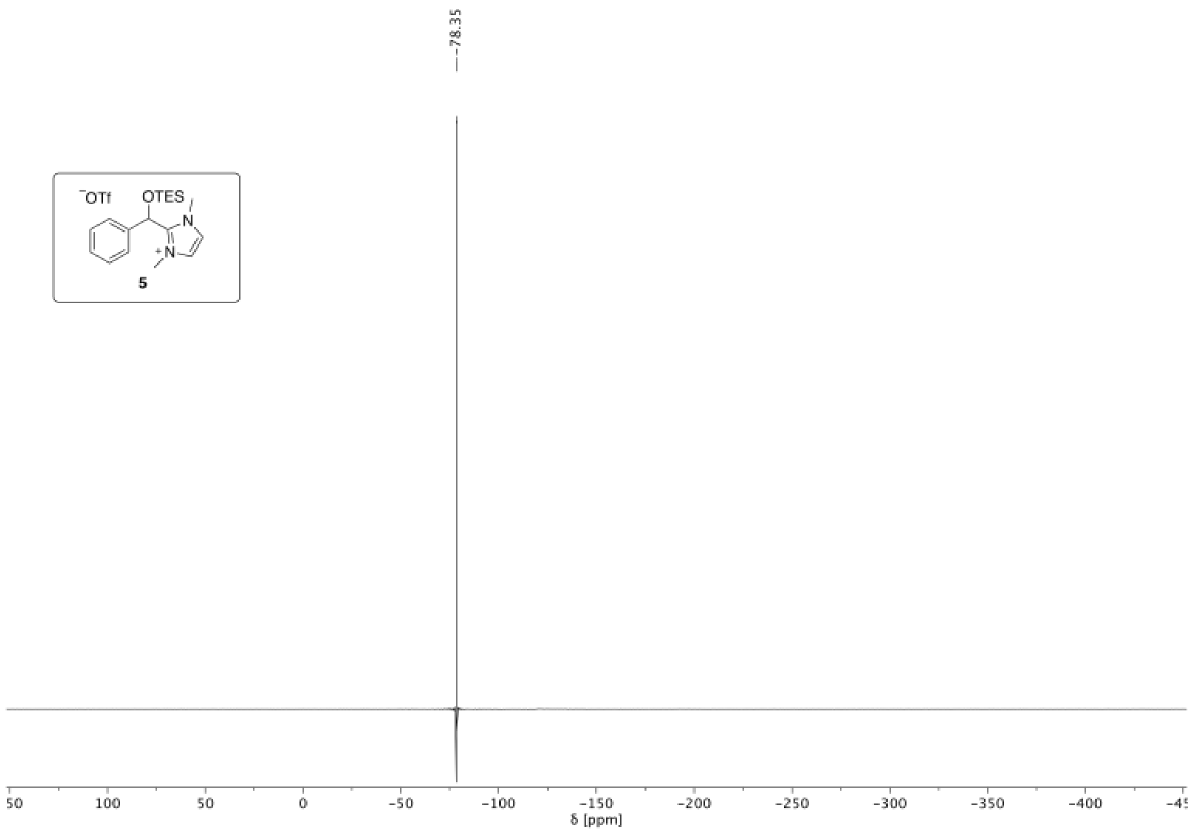
−78.35
⁻OTf
OTES
N
N
5
δ [ppm]

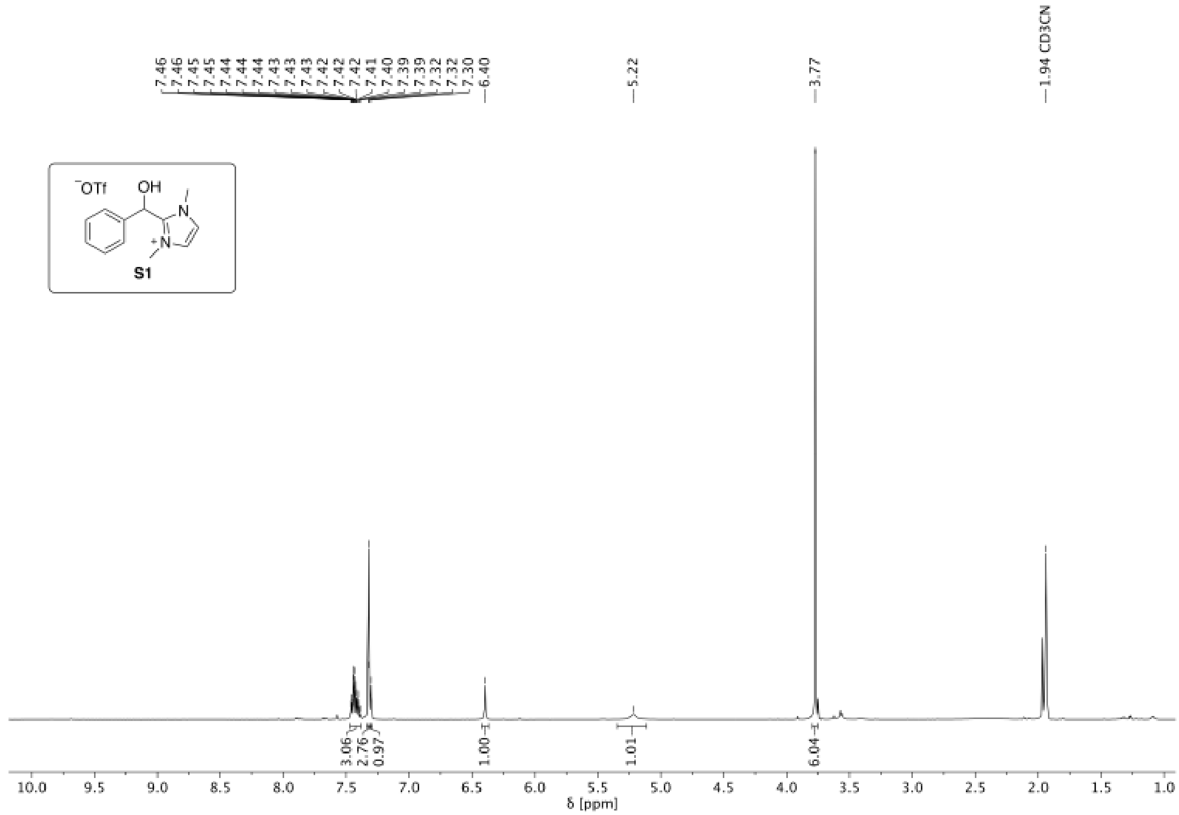

7.46
7.46
7.45
7.45
7.44
7.44
7.44
7.43
7.43
7.43
7.42
7.42
7.42
7.41
7.40
7.39
7.39
7.32
7.32
7.30
6.40
5.22
3.77
1.94 CD3CN
−OTf
OH
N
+
N
S1
3.06
2.76
0.97
1.00
1.01
6.04
10.0
9.5
9.0
8.5
8.0
7.5
7.0
6.5
6.0
5.5
5.0
4.5
4.0
3.5
3.0
2.5
2.0
1.5
1.0
δ [ppm]

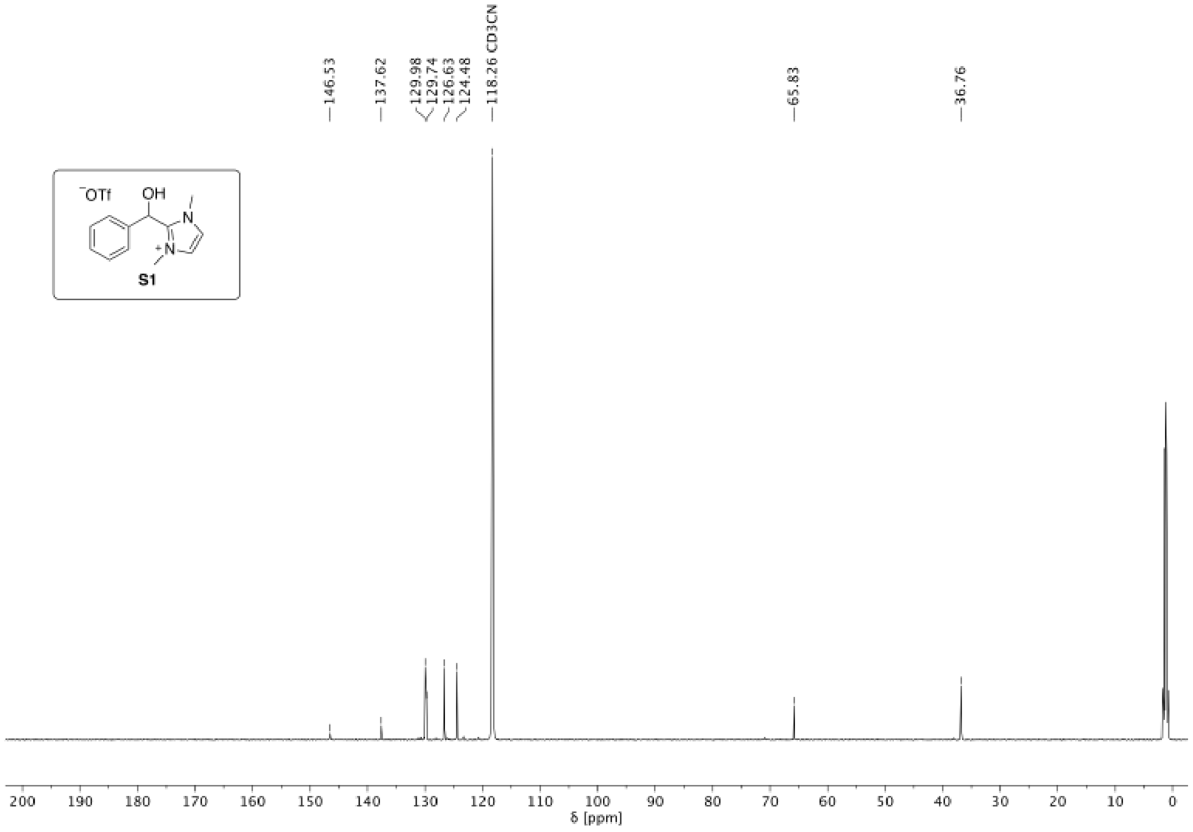

146.53
137.62
129.98
129.74
126.63
124.48
118.26 CD3CN
65.83
36.76
−OTf
OH
N
+
N
S1
200
190
180
170
160
150
140
130
120
110
100
90
80
70
60
50
40
30
20
10
0
δ [ppm]

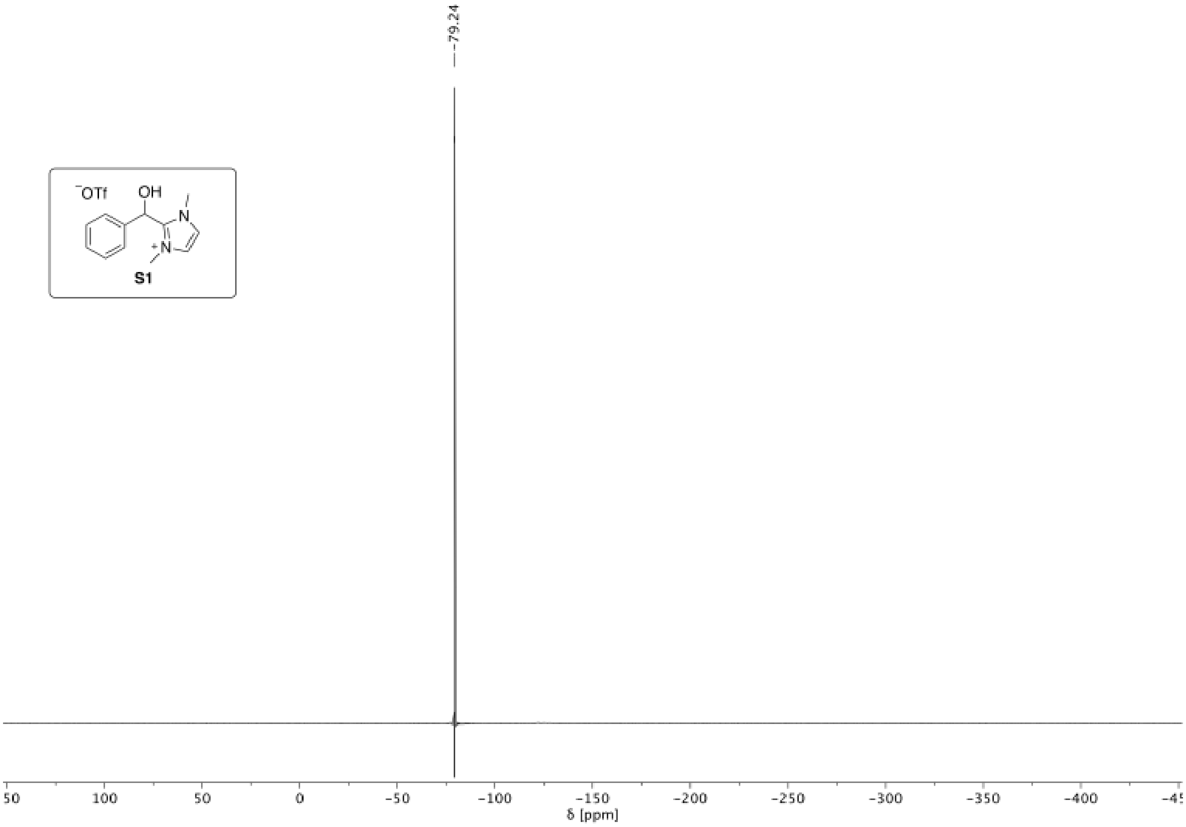
-79.24
⁻OTf
OH
N
N
S1
50
100
50
0
−50
−100
−150
−200
−250
−300
−350
−400
δ [ppm]

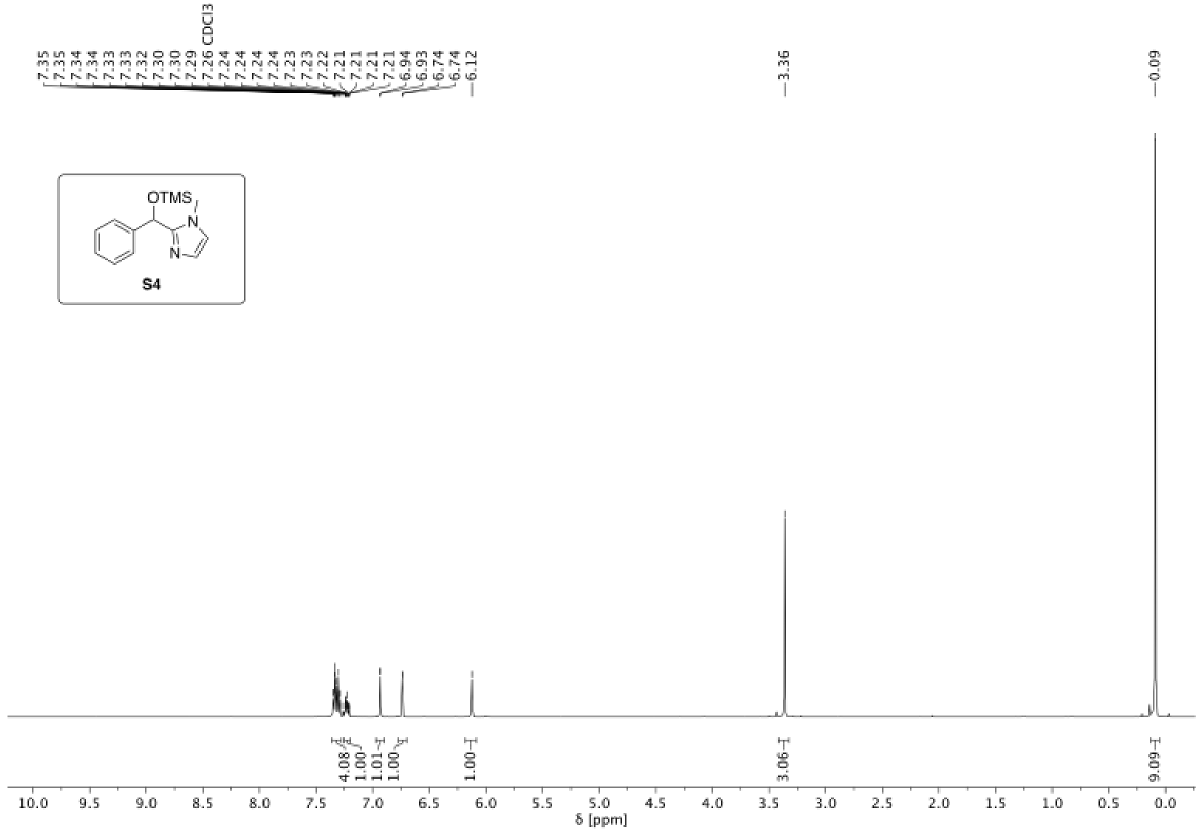
7.35
7.35
7.34
7.34
7.33
7.33
7.32
7.30
7.30
7.29
7.26 CDCl3
7.24
7.24
7.24
7.24
7.23
7.23
7.22
7.21
7.21
7.21
7.21
6.94
6.93
6.74
6.12
3.36
0.09
OTMS
N
N
S4
4.08
1.00
1.01
1.00
1.00
3.06
9.09
10.0
9.5
9.0
8.5
8.0
7.5
7.0
6.5
6.0
5.5
5.0
4.5
4.0
3.5
3.0
2.5
2.0
1.5
1.0
0.5
0.0
δ [ppm]

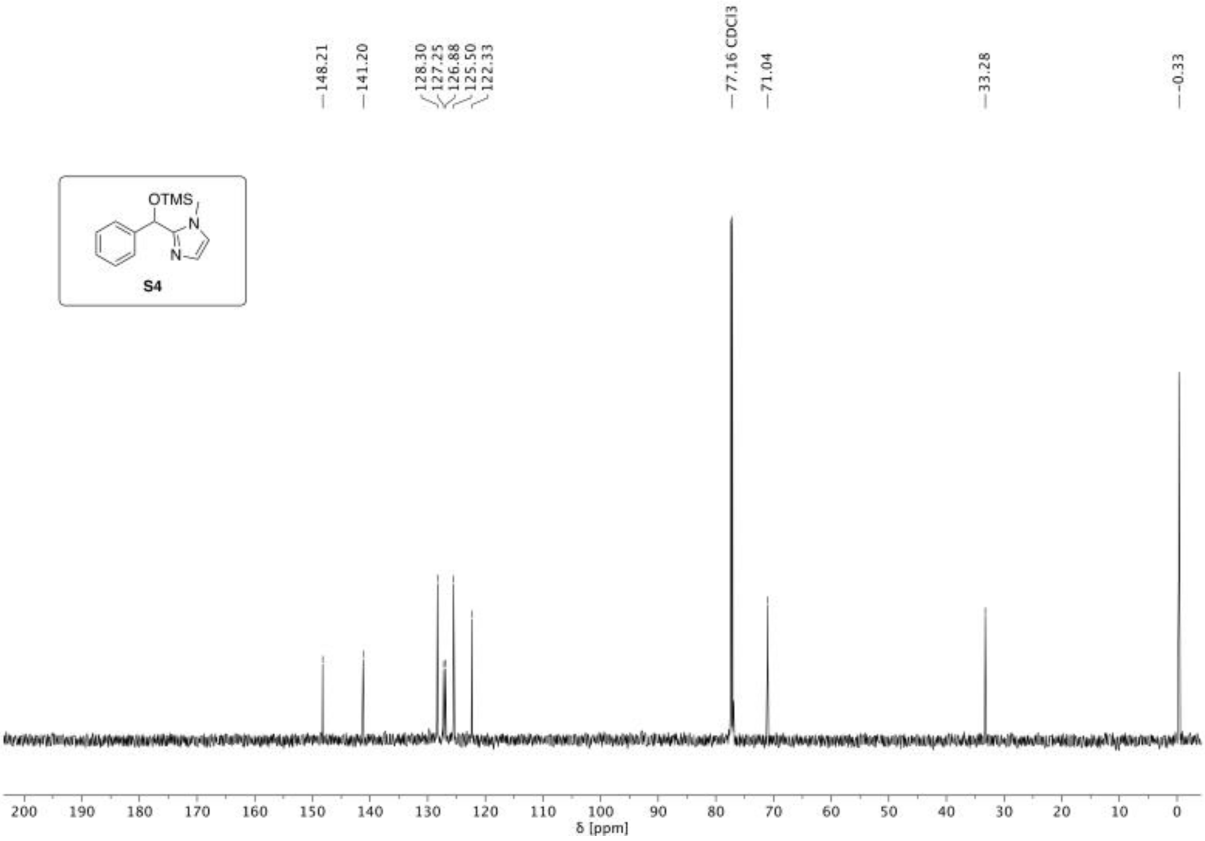
148.21
141.20
128.30
127.25
126.88
125.50
122.33
77.16 CDCl3
71.04
33.28
-0.33
OTMS
N
N
S4
200
190
180
170
160
150
140
130
120
110
100
90
80
70
60
50
40
30
20
10
0
δ [ppm]

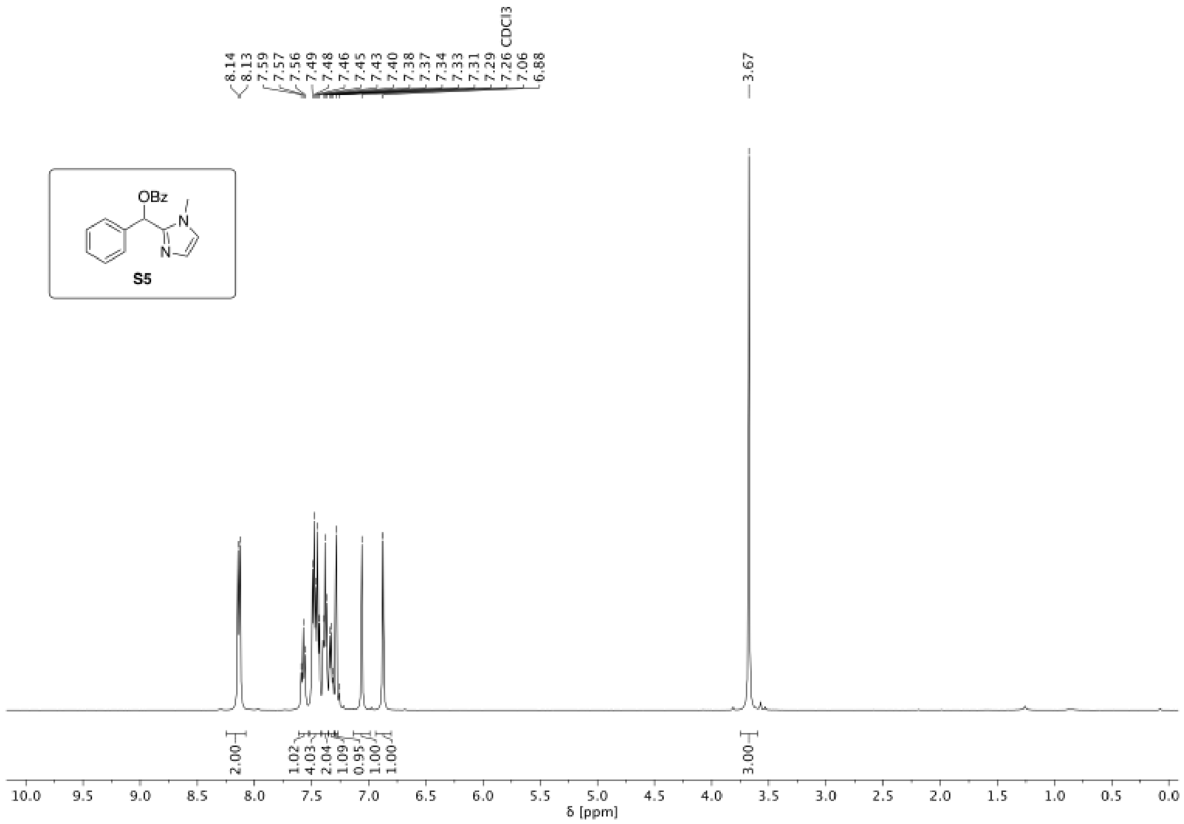
8.14
8.13
7.59
7.57
7.56
7.49
7.48
7.46
7.45
7.43
7.40
7.38
7.37
7.34
7.33
7.31
7.29
7.26 CDCl3
7.06
6.88
3.67
OBz
N
N
S5
2.00
1.02
4.03
2.04
1.09
0.95
1.00
1.00
3.00
δ [ppm]

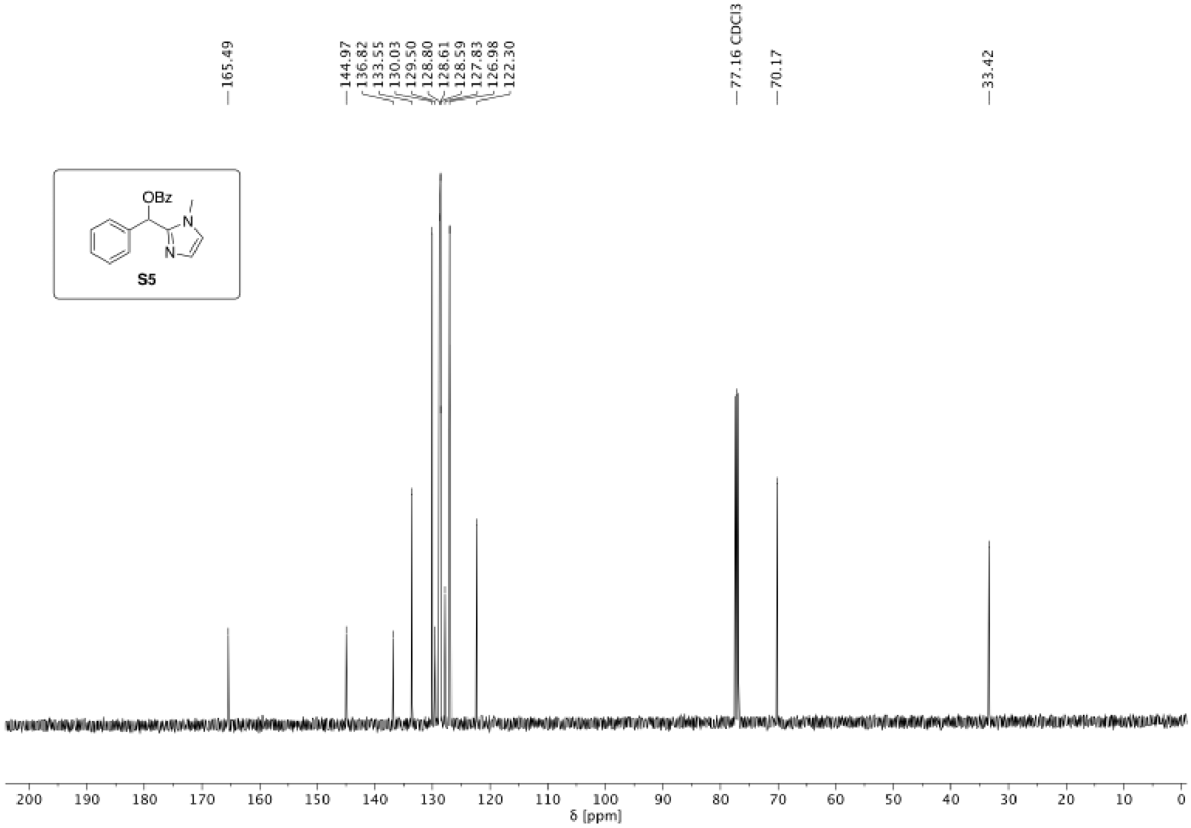
165.49
144.97
136.82
133.55
130.03
129.50
128.80
128.61
128.59
127.83
126.98
122.30
77.16 CDCl3
70.17
33.42
OBz
N
N
S5
δ [ppm]
